# Targeting the PRMT5/Nur77 methylation axis enhances endometrial decidualization capacity and female fertility in preclinical models

**DOI:** 10.1172/JCI178862

**Published:** 2026-07-16

**Authors:** Zhiwen Cao, Xinyu Cai, Jie Mei, Na Kong, Yang Liu, Xiaoyue Shen, Min Wu, Xin Zhen, Jianxin Sun, Rong Li, Ruiwei Jiang, Haixiang Sun, Guijun Yan

**Affiliations:** 1 Center for Reproductive Medicine and Obstetrics and Gynecology, Nanjing Drum Tower Hospital, Affiliated Hospital of Medical School, State Key Laboratory of Pharmaceutical Biotechnology, and; 2 Center for Molecular Reproductive Medicine, Nanjing University, Nanjing, Jiangsu, China.; 3 State Key Laboratory of Reproductive Medicine and Offspring Health, Nanjing Medical University, Nanjing, Jiangsu, China.; 4 Department of Medicine, Center for Translational Medicine, Thomas Jefferson University, Philadelphia, Pennsylvania, USA.; 5 Jiangsu Human Reproductive Function Remodeling Engineering Research Center, Nanjing, Jiangsu, China.; 6 Nanjing Clinical Medical Center for Reproductive Medicine, Nanjing, Jiangsu, China.

**Keywords:** Genetics, Reproductive biology, Fertility, Peptides

## Abstract

Defective endometrial decidualization is one major cause of female infertility, yet the underlying mechanisms remain elusive. Here, we identified that protein arginine methyltransferase 5 (PRMT5), which was upregulated during decidualization and by progesterone stimulation, was markedly downregulated in the endometria of patients with recurrent implantation failure (RIF), along with a global reduction of symmetric dimethylarginine (SDMA). Uterine stroma-specific ablation of *Prmt5* in mice severely impaired decidualization, leading to infertility. A multiomics analysis in human endometrial stromal cells (EnSCs) revealed that PRMT5 promoted decidualization primarily by catalyzing SDMA at arginine 346 (R346) of the orphan nuclear receptor Nur77, which directs its proper chromatin occupancy. Targeting the PRMT5/Nur77 methylation axis, we designed a peptide, Pep-Nur77^R346K^, which rescued the decidualization of multiple preclinical models: PRMT5-deficient human EnSCs, both genetic knockout (*Prmt5^d/d^*) and pharmacologically inhibited mouse models, and most importantly, primary RIF EnSCs. In a retrospective cohort of 114 participants, the correlated reductions of endometrial PRMT5/Nur77-R346me2s were confirmed, which demonstrated robust predictive value for pregnancy outcome. Our work establishes the PRMT5/Nur77 methylation axis as a key regulator of endometrial receptivity and highlights both a diagnostic biomarker and a peptide-based therapeutic potential for infertility.

## Abstract 2

Protein methylation in human endometrium is critical for female fertility. A de novo peptide targeting the methylation axis can improve female fertility.

## Graphical abstract

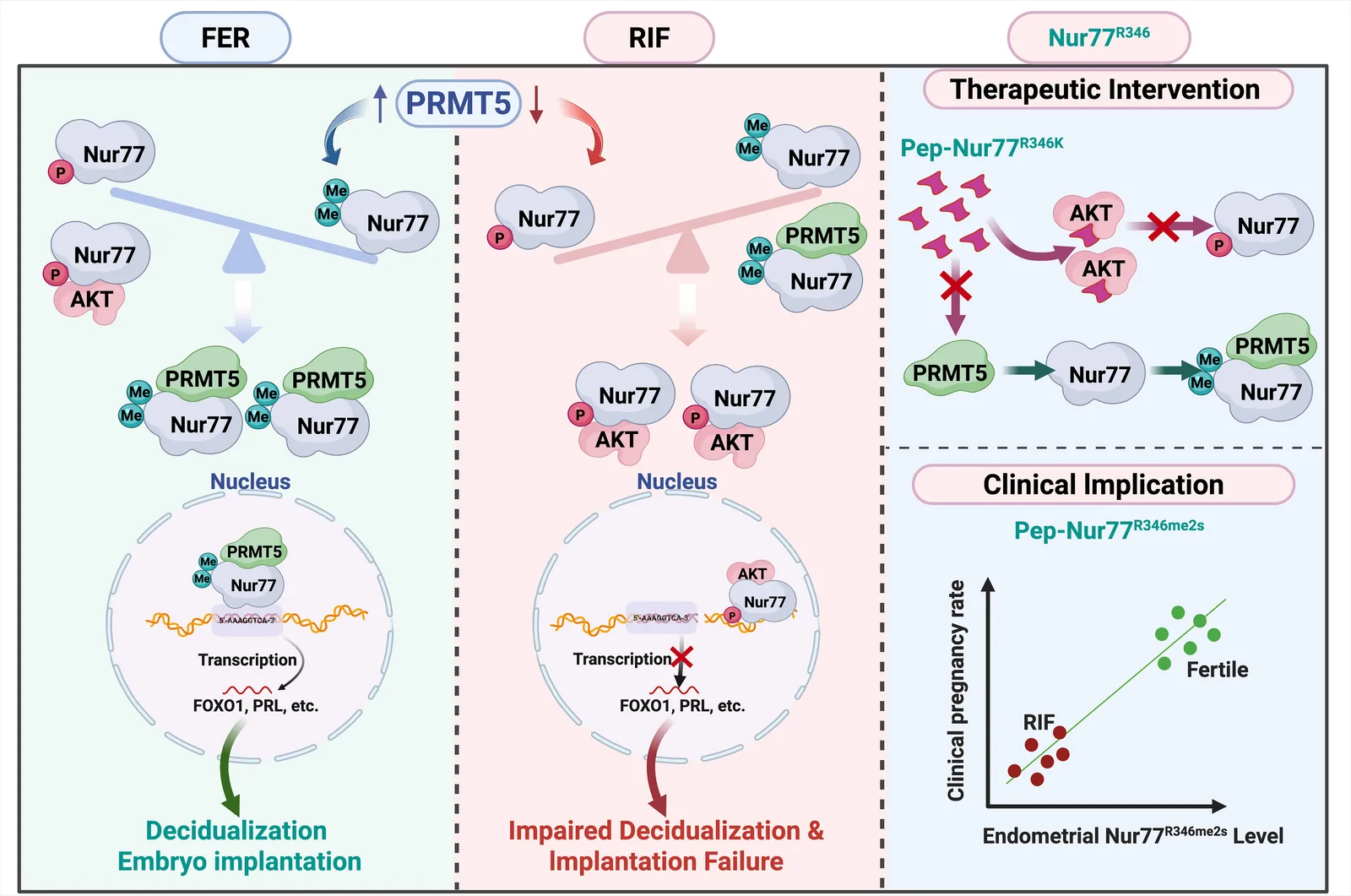

## Introduction

Infertility is a global reproductive health issue that affects 1 in 6 couples (1, 2). The number of women undergoing assisted reproductive technology (ART) has risen steadily (3). However, even with preimplantation genetic testing to ensure embryonic quality, the delivery rate per transfer remains at approximately 48.7%, emphasizing the critical contribution of endometrial dysfunction to fertility outcomes (4). Decidualization, referring to the transformation of endometrial stromal cells (EnSCs) from spindle-shaped fibroblasts into rounded, secretory epithelioid cells, is the major change in the maternal endometrium that is essential for pregnancy health (5). Recurrent implantation failure (RIF) is characterized by implantation failure after multiple embryo transfers and represents a clinically devastating outcome for women receiving embryo transfer (6). However, its underlying mechanism remains poorly understood (7). Emerging evidence indicated decidualization defects predispose women to RIF (8, 9). Therefore, deciphering the pathological mechanism underlying decidual incompetence highlights a promising avenue for overcoming the bottleneck of RIF during clinical practice.

Decidualization is tightly regulated by the ovarian hormone progesterone (10), which governs an intricate transcriptional regulatory network consisting of major transcription factors, such as FOXO1 (11), HOXA10 (12), and notably, the orphan nuclear receptor Nur77 (13, 14). In addition to regulation at the expression level, the activity of these key transcription factors is further controlled by a dynamic interplay of posttranslational modifications (PTMs). Phosphorylation (15, 16), ubiquitination (17, 18), and acetylation (19, 20) fine-tune the stability, localization, and DNA binding specificity of multiple decidual regulators. More interestingly, we have reported that the homeobox transcription factor HOXA10, a well-established master regulator of receptivity, undergoes acetylation (21), SUMOylation (22), and phosphorylation (23), and these modifications crosstalk to precisely modulate its transcriptional activity and protein interactions during the implantation window. PTMs exponentially expand the diversity of protein functions with both reversible and irreversible changes (24) and are considered better therapeutic targets than the mostly “undruggable” transcription factors (25, 26).

Protein arginine methylation is emerging as one attractive therapeutic target in fields such as immunology, neuroscience, and oncology (27). Protein arginine methyltransferase (PRMT) family can be categorized into 3 types: type I, PRMT1, 2, 3, 4, 6, and 8, catalyzes monomethylarginine (MMA) and asymmetric dimethylarginine (ADMA); type II, PRMT5 and PRMT9, generates MMA and symmetric dimethylation (SDMA); and type III, PRMT7, only regulates MMA (28). Among them, PRMT5 is the primary SDMA methyltransferase in eukaryotic species (29). PRMT5 is essential for multiple cellular processes, including transcriptional regulation (30, 31), RNA metabolism (32, 33), DNA damage repair (34, 35), and chromatin remodeling (36). Functional studies show that PRMT5 plays essential regulatory roles in various lineage-specific differentiation programs, such as myelin formation during nervous system development (37), maintaining the balance of self-renewal and differentiation in hematopoietic stem cells (38), and regulating the phenotypic plasticity of vascular smooth muscle cells (39). Notably, PRMT5 dysfunction is closely associated with pathogenic mechanisms of various human diseases, including neurodegenerative disorders, malignancies, and autoimmune diseases (40). We previously reported that PRMT5 was reduced in the endometrium of women with endometriosis and adenomyosis and may contribute to the associated fertility defects (41, 42). The critical role of PRMT5 in decidualization is implicated by treatment with a PRMT5 inhibitor, which downregulated the key decidual markers FOXO1, HOXA10, and WNT4 and induced nuclear translocation of P65, collectively resulting in decidualization defects (42). However, a systematic investigation of PRMT5 in the endometrium of RIF patients and an in-depth exploration of the functional roles of PRMT5 in endometrial decidualization are still lacking.

In this study, we systematically profiled the endometrial transcriptome and arginine methylome across the window of implantation (WOI) and comprehensively compared these profile changes in RIF patients and PRMT5-deficient EnSCs. Together, these results establish PRMT5 as a progesterone-induced decidual regulator that acted via SDMA modification. Stroma-specific PRMT5-knockout mice validated its essential role in endometrial decidualization in vivo. Integrated multiomics analysis revealed PRMT5-mediated SDMA modification of the orphan nuclear receptor Nur77 at R346 promoted decidualization by directing the precise chromatin occupancy and recruiting appropriate transcriptional chaperones. More importantly, we designed a functional R346 mimetic decoy peptide (Pep-Nur77^R346K^) that rescues decidualization defects and restores pregnancy outcomes in multiple preclinical models. The endometrial Nur77-R346me2s levels displayed a robust predictive capability for pregnancy outcomes in a retrospective investigation of women with normal fertility and RIF. Our findings establish the PRMT5/Nur77 methylation axis as a key regulator of endometrial receptivity and suggest a targeted therapeutic strategy for infertility.

## Results

### PRMT5-mediated SDMA protein modification is compromised in RIF.

**Figure 1:**
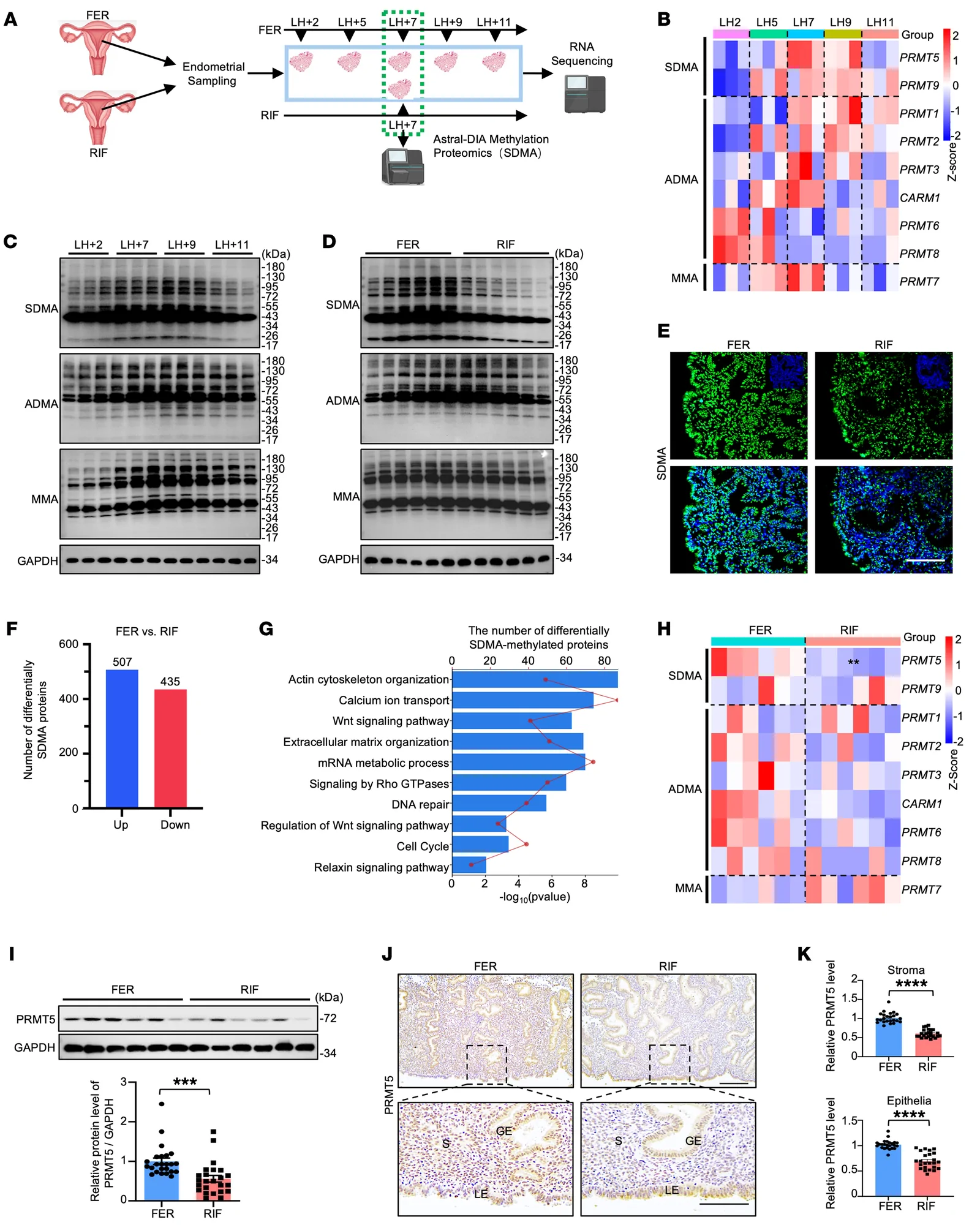
PRMT5-mediated arginine methylome is compromised in RIF. (**A**) Schematic diagram illustrating the collection of endometrium samples for transcriptome sequencing at different time points during the secretory phase of LH in normal FER, as well as Alstra-DIA methylation sequencing of endometrial samples at the LH+7 time point from both controls and patients with RIF. (**B**) Transcriptomic analysis reveals the expression dynamics of PRMT family expression profiles at different time points during the secretory phase. (**C** and **D**) Western blot analysis revealing expression patterns of 3 protein arginine methylation modifications (SDMA, ADMA, MMA) in human endometrium temporal dynamics from LH+2 to LH+11 days during secretory phase (**C**) (*n* = 3) and between FER and RIF patients (**D**) (*n* = 6). (**E**) Immunofluorescence detection of SDMA expression and localization in midsecretory-phase endometrium from FER and RIF patients. (**F**) The number of proteins with differentially expressed SDMA-modified peptides in RIF compared with FER endometria, using Alstra-DIA methylation sequencing and co-immunoprecipitation of anti-SDMA antibody. (**G**) Biological Process (Gene Ontology) enrichment analysis of differentially methylated proteins in RIF compared with FER groups. (**H**) Heatmap of PRMT family expression profiles in FER and RIF endometria at LH+7 (*n* = 6). Western blot (**I**) analysis of PRMT5 protein levels and IHC staining (**J**) and IHC quantitative analysis (**K**) of PRMT5 expression in endometrial epithelial and stromal cells in FER and RIF patients (*n* = 24). Data represent mean ± SEM. **P < 0.01; ***P < 0.001; ****P < 0.0001; Student’s *t* test. Black and white scale bars: 100 μm. FER, fertile controls; RIF, recurrent implantation failure; DIA, data-independent acquisition; SDMA, symmetric dimethylarginine; ADMA, asymmetric dimethylarginine; MMA, monomethylarginine; LH, luteinizing hormone; GE, glandular epithelium; LE, luminal epithelium; S, stroma.

Dynamic transcriptomic changes during the WOI are critical for establishing the endometrial receptivity (43), we first conducted longitudinal RNA-seq on endometrial biopsies from fertile women (FER) across the secretory phase (LH+2, LH+5, LH+7, LH+9, LH+11) (Figure 1A and Supplemental Tables 1 and 2; supplemental material available online with this article; https://doi.org/10.1172/JCI178862DS1). As previously reported (44), progesterone receptor expression was high in epithelial cells during the early secretory phase (LH+2 to LH+5), declined by the midsecretory phase (LH+7 to LH+9), and further decreased by the late secretory phase (LH+11), but persisted at the stroma throughout the stages, coinciding with the temporal mRNA changes of proliferation, differentiation, and decidualization representative genes (Supplemental Figure 1, A–C, and Supplemental Table 1). PRMT family genes exhibited distinct expression patterns: an early secretory group (*PRMT6/8*) that declined at WOI onset, a midsecretory transient group (*PRMT3/7*) peaking at LH+7, and a late-accumulating group (*PRMT1/2* and type II enzymes *PRMT5/9*) that was maximized from LH+7 to LH+9 (Figure 1B). Accordingly, a moderate increase of ADMA and an obvious increase of MMA was observed from LH+2 to LH+7, but only SDMA showed a robust accumulation during the WOI from LH+7 to LH+9 across a broad molecular weight range (17–180 kDa) (Figure 1C), indicating a potential association with endometrial receptivity. Interestingly, the analysis of one published single-cell RNA-seq dataset of human endometrium also indicates that PRMT5 was the only SDMA catalytic enzyme that expressed much higher levels in the EnSCs at LH+7 compared with LH+2 and LH+12 (Supplemental Figure 1D), implicating its critical role in regulating SDMA during endometrial decidualization.

Here, we collected endometrial biopsies from 6 patients with RIF, who did not achieve a pregnancy after at least 5 transfers of high-quality embryos, for in-depth multiomics analysis to identify the key endometrial factors implicated in the pathogenesis of RIF. We found the global SDMA abundance was markedly reduced in the LH+7 endometria from patients with RIF, in contrast with comparable ADMA and MMA signals (Figure 1D). The reduction of SDMA was more pronounced in the endometrial stroma than in the epithelium (Figure 1E and Supplemental Figure 2A). These findings suggest that the diminished endometrial SDMA modification may contribute to the pathogenesis of RIF.

Next, by integrating a PTMScan SDMA motif enrichment kit with Alstra-DIA proteomics, we obtained a high-sensitivity SDMA protein landscape in the pooled control and RIF endometrial samples mid-secretory (LH+7) (Supplemental Figure 2B and Supplemental Table 3). There were 507 upregulated and 435 downregulated SDMA-modified proteins in patients with RIF (Figure 1F). These differentially methylated proteins are prominently enriched in pathways essential for decidualization, including Actin cytoskeleton organization, Relaxin signaling, and Wnt signaling (Figure 1G). Transcriptomic analysis demonstrated multiple dysregulated decidualization pathways in these RIF endometria (Supplemental Figure 2C and Supplemental Table 4). Between the 2 primary enzymes responsible for SDMA modification, PRMT9 expression was unaltered, whereas PRMT5 was downregulated in RIF endometrium (Figure 1H), which was further confirmed by qPCR (Supplemental Figure 2D). The downregulation of PRMT5 proteins was detected in both endometrial compartments of RIF patients but was more pronounced at the stroma (Figure 1, I–K), which is consistent with the SDMA staining results (Figure 1E). Collectively, these results identify endometrial PRMT5 deficiency, particularly in the stroma of patients with RIF, which might account for the hypo-SDMA of decidual regulatory networks.

### PRMT5 deficiency impairs endometrial decidualization in vivo.

**Figure 2:**
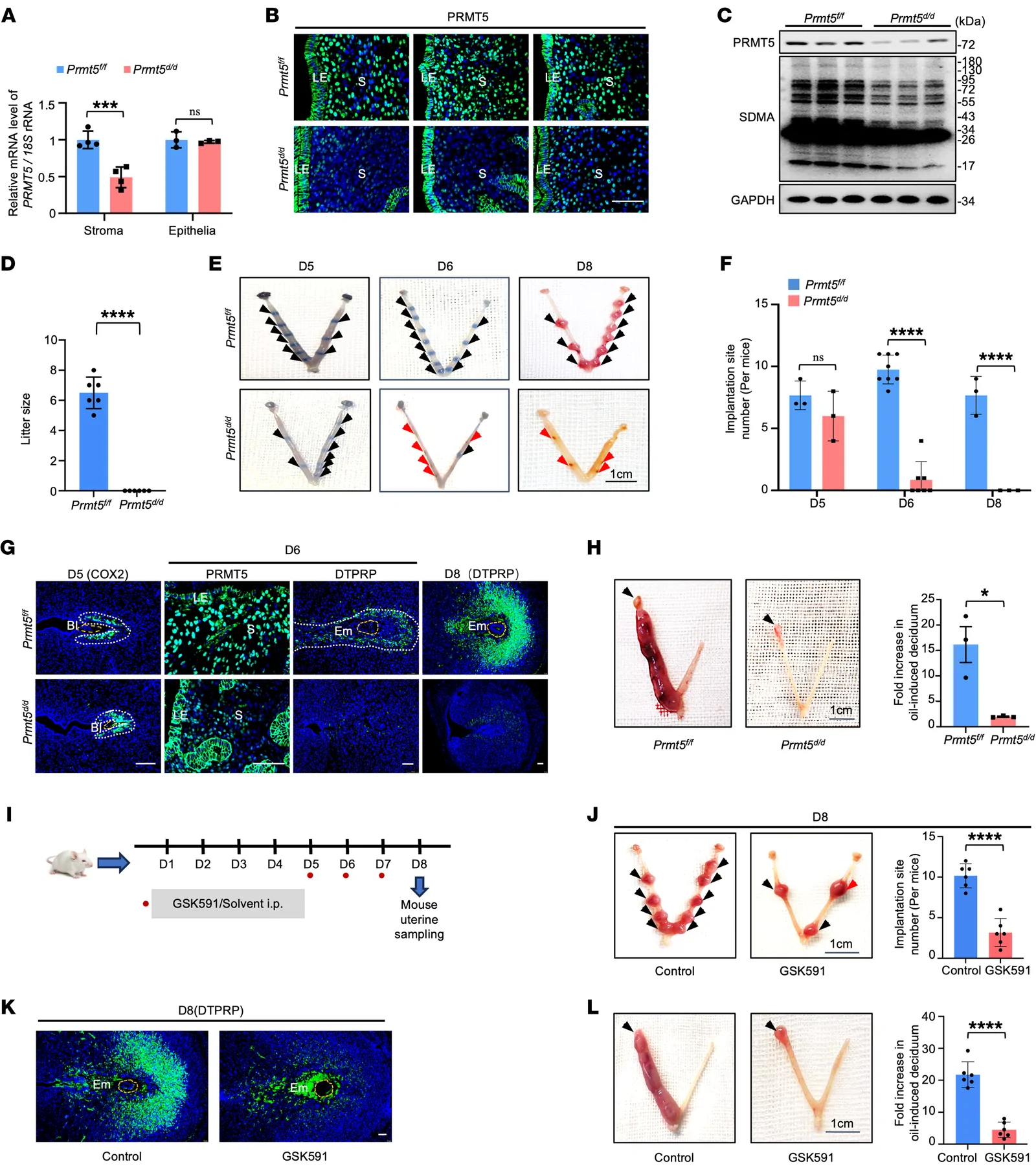
Stromal PRMT5 deficiency impairs endometrial decidualization and embryo development. (**A**) qRT-PCR analysis of *PRMT5* mRNA levels in isolated primary endometrial stromal and epithelial cells from *Prmt5^fl/fl^* and *Amhr2^cre^* *Prmt5^fl/fl^* (*Prmt5^d/d^*) mice. (**B** and **C**) Immunofluorescence (**B**) and Western blot analysis (**C**) of PRMT5 protein in uteri from *Prmt5^fl/fl^* and *Prmt5^d/d^* mice. (**D**) Litter size in *Prmt5^fl/fl^* and *Prmt5^d/d^* mice. Gross morphology detection (**E**) and statistics (**F**) of uterine implantation sites on D5, D6, and D8 in *Prmt5^fl/fl^* and *Prmt5^d/d^* mice. (**G**) Immunofluorescence analysis of COX2 for embryo attachment reaction on D5 and decidual marker DTPRP for decidualization on D6 and D8. (**H**) Gross uterine morphology and weight ratio of stimulated to unstimulated sides in artificial decidualization model in *Prmt5^fl/fl^* and *Prmt5^d/d^* mice. (**I**–**K**) GSK591 or vehicle was administered to normal pregnant mice from D5 to D7, and uteri were collected on D8 for analysis: schematic of the treatment protocol (**I**), representative uterine images and quantification of implantation sites (**J**), and immunofluorescence analysis of the DTPRP in implantation sites (**K**). (**L**) Gross uterine morphology and weight ratio of stimulated to unstimulated sides in artificial decidualization model of GSK591-administered mice. Data represent mean ± SEM. *P < 0.05; ***P < 0.001; ****P < 0.0001; Student’s *t* test. White scale bars: 100 μm (**B**, **G**, and **K**). LE, luminal epithelium; GE, glandular epithelium; S, stroma; Em, embryo.

Consistent with human patterns, PRMT5 was present in both epithelial and stromal compartments of the pregnant mouse uterus, with higher stromal expression at gestation day 4 (D4) compared with D1, started to rise in predecidua at D5, and was robustly upregulated in decidual cells at D6 to D8, implying its potential importance in murine endometrial decidualization (Supplemental Figure 3A). To test the in vivo functions of PRMT5 on endometrial decidualization, we generated 3 transgenic mouse lines. *Pgr*-Cre mice have been widely used to conditionally target genes in both uterine epithelium and stroma (45, 46). But we found *Pgr^Cre/+^* *Prmt5^fl/fl^* mice exhibited developmental defects, which caused severe uterine dysplasia and a complete absence of glands during adulthood (Supplemental Figure 3B). Since the developmental defects have not been observed in patients with RIF, this mouse model may not be suitable for investigating the association of PRMT5 and RIF. *Ltf*-iCre mainly mediates recombination at uterine epithelium (47). Unfortunately, we observed varying degrees of ovarian dysfunction in *Ltf^iCre/+^* *Prmt5^fl/fl^* mice, thereby complicating their utility to study uterine functions (Supplemental Figure 3C). In contrast, *Amhr2*-Cre-mediated *Prmt5* knockout (*Prmt5^d/d^*) maintained normal uterine and ovarian morphology. qRT-PCR analysis of isolated uterine epithelial and stromal cells, together with immunofluorescence staining of uterine sections, revealed a variable yet pronounced reduction of PRMT5 in the uterine stroma of *Prmt5^d/d^* mice at D3.5, which was accompanied by a concurrent decrease in global SDMA levels (Figure 2, A–C, and Supplemental Figure 3D), similar to the marked reduction observed in patients with RIF (Figure 1, E and J). It has been reported that *Amhr2*-Cre has some penetration issues (48). But considering the essential role of PRMT5 in embryonic development (49), we were not surprised that the mice we checked only exhibited restricted deletion within the stromal compartment. Therefore, we primarily investigated the roles of stromal PRMT5 in regulating endometrial receptivity using this mouse model.

First of all, we found no pups were delivered by *Prmt5^d/d^* female mice (Figure 2D). To determine whether the infertility stemmed from initial implantation failure, we examined uteri at D5, when embryo implantation occurred, by intravenous Chicago Blue injection and found out the number of implantation sites was comparable between *Prmt5^fl/fl^* and *Prmt5^d/d^* mice (Figure 2, E and F). The loss of stromal PRMT5 altered neither the embryo attachment marker COX2 expression (50) nor the epithelial receptivity marker FOXO1 expression (51) on D5, indicating that the initial phase of embryo implantation remains uncompromised (Figure 2G and Supplemental Figure 3E). However, a subsequent decidualization defect was observed on D6, as evidenced by attenuated HAND2 expression (Supplemental Figure 3F). On D6 and D8, *Prmt5^d/d^* mice displayed extensive embryo resorptions (Figure 2, E and F), which could be caused by the severe decidualization defect, as indicated by markedly reduced expression of the murine decidual marker DTPRP (52) (Figure 2G). Since decidualization is tightly regulated by the ovarian hormones progesterone and estrogen (5), and *Amhr2*-Cre is also active in the granulosa cells of mice (53), we checked the ovaries of *Prmt5^d/d^* mice. The number of ovulated oocytes, as well as serum estradiol-17β (E2) and progesterone (P4) levels, were comparable to those of *Prmt5^fl/fl^* mice, thereby ruling out ovarian dysfunction as a confounding factor for decidualization (Supplemental Figure 3, G–I). Additionally, we employed an artificial decidualization (AD) model, which reliably recapitulates endometrial decidualization during natural pregnancy in mice while eliminating confounding interferences from ovarian and embryonic signals (54). The decidualization failure in the *Prmt5^d/d^* mice was validated in AD models, characterized by a blunted decidual weight induction and a complete absence of stromal alkaline phosphatase activity (Figure 2H and Supplemental Figure 3J). Collectively, these results demonstrate that stromal PRMT5 is indispensable for endometrial decidualization leading to successful pregnancy.

GSK591 is a well-characterized PRMT5 inhibitor that efficiently inhibits its methylation activity (55). The genetic heterogeneity of outbred ICR mice makes them suitable for medical study (56). To determine whether methylation activity is critical for endometrial decidualization, we administered GSK591 to ICR mice on D5, D6, and D7 after embryo implantation. This treatment markedly diminished stromal SDMA methylation, which resulted in the suppressed decidualization and elevated embryonic resorption at D8 (Figure 2, I–K, and Supplemental Figure 3K). Since PRMT5 is essential for embryonic and ovarian follicle development (49, 57), intraperitoneal injections of GSK591 may also adversely affect embryos and ovaries. To specifically assess the uterine decidual ability following GSK591 treatment while excluding the confounding factors from ovaries and embryos, artificial decidualization was also conducted in the ovariectomized ICR mice. GSK591 administration all through the hormone treatment period in the artificial decidualization model robustly inhibited the decidual response (Figure 2L and Supplemental Figure 3L). These findings establish that the endometrial decidualization is dependent on the enzymatic activity of PRMT5.

### PRMT5 is indispensable for the decidualization of human EnSCs.

**Figure 3:**
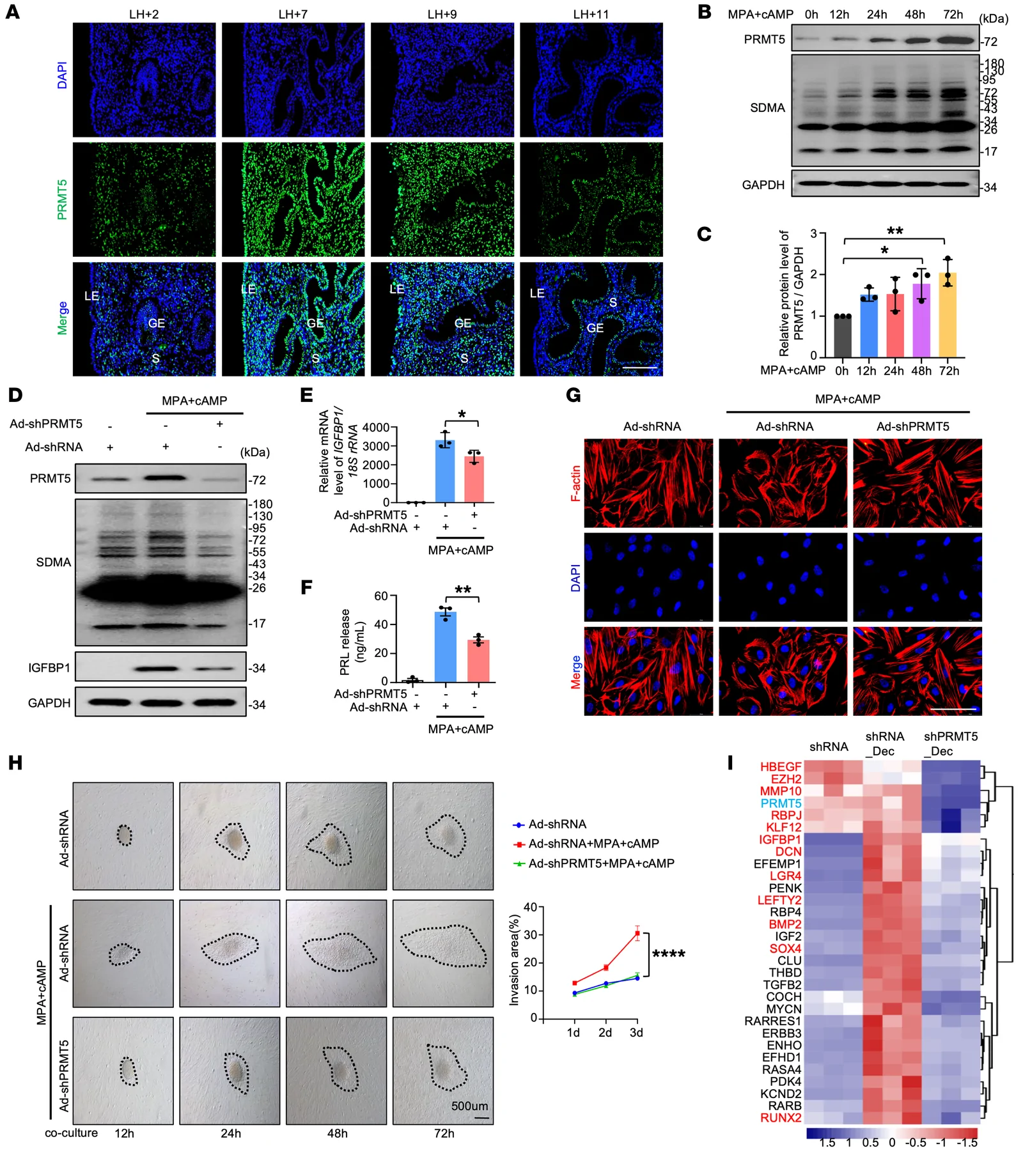
PRMT5 is indispensable for endometrial stromal decidualization and trophoblast invasion in vitro. (**A**) Immunofluorescence analysis of PRMT5 expression patterns in human endometrium across the secretory phase (LH+2 to LH+11). Western blot analysis (**B**) and quantification (**C**) of temporal changes in PRMT5 and SDMA levels in human EnSCs treated with MPA and cAMP for 0, 12, 24, 48, and 72 hours. *n* = 3. (**D**) Western blot analysis of PRMT5, SDMA, and IGFBP1 protein levels in EnSCs transfected with Ad-shRNA or Ad-shPRMT5 followed by decidualization induction (MPA+cAMP). Effects of PRMT5 knockdown on decidualization markers were assessed by RT-qPCR for *IGFBP1* mRNA levels (**E**) and ELISA for PRL secretion (**F**) (*n* = 3). (**G**) Immunofluorescence staining of F-actin (red) showing morphological changes in control and PRMT5-knockdown EnSCs under decidualizing conditions. Nuclei were stained with DAPI (blue). (**H**) Representative images (left) and quantification (right) of trophoblast spheroid invasion into EnSC monolayers. EnSCs were treated with Ad-shRNA or Ad-shPRMT5 and induced with 8-Br-cAMP prior to coculture. The invasion area was measured at 12, 24, 48, and 72 hours. (**I**) Heatmap of RNA-seq data showing the expression profiles of key decidualization regulators and markers in nondecidualized (shRNA), decidualized control (shRNA_Dec), and decidualized PRMT5-knockdown (shPRMT5_Dec) groups. *n* = 3. Data represent mean ± SEM. *P < 0.05; **P < 0.01; ****P < 0.0001. One-way ANOVA with Tukey’s multiple comparisons test was used (**C**, **E**, and **F**). Two-way ANOVA with Bonferroni’s multiple comparisons test was used (**H**). Scale bar: 100 μm.

The in vivo findings demonstrated the important roles of stromal PRMT5 in endometrial decidualization, probably through its methylation activity. Next, we investigated the detailed roles of stromal PRMT5 in decidualization using cultured human EnSCs. First, we characterized its expression pattern across the secretory phase, revealing that PRMT5 protein expression peaked in the midsecretory endometrium at LH+7 (Figure 3A). Reanalysis of a previously published single-cell atlas of human endometrium indicated PRMT5 was mainly expressed in the stromal fibroblasts with peak expressions at LH+7 (Supplemental Figure 4A). The decidualization of human endometrium is induced by progesterone rise and second messenger cAMP production in vivo and can be mimicked in vitro by synthetic progesterone MPA and cAMP (Pc) treatment (10). We found PRMT5 and global SDMA modification were increased after Pc treatment from 0 to 72 hours (Figure 3, B and C). Moreover, treatment with progesterone alone was sufficient to upregulate PRMT5 expression, suggesting that PRMT5 and PRMT5-mediated SDMA were downstream effectors of progesterone signaling during decidualization (Supplemental Figure 4B).

To directly assess the functional requirement of PRMT5, we performed adenoviral-mediated knockdown (Ad-shPRMT5) in primary EnSCs. This intervention substantially attenuated the Pc-induced upregulation of PRMT5 and SDMA (Figure 3D). Critically, PRMT5 deficiency contributed to a functional decidualization defect, evidenced by markedly reduced expressions of canonical markers (*IGFBP1*, *PRL*) and diminished PRL secretion (Figure 3, D–F, and Supplemental Figure 4C). Beyond gene expression, PRMT5 knockdown impaired the morphological transformation of stromal cells from spindle to rounded shape during decidualization (Figure 3G). Since maternal decidualization controls the invasion behavior of the fetal trophoblast (58), it is not surprising that the trophoblast invasion over decidual EnSCs was also compromised by PRMT5 knockdown in EnSCs (Figure 3H). Transcriptomic analysis revealed a broad disruption of the decidualization program by PRMT5 knockdown, characterized by the significant downregulation of key decidual regulators (e.g., *BMP2*, *KLF12*) and decidual markers (e.g., *IGFBP1*, *LEFTY2*), and dysregulated decidualization-related pathways, including extracellular matrix organization, PI3K/AKT signaling, TGF-β signaling, and ECM-receptor interaction (Figure 3I; Supplemental Figure 4, D–H; and Supplemental Table 5). Taken together, these data indicate that PRMT5 is not only upregulated in the EnSCs during the WOI but also functionally indispensable for the endometrial decidualization that facilitates trophoblast invasion.

### PRMT5 methylates Nur77 and regulates its chromatin occupancy during decidualization.

**Figure 4:**
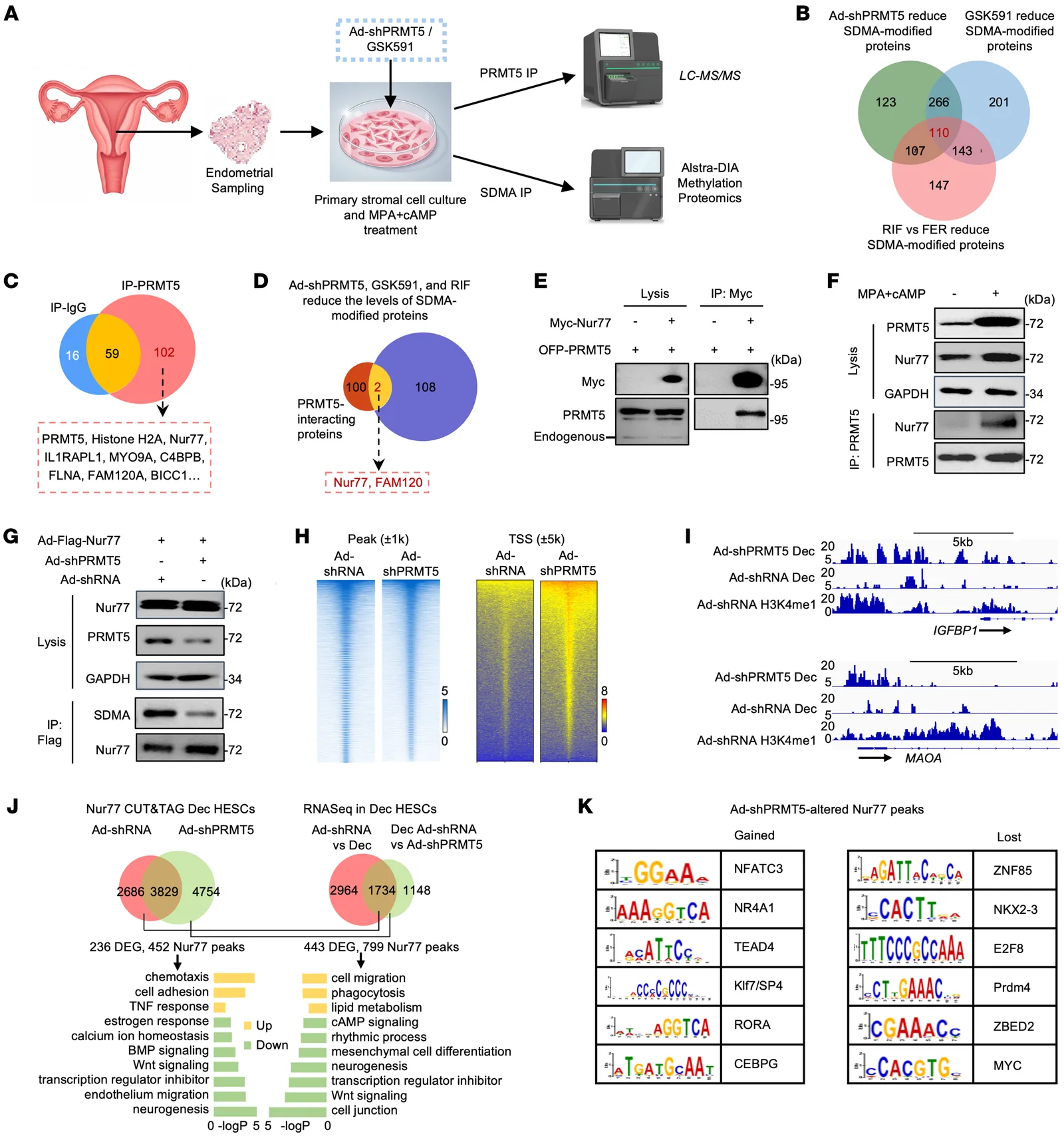
PRMT5 methylates Nur77 and regulates its chromatin occupancy during decidualization. (**A**) Schematic of multiomics strategy to identify PRMT5 downstream effectors in human EnSCs, combining anti-PRMT5 IP-MS and pan-SDMA methylation proteomics. (**B**) Venn diagram showing overlap between SDMA-methylated proteins downregulated upon PRMT5 deficiency (knockdown/inhibition) and those deficient in RIF patient endometria. Intersection of PRMT5-interacting proteins (IP-MS) (**C**) with the 110 targets from **B** identifies Nur77 and FAM120A as core candidates (**D**). *n* = 1 pooled one sample per group. (**E**) Immunoprecipitation validation of overexpressed PRMT5-Nur77 interaction in HEK293T cells. (**F**) Immunoprecipitation analysis of endogenous PRMT5 and Nur77 protein levels and interaction following decidualization induction in EnSCs. (**G**) Western blot analysis of SDMA modification of Nur77 and total Nur77 protein levels upon PRMT5 knockdown. (**H**) Heatmap of Nur77 CUT&Tag peak intensity and signals specifically around the transcription start site (TSS) regions in control and PRMT5-knockdown EnSCs under decidualization. (**I**) Representative genome browser tracks of Nur77 occupancy, monomethylation of histone H3 at lysine 4 (H3K4me1), and input at the *IGFBP1* and *MAOA* (monoamine oxidase A, a mitochondrial enzyme) loci. (**J**) Functional enrichment analysis of differentially expressed genes (DEGs) associated with lost or gained Nur77 peaks upon PRMT5 knockdown. (**K**) Motif analysis at Nur77-bound regions gained or lost after PRMT5 knockdown, respectively. OFP, orange fluorescent protein.

We already reported before that the PRMT5 inhibitor GSK591 could impair the in vitro decidualization of EnSCs, suggesting that PRMT5 regulates decidualization through its methylation activity (42). Therefore, we performed an integrated multiomics screen combining anti-PRMT5 immunoprecipitation–mass spectrometry (IP-MS) and pan-SDMA methylation proteomics in the decidual human EnSCs to identify the critical substrate of PRMT5 during decidualization (Figure 4A). By intersecting SDMA proteins downregulated in human EnSCs upon PRMT5 loss (via knockdown or inhibition) with those deficient in RIF patient endometria, we identified 110 clinically RIF-relevant, PRMT5-dependent SDMA target proteins in endometrial stroma (Figure 4B and Supplemental Table 3). Overlapping with the PRMT5 interactome pinpointed 2 core candidates: Nur77 and FAM120A (Figure 4, C and D, and Supplemental Table 6). We focused on orphan nuclear receptor Nur77, a key decidual regulator (59), whose SDMA modification was unknown. Co-immunoprecipitation confirmed a PRMT5-Nur77 complex in HEK293T cells (Figure 4E). This interaction was more obvious during decidualization in human EnSCs, probably due to elevated PRMT5 and Nur77 expressions (Figure 4F). Furthermore, PRMT5 knockdown specifically blunted SDMA modification on Nur77 (Figure 4G), thereby identifying Nur77 as a direct substrate of PRMT5.

We directly compared the transcriptomic changes in decidual EnSCs after PRMT5 or Nur77 knockdown. While a few genes like *IGFBP1*, *ZNF92*, and *FAM20C* were coregulated, the global overlap was minimal: 94.8% of PRMT5-dependent DEGs and 87.0% of Nur77-dependent DEGs were unique (Supplemental Figure 5A and Supplemental Table 7), arguing against simple functional equivalence. Considering Nur77 is a transcription factor that binds at specific chromatin loci to regulate the transcriptions of its target genes (60), we further examined the alteration of Nur77 chromatin occupancy following PRMT5 knockdown in EnSCs to elucidate the functional consequences of PRMT5 SDMA on Nur77. Strikingly, PRMT5 knockdown failed to alter the global intensity of Nur77 peaks but triggered a robust accumulation of Nur77 peaks at TSS regions (Figure 4H and Supplemental Figure 5, B and C). Specifically, elevated Nur77 occupancy upstream of the TSS coincided with reduced expression of key decidual genes (e.g., *IGFBP1* and *MAOA*) (61), suggesting that aberrantly bound, unmethylated Nur77 may transcriptionally repress these genes (Figure 4I). Conversely, the loss of Nur77 at downstream intergenic regions was also associated with some upregulated genes (e.g., *FUT2* and *PLAC1*) (Supplemental Figure 5D). H3K4me1 typically marks enhancers to facilitate transcription (62). We observed that the gained Nur77 peaks following PRMT5 knockdown showed a stronger overlap with H3K4me1-marked regions compared with the lost peaks, raising the possibility that unmethylated Nur77 may convert these enhancers to silencers (Figure 4I and Supplemental Figure 5, D–F). All these results demonstrate that PRMT5 depletion fundamentally alters the chromatin binding specificity of Nur77, leading to dysregulated decidual gene expression.

To globally assess the regulation of PRMT5/Nur77 axis on gene expression, we performed the integrated analysis of Nur77 CUT&Tag and PRMT5-knockdown RNA-seq data in decidual EnSCs. In total, 236 DEGs, which are coregulated by both PRMT5 knockdown and Pc treatment, associated with lost Nur77 peaks, and 443 DEGs associated with gained peaks (Figure 4J and Supplemental Table 8). Both gene sets are involved in WNT signaling, cell migration, and adhesion pathways essential for decidualization. However, coregulated DEGs associated with lost peaks were enriched in TNF and estrogen responses, which has been reported to regulate decidualization (63, 64), whereas those associated with gained peaks were enriched in phagocytosis and lipid metabolism (Figure 4J). Notably, peaks gained, but not lost, upon PRMT5 knockdown were highly enriched for the canonical Nur77 binding motif, and distinct cofactor partnerships were predicted for the lost peaks. This indicates that PRMT5 knockdown enhanced Nur77’s direct chromatin binding while altering its coordinated protein interactions at chromatin (Figure 4K). Collectively, these data demonstrate that PRMT5-mediated SDMA modification acts as a critical fidelity checkpoint, ensuring Nur77 engages with appropriate chaperones and binds at precise chromatin sites.

### PRMT5-mediated SDMA of Nur77 at R346 is indispensable for decidualization.

**Figure 5:**
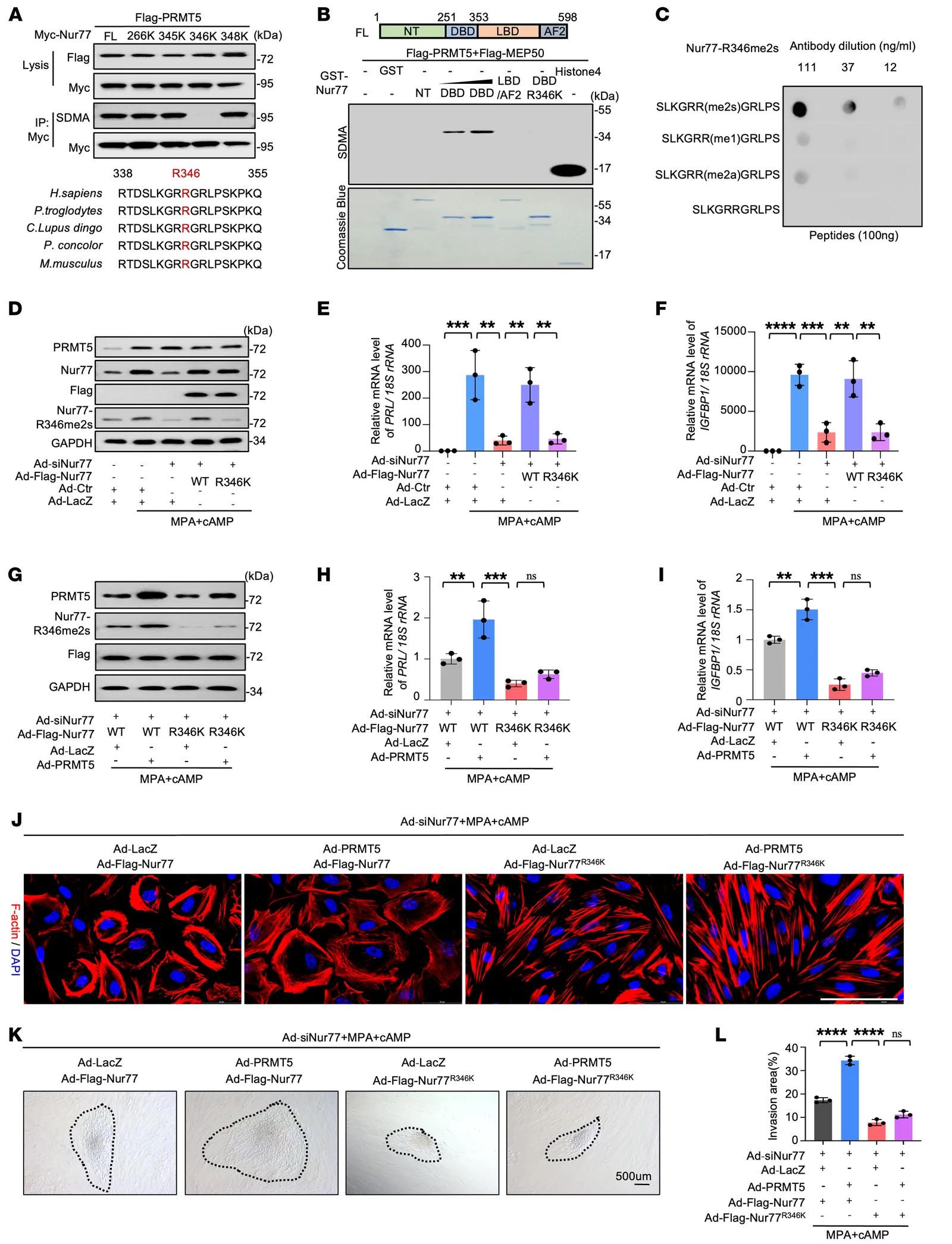
PRMT5-mediated SDMA of Nur77 at R346 is required for decidualization. (**A**) Identification of Nur77 arginine methylation sites. Top: Western blot analysis of SDMA-modified Nur77 in HEK293T cells; bottom: Evolutionary conservation of Nur77 R346 across vertebrate species. (**B**) In vitro methylation analysis of PRMT5 on Nur77 wild-type and various domain mutants. (**C**) Dot blot validation experiment of Nur77-R346me2s–specific antibody, using different antibody concentrations (111, 37, 12 ng/mL) to detect specific binding to various methylated peptides. (**D**) Western blot detection of intracellular Nur77 methylation status following overexpression of wild-type Nur77 and R346K mutant. (**E** and **F**) RT-qPCR analysis of decidualization markers *PRL* (**E**) and *IGFBP1* (**F**) mRNA expression levels in Nur77 knockdown and wild-type or R346K mutant rescue experiments (*n* = 3). (**G**–**L**) Effects of PRMT5 overexpression on decidualization in Nur77-knockdown EnSCs rescued with wild-type or R346K mutant Nur77: Western blot analysis of PRMT5, Nur77, and Nur77-R346me2s (**G**); RT-qPCR analysis of *PRL* (**H**) and *IGFBP1* (**I**) mRNA levels; immunofluorescence analysis of F-actin cytoskeletal remodeling (**J**); trophoblast spheroid invasion assay (**K** and **L**). Data represent mean ± SEM. **P < 0.01; ***P < 0.001; ****P < 0.0001; 1-way ANOVA with Tukey’s multiple comparisons test in (**E**, **F**, **H**, **I**, and **L**). Scale bar: 100 μm. NT, N-terminal domain; DBD, DNA-binding domain; LBD, ligand-binding domain.

Next, we investigated the SDMA modification sites of Nur77 by PRMT5 using 2 methylation prediction tools, PRmePred and GPS-MSP, thereby identifying 4 high-scoring arginine residues, R266, R345, R346, and R348, within the Nur77 DNA-binding domain (DBD). Single point mutation of each residue indicated that only the R346K substitution effectively abolished the PRMT5-activated SDMA signal, an evolutionarily conserved site (Figure 5A). Consistently, in vitro methylation assays confirmed that PRMT5 methylates the DBD of Nur77 specifically at residue R346 (Figure 5B). Because commercial antibodies against methylated Nur77 were not available, we generated a polyclonal antibody exhibiting high affinity to symmetric dimethyl R346 (R346-me2s), with minimal crossreactivity toward the unmodified, monomethyl, or asymmetric forms (Figure 5C). The R346K mutation attenuated Nur77-mediated decidualization of EnSCs (Figure 5, D–F). Importantly, after knockdown of endogenous Nur77, PRMT5 overexpression markedly potentiated the SDMA modification on exogenous wild-type Nur77 and synergistically amplified decidual marker induction, cytoskeletal remodeling, and trophoblast invasion in EnSCs, whereas it had negligible effects on exogenous non-SDMA mutant Nur77^R346K^ (Figure 5, G–L). Collectively, these findings support that PRMT5-mediated SDMA at R346 of Nur77 drives the decidualization program.

### A Nur77-derived peptide with R346K mutant (Pep-Nur77^R346K^) promotes decidualization.

**Figure 6:**
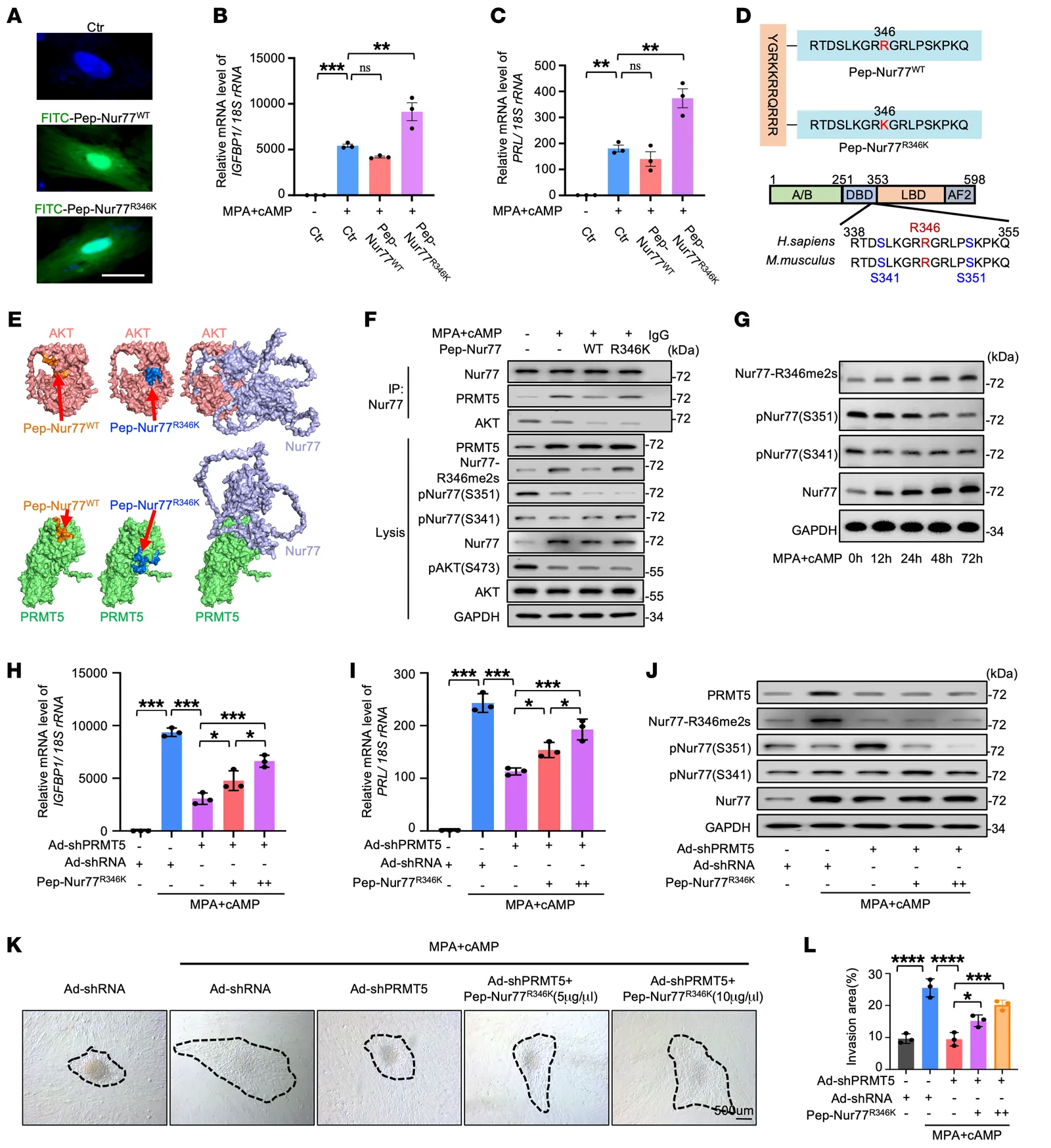
A Nur77-derived peptide with R346K mutant (Pep-Nur77^R346K^) promotes EnSC decidualization. (**A**) Representative fluorescence images showing the intracellular uptake of FITC-labeled wild-type (FITC-Pep-Nur77^WT^) and mutant (FITC-Pep-Nur77^R346K^) peptides in human EnSCs. (**B** and **C**) RT-qPCR analysis of decidualization markers *IGFBP1* (**B**) and *PRL* (**C**) mRNA expression levels in EnSCs treated with control vehicle, Pep-Nur77^WT^, or Pep-Nur77^R346K^ under decidualization conditions (MPA+cAMP). (**D**) Top: Schematic representation of the amino acid sequences of cell-permeable peptides containing the TAT transduction domain. Bottom: Domain structure of Nur77 highlighting the conserved R346 site and phosphorylation sites S341/S351. (**E**) Molecular docking simulation showing the interaction interfaces of AKT and PRMT5 with Pep-Nur77^WT^ and Pep-Nur77^R346K^. (**F**) Co-immunoprecipitation analysis of endogenous Nur77 interactions with PRMT5 and AKT following treatment with Nur77-derived peptides. (**G**) Western blot analysis of temporal changes in Nur77 methylation (R346me2s) and phosphorylation (S341, S351) during the decidualization process (0–72 hours). (**H**–**L**) Functional rescue of PRMT5-depleted EnSCs with Pep-Nur77^R346K^: RT-qPCR analysis of *IGFBP1* (**H**) and *PRL* (**I**) mRNA levels; Western blot analysis of PRMT5, Nur77-R346me2s, pNur77-S341, and pNur77-S351 status (**J**). (**K** and **L**) Trophoblast spheroid invasion assay with representative images (**K**) and quantification (**L**). Data represent mean ± SEM. *P < 0.05; **P < 0.01; ***P < 0.001; ****P < 0.0001; 1-way ANOVA with Tukey’s multiple comparisons test (**B**, **C**, **H**, **I**, and **L**). White scale bar: 50 μm.

Given the critical role of the PRMT5/Nur77 methylation axis in driving decidualization, we explored whether modulating this axis could have therapeutic relevance. We designed 2 cell-permeable peptides derived from Nur77: a wild-type peptide (Pep-Nur77^WT^) and a methylation-deficient mutant (Pep-Nur77^R346K^). We found both peptides were efficiently delivered into the cytoplasm and nucleus of decidual EnSCs (Figure 6A). Surprisingly, only Pep-Nur77^R346K^ strongly enhanced decidual marker expression (*IGFBP1*, *PRL*), while Pep-Nur77^WT^ showed a mild suppressive effect in decidual EnSCs (Figure 6, B and C). Further analysis of the R346 flanking sequence also identified 2 serine residues, S341 and S351, that are established substrates of AKT-mediated phosphorylation, and S351 is known to regulate Nur77 activity (65) (Figure 6D). Molecular docking demonstrated that Pep-Nur77^WT^ bound strongly to the triose-phosphate isomerase barrel region of PRMT5 and AKT LBD, thus competing with the same binding sites of Nur77 (Figure 6E and Supplemental Figure 6, A and B). Pep-Nur77^R346K^, however, retained binding to AKT at the canonical Nur77-binding site, but bound to PRMT5 at a non-Nur77-binding region, implying that it might compete with the AKT-mediated phosphorylation of Nur77 without interfering with the PRMT5-mediated methylation (Figure 6E and Supplemental Figure 6, C and D).

Based on these structural insights, we hypothesized that the peptides function as molecular decoys that selectively sequester upstream enzymes. Co-immunoprecipitation confirmed that Pep-Nur77^WT^ attenuated endogenous Nur77 interactions with both PRMT5 and AKT, reducing both R346 methylation (R346me2s) and S351 phosphorylation (Figure 6F). In contrast, Pep-Nur77^R346K^ specifically disrupted the Nur77-AKT interaction without affecting PRMT5 binding, thereby suppressing inhibitory S351 phosphorylation while preserving R346 methylation of Nur77 (Figure 6F). Through the comparative analysis of these 2 peptides, we found out that dominant R346 methylation of Nur77, relative to its S351 phosphorylation, may play important roles in decidualization. Indeed, during in vitro decidualization, a progressive increase of Nur77-R346 SDMA coincided with the decreased Nur77 phosphorylation, particularly at Ser351 (Figure 6G). Thus, the orchestrated regulation of Nur77 methylation and phosphorylation represents a layer of posttranslational regulation mode of progesterone-driven decidualization but also emerges as a potential intervention point.

We then asked whether Pep-Nur77^R346K^ could rescue defects caused by PRMT5 loss. We found that Pep-Nur77^R346K^ indeed restored decidualization in PRMT5-knockdown cells and reversed the hyperphosphorylation at S351 induced by PRMT5 silencing, while leaving the methylation state at R346 undisturbed, to restore the dominance of R346 methylation of Nur77 (Figure 6, H–J). Eventually, trophoblast invasion defects over PRMT5-knockdown EnSCs were also ameliorated by Pep-Nur77^R346K^ (Figure 6, K and L).

### Pep-Nur77^R346K^ ameliorates decidualization defects and embryo implantation failure in preclinical mouse models.

**Figure 7:**
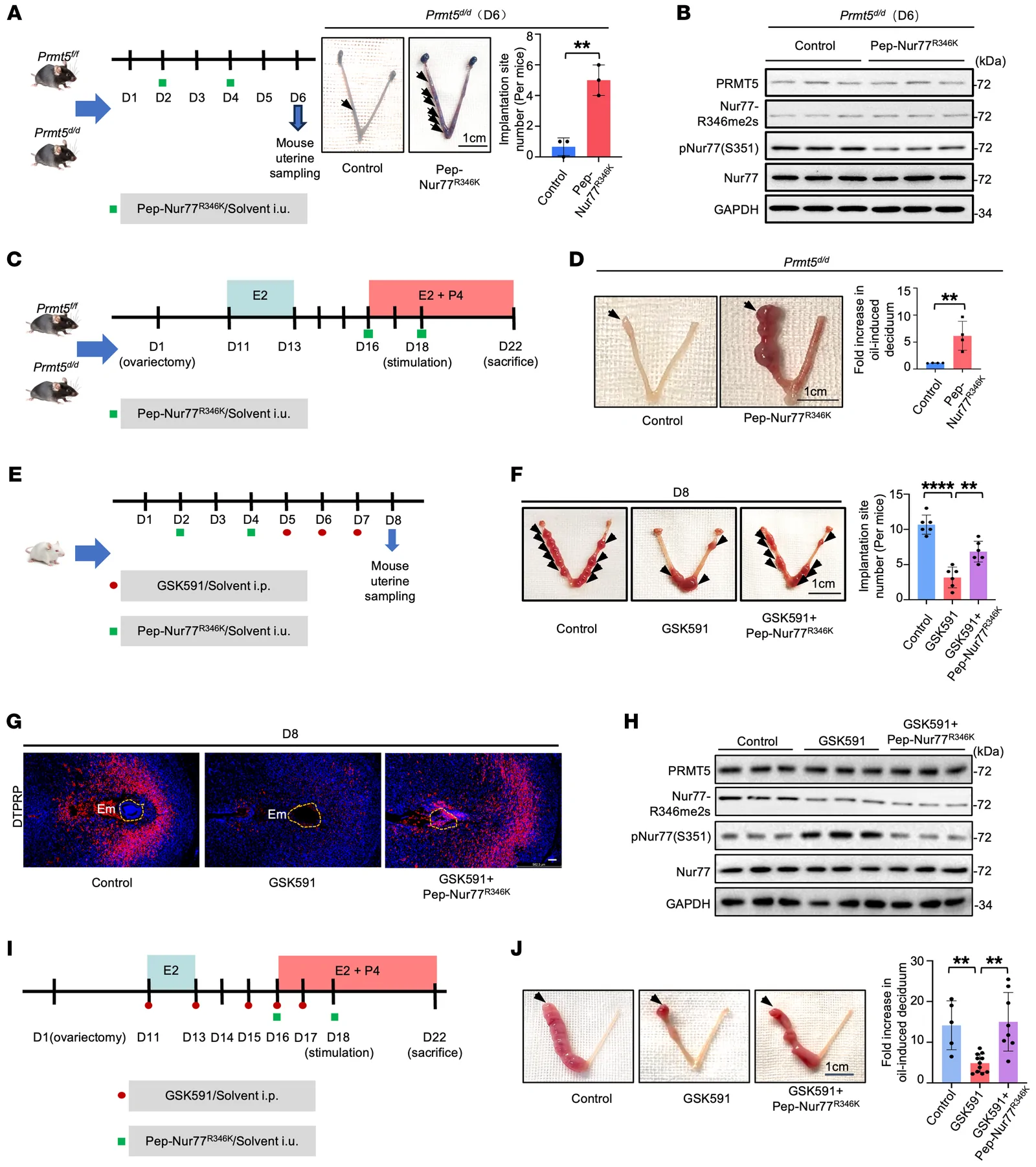
Pep-Nur77^R346K^ ameliorates decidualization defects and embryo implantation failure. (**A**) Effects of Pep-Nur77^R346K^ intrauterine perfusion on implantation site numbers (*n* = 3) on D6 in *Prmt5^d/d^* mice. (**B**) Western blot analysis of PRMT5, Nur77, Nur77-R346me2s, and pNur77 (S351) levels in endometrial tissues of *Prmt5^d/d^* mice treated with Pep-Nur77^R346K^. (**C** and **D**) Effects of Pep-Nur77^R346K^ on artificial decidualization in *Prmt5^d/d^* mice: schematic of the experimental schedule (**C**); gross uterine morphology and weight ratio analysis of decidualized (oil-stimulated) to control sides (**D**). (**E**–**H**) Effects of Pep-Nur77^R346K^ on GSK591-induced implantation failure: schematic of the experimental design (**E**); representative uterine images and quantification of implantation sites on D8 (**F**); immunofluorescence analysis of the decidual marker DTPRP (**G**); Western blot analysis of uterine PRMT5 and Nur77 modification status (**H**). (**I** and **J**) Effects of Pep-Nur77^R346K^ on artificial decidualization in GSK591-treated mice, showing gross morphology and decidual weight ratio. Data represent mean ± SEM. **P < 0.01; ****P < 0.0001; Student’s *t* test (**A** and **D**); 1-way ANOVA with Tukey’s multiple comparisons test (**F** and **J**). Scale bar: 100 μm.

Next, we assessed the therapeutic potential of functional peptide Pep-Nur77^R346K^ in multiple preclinical models. As we described above, *Prmt5^d/d^* mice developed substantial embryonic resorption at D6 due to decidualization defects (Figure 2). Further analysis indicated *Prmt5^d/d^* mice exhibited reduced Nur77 methylation (R346me2s) and heightened Nur77 phosphorylation (S351), recapitulating the posttranslational signature observed in PRMT5-knockdown EnSCs, positioning this genetic model as a physiologically relevant platform for therapeutic validation (Supplemental Figure 7A). FITC-labeled Pep-Nur77^R346K^ was efficiently delivered to mouse uterine cells including stromal cells through intrauterine perfusion (Supplemental Figure 7B). Two administrations of Pep-Nur77^R346K^ before embryo implantation effectively restored the implantation sites and reversed the aberrant elevation of inhibitory phosphorylation (S351) at D6 of *Prmt5^d/d^* mice (Figure 7, A and B). The direct rescue effect of Pep-Nur77^R346K^ on decidualization was further confirmed using an AD model of *Prmt5^d/d^* mice (Figure 7, C and D).

Interestingly, prophylactic treatment with Pep-Nur77^R346K^ before implantation effectively rescued these postimplantation PRMT5 inhibitor GSK591-induced decidualization defects, restoring embryo implantation in ICR mice, probably through the downregulation of S351 phosphorylation of Nur77 (Figure 7, E–H). Besides, in the AD models, we treated the ICR mice with GSK591 all through the hormone treatment period, and 2 administrations of Pep-Nur77^R346K^ at the estrogen and progesterone treatment period before decidual stimulation could completely reverse the impaired decidual responses (Figure 7, I and J). Together, these findings suggest that targeting the Nur77 R346/S351 modification balance with Pep-Nur77^R346K^ may restore decidualization and improve implantation outcomes in preclinical models, warranting further evaluation.

### Reduced endometrial PRMT5/Nur77 methylation is associated with RIF and pregnancy outcome.

**Figure 8:**
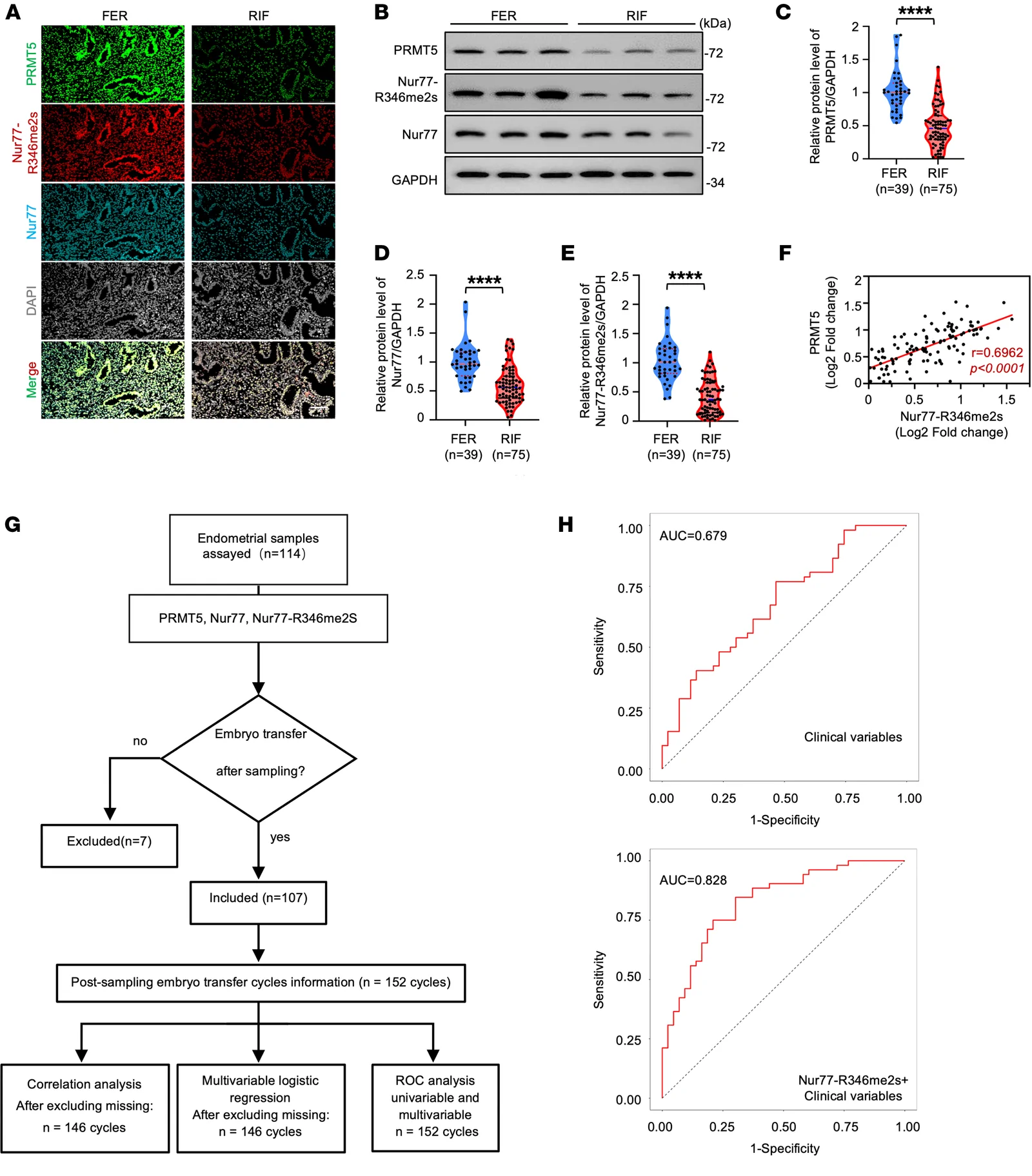
Reduced endometrial PRMT5 and Nur77-R346me2s are associated with RIF and pregnancy outcome. (**A**) Representative immunofluorescence images of PRMT5 (green), Nur77-R346me2s (red), and total Nur77 (cyan) in endometrial sections from FER and RIF patients. Nuclei were stained with DAPI (gray). (**B**) Representative Western blot image of PRMT5 and Nur77 methylation modification levels in endometria from FER and RIF patients. (**C**–**E**) Semiquantitative analysis of protein expression of (**C**) PRMT5, (**D**) total Nur77, and (**E**) Nur77-R346me2s in control (*n* = 39) and RIF (*n* = 75) endometrial tissues. (**F**) Correlation analysis between PRMT5 protein levels and Nur77-R346me2s intensity in human endometrial tissues. (**G**) Flowchart illustrating the inclusion and exclusion criteria for the clinical study participation and analysis. (**H**) ROC curve analysis of clinical variables alone and clinical variables plus Nur77-R346me2s. Data represent mean ± SEM. ****P < 0.0001. Student’s *t* test (**C**–**E**). Scale bar: 100 μm.

Our findings already indicated that PRMT5 deficiency, particularly within stroma, may contribute to endometrial dysfunction in patients with RIF. Immunostaining analysis revealed a pronounced global reduction in methylation in RIF endometrial samples, characterized by markedly attenuated nuclear signals of both PRMT5 and Nur77-R346me2s, along with decreased Nur77 protein levels, within both stromal and epithelial compartments in a subset of RIF patients with ≥5 failed implantations after embryo transfer cycles (Figure 8A). To elucidate the clinical relevance of the PRMT5/Nur77 methylation axis, we expanded our investigation to a larger retrospective cohort (*n* = 114 participants: 39 control and 75 RIF with ≥3 failed implantation after embryo transfer cycles) using semiquantitative Western blot protein analysis. Strikingly, we observed a concurrent reduction in PRMT5, Nur77, and specifically Nur77-R346me2s levels in RIF endometrial tissues (Figure 8, B–E). Moreover, correlation analysis demonstrated a strong positive association between PRMT5 protein levels and the intensity of Nur77-R346me2s modification (Figure 8F). Among these, 107 patients underwent subsequent embryo transfer (152 cycles) (Figure 8G). Spearman’s correlation analysis of 13 variables revealed significant positive correlations among PRMT5, Nur77, and Nur77-R346me2s, all of which were positively associated with clinical pregnancy outcomes (Supplemental Figure 8A and Supplemental Table 9). Logistic regression analysis demonstrated that Nur77-R346me2s levels were significantly associated with clinical pregnancy, with patients in the highest tertile exhibiting a more than 6-fold increased likelihood of pregnancy compared with the lowest tertile (OR: 6.46, 95% CI: 2.52–16.56) (Supplemental Figure 8, B and C). Receiver operating characteristic (ROC) analysis demonstrated that a clinical model incorporating parameters such as age, ovarian reserve, and endometrial thickness predicted pregnancy outcomes with an AUC of 0.679 (Figure 8H). Notably, the addition of endometrial Nur77-R346me2s alone improved predictive performance, yielding AUC of 0.828; further addition of Nur77 and/or PRMT5 did not provide substantial improvement (Figure 8H and Supplemental Figures 9 and 10). All these results demonstrated the endometrial Nur77-R346me2s levels displayed robust predictive capability for pregnancy outcomes.

**Figure 9:**
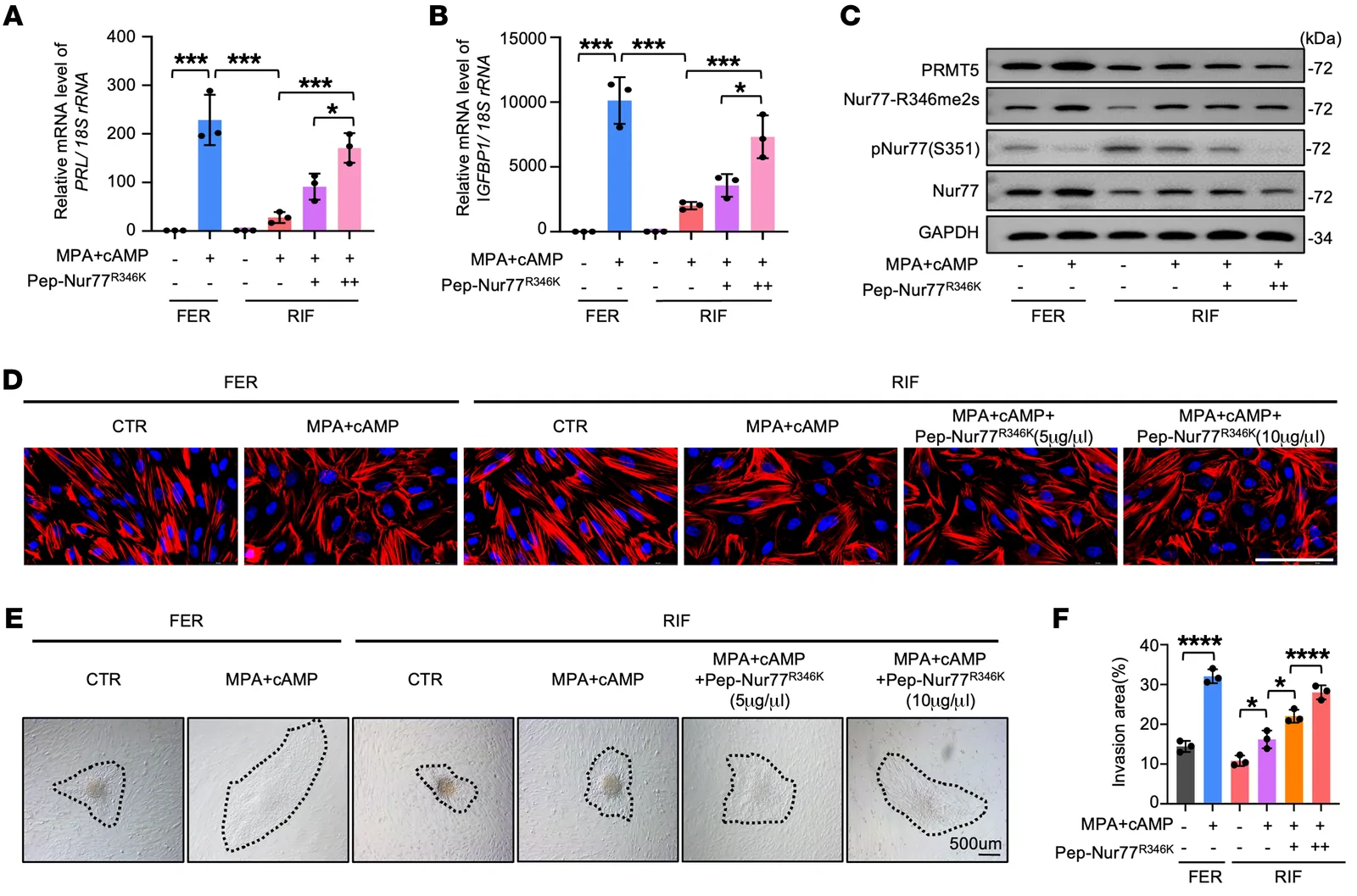
Pep-Nur77^R346K^ restores decidualization-associated responses in RIF-derived EnSCs. (**A** and **B**) Effects of Pep-Nur77^R346K^ treatment on decidualization markers *PRL* (**A**) and *IGFBP1* (**B**) mRNA expression in EnSCs derived from FER and RIF patients. (**C**) Western blot analysis of PRMT5, Nur77 methylation, and phosphorylation modification levels in FER- and RIF-derived EnSCs following Pep-Nur77^R346K^ treatment. (**D**) Immunofluorescence analysis of F-actin cytoskeletal remodeling and (**E** and **F**) trophoblast invasion assay (representative images left, quantification right) in RIF-derived EnSCs treated with or without Pep-Nur77^R346K^ following decidualization induction. Data represent mean ± SEM. *P < 0.05; ***P < 0.001; ****P < 0.0001. One-way ANOVA with Tukey’s multiple comparisons test (**A**, **B**, and **F**). White scale bar: 100 μm (**D**); black scale bar: 500 μm (**E**).

Beyond its predictive value, we asked whether the PRMT5/Nur77 methylation axis could be therapeutically targeted. We isolated primary EnSCs from 5 FER with normal Nur77-R346me2s expressions and from 5 patients with RIF with relatively low Nur77-R346me2s levels, then cultured these cells in vitro. Pep-Nur77^R346K^ improved decidualization responses in RIF-derived EnSCs in a dose-dependent manner (Figure 9, A and B, and Supplemental Figure 11). Mechanistically, the peptide did not restore PRMT5 expression but selectively suppressed pathological pS351 levels without perturbing R346 methylation (Figure 9C). Consequently, it restored cytoskeletal remodeling during decidualization (Figure 9D) and enhanced trophoblast invasion over decidual cells (Figure 9, E and F). Collectively, these findings identify the loss of PRMT5-mediated R346 methylation as a molecular hallmark of RIF and highlight the therapeutic promise of Pep-Nur77^R346K^ for restoring endometrial receptivity.

## Discussion

Here, we revealed that progesterone downstream effector PRMT5-mediated SDMA modifications play a critical role in endometrial decidualization and female fertility. As a type II protein arginine methyltransferase that catalyzes histone methylation, PRMT5 catalyzes SDMA of H2AR3, H3R8, and H4R3, thereby regulating chromatin structure and gene accessibility (66, 67). Recent evidence from animal models has established that histone-modifying enzymes such as EZH2, CFP1, and KMT2D control epithelial or stromal decidualization to regulate endometrial receptivity to embryo implantation (68–70). Accumulating evidence indicates that arginine methylation extends beyond histone substrates to encompass nonhistone proteins, including transcription factors such as p53 (71), androgen receptor (72), and NF-κB p65 subunit (73), which collectively facilitate precise gene expression control. Consistent with this paradigm, our IP-MS analysis identified transcription factor Nur77 as the dominant PRMT5 substrate in EnSCs. Overall, our findings regarding PRMT5-mediated protein arginine methylation add an additional regulatory dimension to endometrial receptivity establishment, distinct from traditional histone-centric epigenetic regulatory models.

We observed a prominent transition from phosphorylation to methylation of Nur77 that is essential for endometrial decidualization. Although several phosphorylation sites of Nur77 have already been identified and shown to regulate its activity (14, 65, 74), the role of Nur77 methylation in decidualization remains poorly defined, particularly its functional interplay with phosphorylation. An interesting finding from our study is that the predomination of methylation over phosphorylation in Nur77 does not alter its protein stability but instead redirects its chromatin occupancy to modulate the transcription of its target genes. The battles between methylation and phosphorylation have also been reported in several cancer cell lines involving multiple PRMT family proteins (75–78). The observed antagonism between methylation and phosphorylation may arise from steric hindrance between the adjacent residues, R346 and S351, a possibility that warrants further investigation. But what is the biological meaning of this competition? There are some clues. We have identified PRMT5 as a downstream effector of progesterone signaling. Concurrently, a positive correlation between AKT kinase activity and estrogen signaling has been well established (78, 79). We propose the transition from phosphorylation to methylation reflects the shift toward progesterone dominance over estrogen during pregnancy. Consequently, our therapeutic strategy may also inform on other diseases characterized by progesterone resistance and estrogen hyperactivity, such as endometriosis, endometrial cancers, and breast cancer.

This study primarily investigated the regulatory roles of stromal PRMT5/Nur77 methylation axis on endometrial decidualization, but we also found PRMT5 downregulation at endometrial epithelium of patients with RIF. The loss of PRMT5 in the pulmonary or intestinal epithelium disrupted epithelial populations through diminished histone SDMA or impaired EGFR/AKT/β-catenin pathways (80, 81). A recent study published by us indicated that epithelial PRMT5 deficiency impairs HOXA10 R337 methylation, altering the transcriptional regulation of adhesion molecules, ultimately impairing embryo implantation (41). All these findings suggest that epithelial PRMT5 reduction may perturb the epithelial differentiation and functions through epigenetic or nonepigenetic mechanisms, contributing to a nonreceptive endometrium in patients with RIF. Along with the PRMT5 deficiency, we further observed a reduction in both total Nur77 and its SDMA modification at the endometrial epithelium of patients with RIF. Previously, we already found epithelial Nur77 could promote embryo attachment in the uterus through elevation of β-integrin expression (14). Therefore, the epithelial PRMT5/Nur77 axis may also play a key regulatory role in establishing the endometrium receptivity. Actually, i.p. injection of GSK591 effectively suppressed PRMT5 methylation activity at both endometrial epithelium and stroma. The observations that Pep-Nur77^R346K^ rescued embryo implantation in GSK591-treated mice implied a conserved mechanism shared by PRMT5-deficient epithelium and stroma. However, the specific functions of epithelial PRMT5 remain to be elucidated and warrant further investigation using appropriate mouse models or cultured human endometrial epithelium systems.

During in vitro fertilization ART, low progesterone levels have been observed on the day of embryo transfer and associated with diminished pregnancy rate (82, 83). Current progesterone luteal support paradigm does not produce the best results (84, 85). Amid extensive discussion about the administration methods, duration, and dosages of progesterone supplement, we developed Pep-Nur77^R346K^, a Nur77-derived peptide designed to enhance PRMT5-dependent Nur77 SDMA during decidualization. Therapeutic peptides, which are specific chains of amino acids with particular biological effects, show great promise in this area. Peptides such as oxytocin and gonadotropin-releasing hormone are important for regulating reproductive hormones (86). In addition to the benefits of Pep-Nur77^R346K^ on endometrial decidualization, we have previously reported another Nur77-derived peptide promoted uterine epithelial cell integrin expression and improves embryo implantation (14). Future studies should consider the combined application of therapeutic peptides to synergistically improve embryo implantation success rates through multitarget regulation.

In summary, this study establishes that PRMT5-catalyzed arginine methylation of the transcription factor Nur77 at R346 is a critical regulator of endometrial stromal decidualization and embryo implantation. The loss of this methylation axis is widely observed in RIF endometria. By translating this mechanism into a functional peptide therapy, we not only provide theoretical insight into endometrial receptivity but also pave the way for a targeted, molecular-based therapeutic strategy for implantation failure.

## Methods

### Sex as a biological variable.

Our study focused on female fertility. Sex was not considered as a biological variable.

### Human endometrial sampling.

Endometrial biopsy specimens were obtained from female patients receiving treatment at the Centre for Reproductive Medicine, Nanjing Drum Tower Hospital, 2018–2024. The FER group included women who underwent in vitro fertilization-embryo transfer treatment for male infertility factor or tubal obstruction and got pregnant after their first or second embryo transfer. The RIF group consisted of patients who had undergone 3 or more consecutive fresh or frozen embryo transfer cycles, with a cumulative total of at least 4 high-quality embryos or 2 high-quality blastocysts that failed to implant. Exclusion criteria included a known uterine abnormality (such as uterine congenital malformation; untreated uterine septum, adenomyosis, or submucous myoma; endometrial polyps; or intrauterine adhesions); a thin endometrium (<6 mm); endometritis diagnosed by hysteroscopy; endometriosis or adenomyosis diagnosed by transvaginal ultrasonography; known autoimmune diseases; currently taking corticosteroids or confounding immunosuppression medications; or abnormal results on parental karyotyping. LH+0 is defined as the day when 5,000 IU human chorionic gonadotropin was injected in women with the leading follicle reaching 18 mm in diameter, and endometrial biopsies were obtained at LH+2, LH+5, LH+7, LH+9, and LH+11, respectively, from patients who did not undergo embryo transfer following in vitro fertilization treatment. This was either because of failed fertilization or the potential risk of ovarian hyperstimulation syndrome in stimulated cycles. The endometrial biopsies were snap-frozen in liquid nitrogen for RNA or protein extraction, or placed in 10% buffered formalin for paraffin embedding, or collected in DMEM-F12 media for isolation of primary EnSCs. Patient information is summarized in Supplemental Table 1.

### Mice and treatment.

*Prmt5^fl/fl^* mice (strain no. T007047, generated by GemPharmatech, Nanjing, China) were crossed with *Amhr2*-Cre mice (87) [Amhr2^tm3(cre)Bhr^, MGI: 4358357] to generate uterine stroma-specific mutant mice (*Prmt5^d/d^*), crossed with *Pgr*-cre mice (46) [B6.129S(Cg)-Pgr^tm1.1(cre)Shah^/AndJ, JAX 017915] to generate uterine specific knockout mice, and crossed with *Ltf*-iCre mice [Ltf^tm1(icre)Tdku^/J, JAX 026030] (47) to generate uterine epithelium-specific knockout mice. *Amhr2-Cre* mice were provided by Haibin Wang at Xiamen University (Xiamen, China). All these mice were bred in our animal facility for multiple generations. ICR mice at 6 weeks old were purchased from Nanjing Ziyuan Biotechnology (Nanjing, China) and acclimatized in our animal facility for 2 weeks, then subsequently used for PRMT5 inhibitor experiments. The detailed experiments were listed in Supplemental Methods.

### EnSC culture and treatment.

Primary human EnSCs were isolated from the endometrial biopsy of FER women recruited in this study and cultured in vitro for adenovirus transduction, plasmid transfection, hormone stimulation, and inhibitor treatment. The detailed information is in Supplemental Methods.

### Multiomic analysis of human endometrial sample and/or EnSCs.

RNA-seq, CUT&Tag, PTMScan SDMA motif enrichment, and Alstra-DIA methylation proteomics were conducted in the human endometria and EnSCs as described in Supplemental Methods.

### Three-dimensional structure prediction of protein–protein interaction.

To investigate the binding regions and interaction patterns among PRMT5, Nur77, and AKT proteins, we used the professional protein-protein and protein-DNA/RNA docking program HDOCK (88). The structure with the highest docking score was selected as the standard result for subsequent interaction analysis. The docking scores were based on the ITScorePP or ITScorePR iterative scoring functions (89, 90).

### Peptides.

To facilitate peptide crossing into the cells, we included the transactivator of transcription sequence (GRKKRRQRRR) at the C-terminus of all the peptides (86). For intraperitoneal injection in mice and treatment of cultured cells, FITC, along with a 6-aminohexanoic acid linker, was added at the N-terminus. The sequences of the examined peptides from Nur77 are as follows: Pep-Nur77^WT^: NH2-RTDSLKGRRGRLPSKPKQYGRKKRRQRRR-COOH, Pep-Nur77^R346K^: NH2-RTDSLKGRKGRLPSKPKQYGRKKRRQRRR-COOH. All peptides were synthesized (GenScript Biotech) at over 98% purity, as verified using high-performance liquid chromatography and MS.

### Nur77-R346me2s antibody.

Anti–Nur77-R346me2s rabbit polyclonal antibody was generated by ABclonal. Briefly, me2s-modified antigen peptide, SLKGRR (me2s) GRLPS, was conjugated with keyhole limpet hemocyanin, then subjected to a 10-week immunization schedule in rabbits. The harvested immune serum was subjected to the SLKGRR (me2s) GRLPS peptide column for affinity purification, then to the nonmodified peptide (SLKGRRGRLPS) column to subtract antibodies recognizing total protein. Dot blotting was performed to detect the specificity of Nur77-R346me2s antibody to the me2s-, me1-, and me2a-modified peptides and nonmodified peptide. The antibodies and primers used in this study are listed in Supplemental Table 10.

### Statistics.

The experiments were conducted in triplicate (minimum). The statistical analyses were conducted using the Prism version 9 software, developed by GraphPad, or R software. The data are presented as the means ± SEM. The 2-tailed Student’s *t* test was used to compare the average expression values between the 2 treatment groups. A 1-way ANOVA was conducted to compare multiple groups. Two-way ANOVA with the Bonferroni multiple comparisons test was performed to analyze the interaction effects of more than 2 groups. *P* < 0.05 was considered statistically significant. Logistic regression analysis was performed to evaluate the association in an expanded cohort, with ORs and 95% CIs calculated for patients grouped by tertiles. The predictive performance of clinical parameters, with or without the inclusion of endometrial PRMT5, Nur77, or Nur77-R346me2s, was assessed using ROC curve analysis, and the AUC was calculated to evaluate diagnostic accuracy.

### Study approval.

The Institutional Review Boards at Nanjing Drum Tower Hospital approved the human research (2013-081-02). All participants provided written informed consent before the review of medical records and sampling procedure. All animal experiments were approved by the Institutional Animal Care and Use Committee of Nanjing Drum Tower Hospital (SYXK 2019-0059).

### Data availability.

The raw FASTQ files for RNA-seq and CUT&Tag generated in this study have been deposited at the Genome Sequence Archive of the National Genomics Data Center, China National Center for Bioinformation/Beijing Institute of Genomics, Chinese Academy of Sciences, under the following accession numbers: GSA PRJNA1050378 and PRJCA065814. These datasets are publicly accessible at https://ngdc.cncb.ac.cn/ The full lists of PRMT5 IP-MS and proteins with altered SDMA levels in endometrium of RIF patients and EnSCs after PRMT5 knockdown or GSK591 treatment are provided in the supplemental tables. Values for all data points in graphs are reported in the Supporting Data Values file.

## Author contributions

GY, HS, RJ, and RL initiated and supervised the project. ZC, XC, RJ, and JM performed the experiments and collected the data. YL and XZ contributed to the animal models and animal analysis. MW, NK, and XS contributed to the human endometrium and endometrial stromal cell experiments; RJ and ZC wrote the manuscript. GY, HS, RL, and JS reviewed and edited the manuscript.

## Conflict of interest

The authors have declared that no conflict of interest exists.

## Funding support

- National Key Research and Development Program of China 2023YFC2705400 (to GY).
- National Natural Science Foundation of China 82371680 and 31872846 (to GY), 82471703 (to HS), 82271698 (to RJ), 82301899 (to XC), 82502025 (to ZC), 82502024 (to RL).
- Frontier Technologies R&D Program of Jiangsu Province BF2025626 (to HS).

## Supplementary Material

Supplemental data

Unedited blot and gel images

Supplemental table 1

Supplemental table 10

Supplemental table 2

Supplemental table 3

Supplemental table 4

Supplemental table 5

Supplemental table 6

Supplemental table 7

Supplemental table 8

Supplemental table 9

Supporting data values

## Version 1 07/17/2026

In-Press Preview

## Version 2 09/01/2026

Electronic publication
