# Supplementary material for: Targeting the PRMT5/Nur77 methylation axis enhances endometrial decidualization capacity and female fertility in preclinical models
Source: J Clin Invest. 2026 Jul 16;136(17):e178862. doi: 10.1172/JCI178862 (PMC13528935; doi:10.1172/JCI178862)
Supplement: Supplementary file 1 [file jci-136-178862-s191.pdf]

MATERIALS AND METHODS

Animals and treatments

To conduct the early pregnancy study, C57BL/6 male mice were mated with female *Prmt5*$^{f/f}$ and *Prmt5*$^{d/d}$ mice at 8 weeks of age. The observation of a vaginal plug marked the beginning of gestation, referred to as day 1 (D1). To examine implantation, pregnant mice were sacrificed on the morning of D5, D6, and D8 (n = 13 for *Prmt5*$^{f/f}$, n = 13 for *Prmt5*$^{d/d}$). Implantation sites were visualized by an intravenous injection of Chicago blue dye solution, and the number of implantation sites, demarcated by distinct blue bands, was recorded. Mice that failed to recover any embryos were excluded from the statistical analysis. For the peptide rescue assay, pregnant *Prmt5*$^{d/d}$ mice were subjected to intrauterine (i.u.) injections of a mutant peptide (Pep-Nur77^{R346K}) or solvent on D2 and D4, and dissected on D6 to check embryo implantation.

To evaluate the impact of PRMT5 inhibition in vivo, pregnant C57BL/6 mice received intraperitoneal (i.p.) injections of GSK591 (300μL, 0.2mg/10g bodyweight) or vehicle solvent daily from D5 to D7, and uterine tissues were sampled on D8. For the peptide rescue assay, pregnant mice were subjected to intrauterine (i.u.) injections of 10μL mutant peptide (5 μg/μL, Pep-Nur77^{R346K}) or solvent on D2 and D4. Subsequently, these mice received daily i.p. injections of GSK591 or solvent from D5 to D7, and uterine samples were harvested on D8.

To stimulate decidualization in vivo, female mice aged 6–8 weeks with the *Prmt5*$^{f/f}$ and *Amhr2*^{cre}*Prmt5*$^{f/f}$ genotypes were subjected to ovariectomy followed by sequential subcutaneous injection of estrogen (100ng E2, Sigma-Aldrich, St. Louis, MO, USA;

#E2758) and progesterone (2mg P4, Sigma-Aldrich, #P0130), as described previously (*51*) (n = 3 for each group). One of the uterine horns was subjected to artificial decidualization by injecting 20 μl of sesame oil into its lumen, and the other uterine horn was left untreated as a control. GSK591 inhibitor (300μL, 0.2mg/10g bodyweight) was i.p. injected daily during all the hormone treatment period. For peptide rescue experiments in the artificial decidualization model, 10 μL peptide solution (5 μg/μL, experimental group, n = 4) or saline (control group, n = 4) was injected into the uterine cavity through the cervix before oil injection. Specifically, for the Pep-Nur77^{R346K} rescue experiment, ovariectomized mice underwent an E2 priming regimen (100ng, D11–D13). During the subsequent P4 plus E2 (2mg P4+ 10ng E2) maintenance phase, mice received intrauterine injections of Pep-Nur77^{R346K} or solvent on D16 and D18, respectively, and were sacrificed on D22.

Cell culture and *in vitro* decidualization of endometrial stromal cells

According to the previously mentioned protocol, the primary human EnSCs were extracted from the endometrial biopsy of control fertile and RIF patients recruited in this study (91). To induce decidualization, EnSCs were cultured in DMEM/F12 (Corning, Corning, NY, USA) containing 2.5% charcoal/dextran-treated FBS (Hyclone, Logan, UT, USA), 100 IU/ml penicillin, and 100 μg/ml streptomycin. Concentrations of 0.5 mM 8-Br-cAMP (Sigma, #B7880) and 1 μM of medroxyprogesterone acetate (MedChemExpress, #HY-B0469) were applied. The hormonal counselling was denoted as MPA+cAMP. Before inducing decidualization, cells were exposed to pretreatment

with Ad-shPRMT5 (50 MOI), Ad-siNur77 (50 MOI), Ad-Flag-Nur77 (50 MOI), Ad-PRMT5 (50 MOI), or peptides for 48 h.

HEK293T cells (RRID: CVCL_0063) were purchased from ATCC (Manassas, VA, USA), cultured under standard conditions and treated with Ad-Flag-Nur77 (50 MOI), Ad-PRMT5 (50 MOI) for co-immunoprecipitation as previously described (14). Cycloheximide (Millipore, Burlington, MA, USA; #508739) was introduced at a concentration of 50 μg/mL for the specified durations to conduct the protein degradation experiment. All cells of the control groups were treated with the corresponding control adenoviruses or solvent in equal concentrations.

Construction of adenoviruses

As previously stated, we generated the adenovirus that carries PRMT5 (Ad-PRMT5), shPRMT5 (Ad-shPRMT5) (42), Nur77 (Ad-Flag-Nur77), and siNur77 (Ad-siNur77) (14). Ad-LacZ was used as the control virus for Ad-Flag-Nru77 or Ad-PRMT5. Ad-Ctrl and Ad-shRNA were used as the control virus for Ad-siNur77 and Ad-shPRMT5, respectively. The adenoviruses were isolated by CsCl banding and subsequently dialyzed against 20 mmol/L Tris-buffered saline with 10% glycerol after being propagated in HEK293A cells.

Recombinant protein purification

GST-Nur77-NT, GST-Nur77-LBD, GST-Nur77-DBD, and GST-Nur77-DBD^{R346K}

fusion proteins were generated as described previously (14). Briefly, the fusion proteins were produced in BL21 Escherichia coli bacteria using the pGEX4T-1 vector, then a solitary colony was introduced into Luria broth medium for induction. Pierce Glutathione Agarose (Thermo Scientific, Waltham, MA, USA; #16101) was incubated with the lysate supernatant at 4 °C for 3 h, followed by elution. After dialysis in Slide-A-Lyzer Dialysis Cassettes (Thermo Scientific, #66380) for 48 h, the proteins were separated into groups and stored at –80 °C.

Immunohistochemistry (IHC) analysis

The tissues were fixed in 4% paraformaldehyde (PFA) for 24 h before being embedded in paraffin wax. The tissue sections underwent deparaffinization and rehydration before being exposed to antigen retrieval. The sections were exposed to the primary antibody (Table S10) for an extended time at 4 °C. This was followed by the application of IHC staining kits purchased from Zhongshan Golden Bridge. A Leica DM 2000 microscope and LAS Core software (Leica Microsystems Limited, Wetzlar, Germany) were used to take digital images. The protein expression levels in the epithelial cells and stromal cells of the endometrial samples were quantitatively analyzed using the integrated optical density (IOD) of the digital pictures (×400) with the Image-Pro Plus System 6.0.

IF staining

The cells underwent fixation by treatment with a 4% PFA solution for a duration of 20

min. Next, the samples were treated with 0.1% Triton X-100 in phosphate-buffered saline (PBS) to make them permeable for 5 min at 25 °C. To avoid non-specific binding, the cells were treated with a solution of 3% bovine serum albumin (BSA) in PBS and incubated at 37 °C for 1 h. Subsequently, primary antibodies (Table S10) were introduced, and the cells were subjected to overnight incubation at 4 °C. The signal was visualized using fluorescence-conjugated secondary antibodies. Subsequently, the nuclei were subjected to 4’,6-diamidino-2-phenylindole (DAPI) staining for a duration of 5 min, and images were captured using fluorescence confocal microscopy.

Co-immunoprecipitation (Co-IP)

Tissues or cells were subjected to protein extraction using a lysis buffer consisting of 1% NP-40, 150 mmol/L NaCl, 50 mmol/L Tris (pH 8), 100 μmol/L EDTA, and protease inhibitors. The cell lysates were subjected to pre-clearance using protein A/G beads (Abmart, Shanghai, China; #A10001M) at 4 °C for 2 h. Subsequently, 5 μg of the primary antibody or isotype IgG (Table S10) was introduced into the purified cell extracts and left to incubate overnight at 4 °C. Subsequently, protein A/G beads were introduced into the cell extracts and subjected to incubation at a temperature of 4 °C for 4 h. For IP of exogenous, Flag-beads (MilliporeSigma, #F1804) or Myc-beads (MilliporeSigma, # E6654) were introduced to the purified cell extracts and left to incubate overnight at 4 °C. Ultimately, the proteins that were attached to the beads were released by introducing 2× Laemmli sample buffer and subjecting them to a temperature of 95 °C for 5 min. The proteins that were separated and collected were

examined using western blotting.

CUT&Tag assay

Genome-wide chromatin profiling of Nur77 was performed using the Hyperactive Universal CUT&Tag Assay Kit for Illumina Pro (Vazyme Biotech, TD904) and ChIP grade antibody (Table S10), following the manufacturer's instructions with minor modifications. Briefly, approximately 100,000 dissociated cells per sample were washed and immobilized on activated Concanavalin A (ConA)–coated magnetic beads (ConA Beads Pro). The bead-bound cells were incubated with primary antibodies against Nur77 (1:50 dilution; Proteintech, 12235) or a species-matched IgG control overnight at 4°C in antibody buffer containing digitonin. Following a wash step, cells were incubated with appropriate anti-species secondary antibodies for 60 min at room temperature and subsequently treated with Hyperactive pA/G-Transposon Pro (pA/G-Tnp Pro) for 1 h. Tagmentation was activated by incubating the cells at 37°C for 1 h in Trueprep Tagment Buffer L (TTBL) to cleave DNA at antibody-bound sites. DNA extraction was performed using DNA Extract Beads Pro, and the resulting libraries were amplified via PCR. Post-amplification purification was conducted using VAHTS DNA Clean Beads (Vazyme, N411). Library quality and concentration were assessed using an Agilent 2100 Bioanalyzer and Qubit, respectively. Sequencing was carried out on an Illumina NovaSeq 6000 platform with 150 bp paired-end reads. Raw sequencing data were preprocessed with Cutadapt (92) (version 2.9) to remove adapters and low-quality reads. Cleaned reads were aligned to the human reference genome (hg38) using

Bowtie2 Suite (93) Peak calling was performed using MACS2 (94) with a q-value cutoff of 0.001. Transcription start site (TSS) enrichment was quantified using MAnorm (95). Peak-associated genes were annotated using GREAT (96), and motif discovery was conducted with the MEME Suite (97).

Western blot analysis

Proteinase and phosphatase inhibitor cocktails (Roche Life Science, Basel, Switzerland; #11697498001) and 50 mM Tris-HCl [pH 7.6], 150 mM NaCl, and 1.0% NP-40 were contained in a whole-cell lysis buffer (MilliporeSigma, #P5726) to homogenise tissues and cells. The proteins underwent separation using a 10% sodium dodecyl sulfate–polyacrylamide gel electrophoresis (SDS-PAGE) gel and were subsequently transferred onto polyvinylidene difluoride (PVDF) membranes (Millipore, #03010040001). Subsequently, the membranes were subjected to incubation with primary antibodies (Table S10), which were subsequently followed by a secondary antibody conjugated with horseradish peroxidase (HRP). The detection was conducted using an enhanced chemiluminescence (ECL) kit from Millipore-Sigma, with the product code #32106. To determine the comparative prevalence of the target proteins, the level of expression for each protein was adjusted based on that of GAPDH in the corresponding sample. The signal intensities were measured using densitometric analysis with the ImageJ software as described previously (98). Background subtraction was performed on a lane-by-lane basis. Three independent biological replicates were analyzed for the mouse uterine tissues and EnSCs. 39 control and 75 RIF endometrium were included

for the retrospective RIF cohort western analysis.

Silver nitrate staining and liquid chromatography-tandem mass spectrometry (LC-MS/MS)

The proteins obtained from Co-IP were examined using 10% SDS-PAGE and detected using the Fast Silver Stain Kit (Beyotime, #P0017s), following the manufacturer's instructions. Next, LC-MS/MS was performed and analyzed by Hoogen Biotech (Shanghai, China).

PTMScan® SDMA motif enrichment and Alstra-DIA Methylation Proteomics

PTMScan® SDMA motif enrichment kit (35985, Cell Signaling Technology) was applied to enrich the SDMA peptides for following proteomic analysis according to the manufacture's protocols SDT lysis for protein extraction, followed by BCA quantification, was used for protein methylation modification identification. Appropriate amounts of protein underwent trypsin digestion using Filter-aided proteome preparation (FASP) methodology. Briefly, proteins were reduced with DTT, alkylated with IAA, filtered through 10 kDa ultrafiltration units, digested with Lys-C and trypsin, and finally desalted using C18 Cartridges. Resulting peptides were enriched for methylated peptides using CST methylation peptide enrichment kits. The Vanquish Neo Ultra-High-Performance Liquid Chromatography (UHPLC) System (Thermo Scientific) was used for peptide separation, with samples captured using a

Trap Column (PepMap Neo 5 μm C18 300 μm × 5 mm) before separation using a μPAC Neo High Throughput analytical column. The liquid chromatography gradient consisted of mobile phase B (0.1% formic acid in acetonitrile/water, 80% acetonitrile) linearly increasing from 4% to 45% over a 24-min total run time. An Orbitrap Astral mass spectrometer (Thermo Scientific) operating in data-independent acquisition (DIA) mode was used for MS analysis, with electrospray voltage at 2.2 kV, MS1 resolution of 240,000, MS2 resolution of 80,000, precursor scan range 380–980 m/z, HCD fragmentation, and normalized collision energy of 25%. Raw MS data files were searched using the Spectronaut software for identification and quantitative analysis of methylated peptides. The subcellular location of SDMA proteins in control and RIF endometrial samples was retrieved from Uniport (99).

RNA isolation and real-time qPCR

Cells underwent TRIzol treatment (Takara Bio, Kusatsu, Shiga, Japan; #T9108) for the extraction of total RNA, following the manufacturer's instructions. The assessment of RNA purity involved quantifying the optical density at wavelengths of 260 nm and 280 nm, whereas the determination of RNA integrity was accomplished using agarose gel electrophoresis. A total of 1 μg sample of total RNA was used to synthesize the first strand of DNA (cDNA) using the Takara PrimeScript RT reagent kit (Takara Bio, #RR037A). The analysis of gene expression levels was conducted using SYBR Premix Ex Taq kits (Takara Bio, #RR820A) and the corresponding primers (Table S10) using real-time PCR. The $2^{-\Delta\Delta Ct}$ method was used to compute the relative gene expression

levels, with 18S RNA as the internal control.

RNA-seq and data analysis

Endometrial stromal cells were cultured in 60 mm dishes and exposed to Ad-shPRMT5 for 48 h. Subsequently, the cells underwent decidualization for an additional 72 h. The cells were subjected to RNA extraction using TRIzol (Takara Bio, #T9108). RNA-seq was conducted, and the subsequent analysis of the data was performed by BGI Genomics Co., Ltd, located in Shenzhen, China. The clean reads were aligned to the human reference genome using HISAT2. DESeq2 (100) was used to detect differentially expressed genes (DEGs), with a significance threshold of $p < 0.05$ and $|\log_2 \text{foldChange}| > 1$ to determine significant differences. ClusterProfiler (101) was used to conduct GO, KEGG, and Gene Set Enrichment Analysis (GSEA). We analyzed the transcription factor regulatory network using KnockTF (102) (http://www.licpathway.net/KnockTF/index.php).

Enzyme-linked immunosorbent assay (ELISA)

Following 72 h of decidualization, the culture supernatants of human EnSCs were gathered and subsequently subjected to centrifugation to eliminate cellular debris. The PRL levels were quantified using a commercially accessible ELISA kit (R&D Systems, Minneapolis, MN, USA; #DPRL00), following the manufacturer’s instructions. The samples were analyzed twice, and the PRL level was quantified as nanograms per

milliliter of the liquid surrounding the cells.

ALP staining Assay

The frozen sections were warmed up and fixed for 1 min, following the manufacturer's instructions (Solar bio, Beijing, China; #G1480). ALP incubation was dripped to cover the tissues for 1 h, followed by nuclear red solution staining. Photographs were captured using a Leica DM 2000 microscope and the LAS Core software.

Supplemental Figures

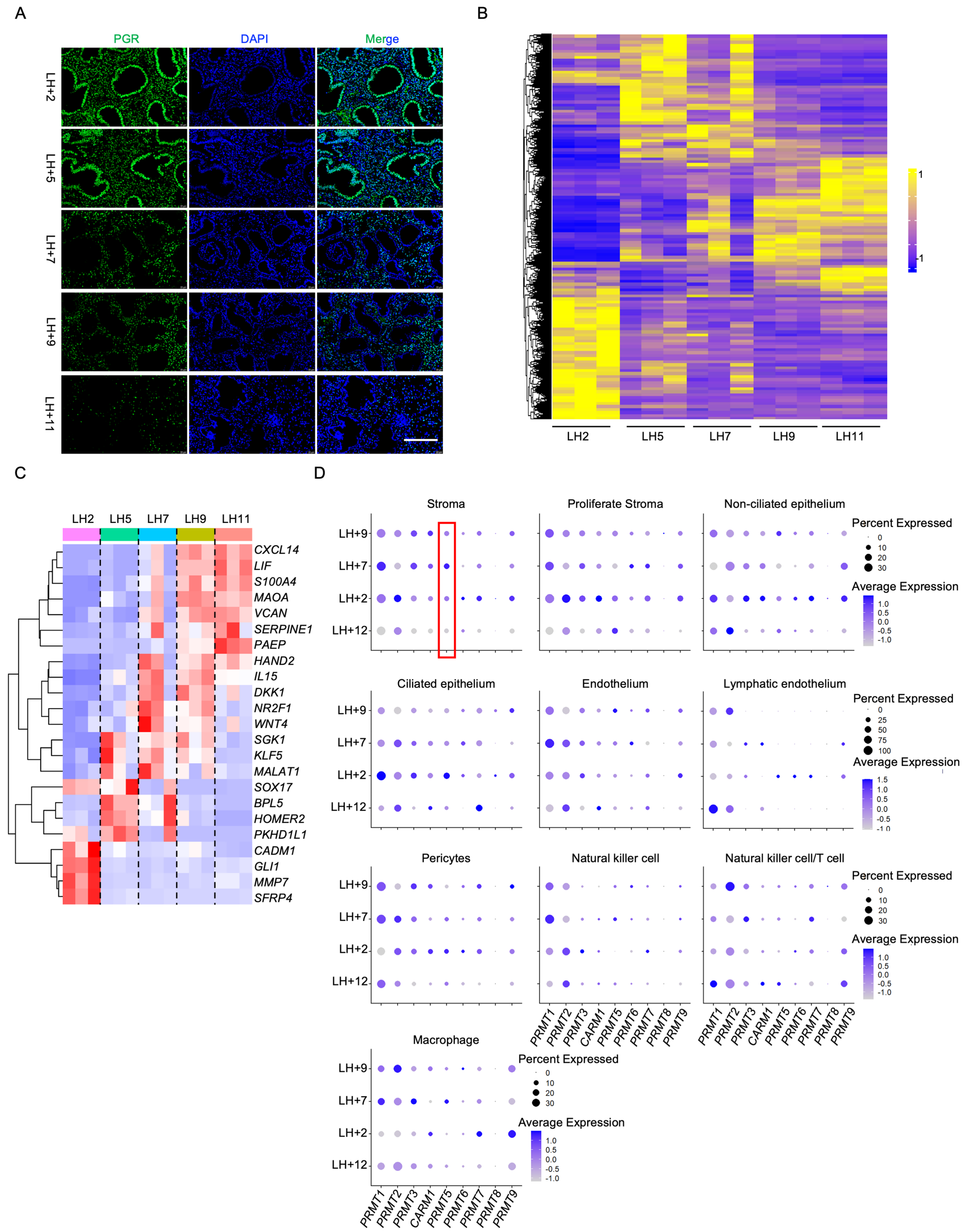

Supplemental Figure 1. Molecular dynamics of human endometrial receptivity and PRMT expression during the LH+ window. (**A**) Representative immunofluorescence images of Progesterone Receptor (PGR, green) in human endometrial tissues collected at varying days post-LH surge (LH+2 to LH+11). Nuclei are stained with DAPI (blue). (**B**) Heatmap visualizing the global gene expression dynamics across different time points of the secretory phase (LH+2 - LH+11). (**C**)

Heatmap showing the expression profiles of specific endometrial receptivity-associated genes (classified into differentiation and proliferation markers) across the secretory phase. (**D**) Single-cell dot plots showing the temporal expression patterns of PRMT family genes across major human endometrial cell types at different days post-LH surge. Scale bar: 100 μm.

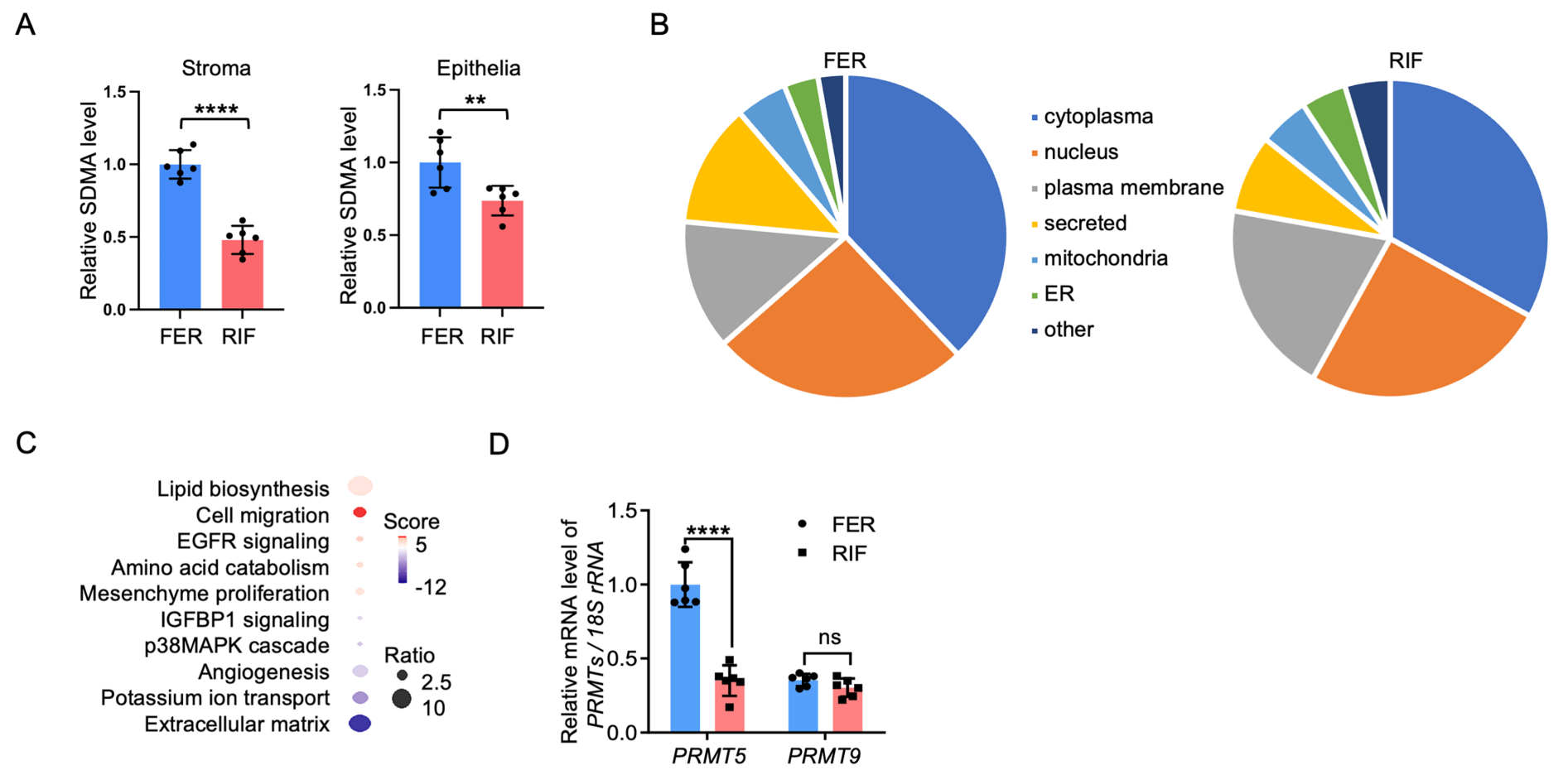

Supplemental Figure 2. Altered SDMA modification profiles and PRMT expression in the endometrium of RIF patients. (**A**) Immunofluorescence analysis of symmetric dimethylarginine (SDMA, green) in FER and RIF patients (n = 6). (**B**) Pie charts displaying the subcellular localization classification of SDMA-modified proteins identified in FER and RIF groups. (**C**) Bubble plot showing the functional enrichment analysis of transcriptionally differentially expressed molecules in endometrium from RIF patients. (**D**) RT-qPCR analysis of *PRMT5* and *PRMT9* mRNA levels in endometrial tissues from FER and RIF patients (n = 6). Data represent mean ± SEM. $^{**}P < 0.01$, $^{****}P < 0.0001$; ns, no significance, Student's t-test.

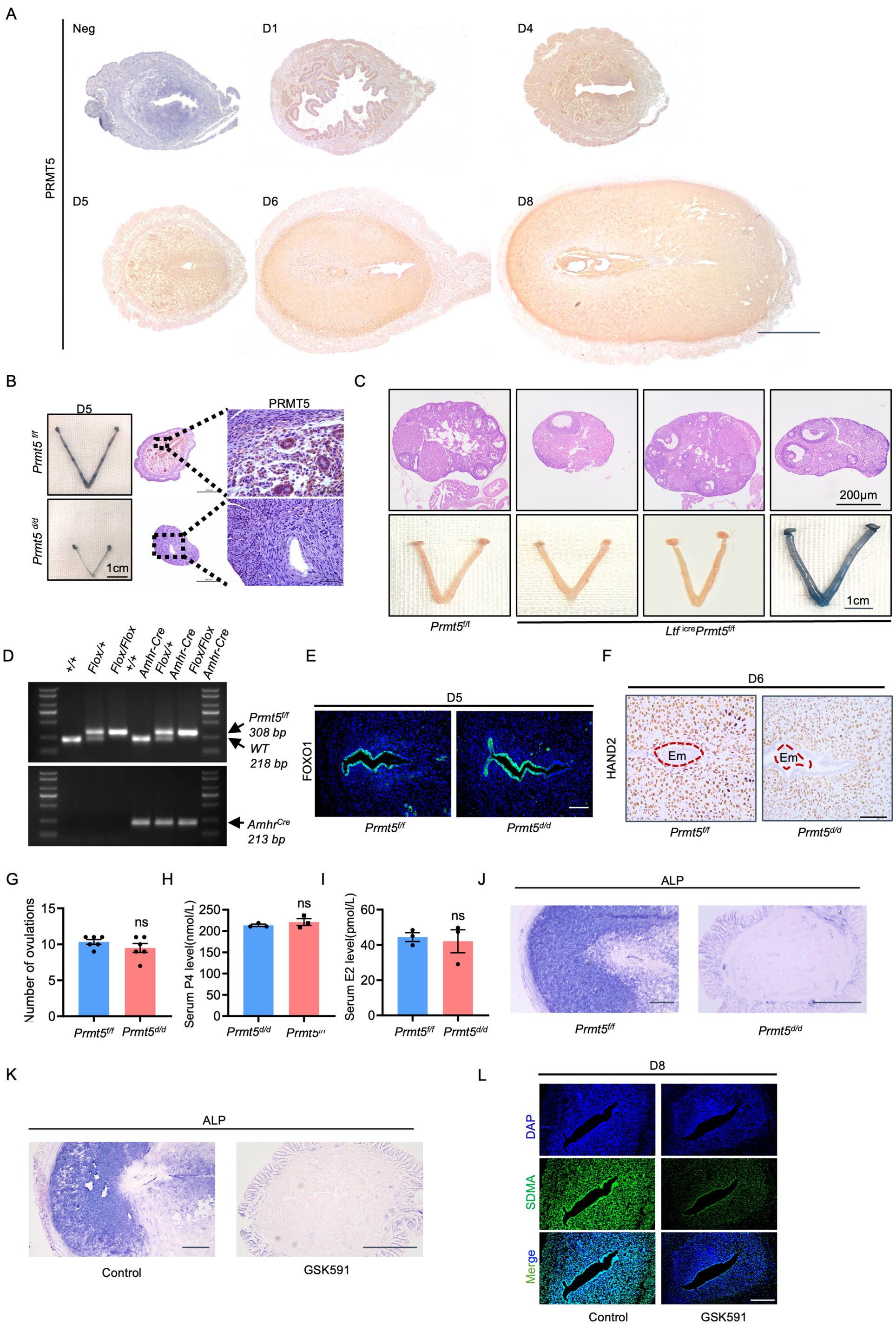

Supplemental Figure 3. Characterization of uterine PRMT5 expression and phenotypic analysis of stromal-specific *Prmt5* knockout mice. (A) Immunohistochemical analysis of PRMT5 protein expression in wild-type mouse uteri at different time points during pregnancy (D1–D8). Neg, negative control. Scale bar, 500 μm. (**B**) Gross morphology of uteri from 8-week-old $Prmt5^{f/f}$ and $Pgr^{cre}Prmt5^{f/f}$ mice. Scale bar, 5 mm. (**C**) Gross morphology of uteri and ovary from 8-week-old

$Prmt5^{f/f}$ and $Ltf^{cre}Prmt5^{f/f}$ mice. Scale bar, 1cm. (D) Genotyping PCR validation of $Prmt5^{f/f}$ and $Amhr2^{cre}Prmt5^{f/f}$ ($Prmt5^{d/d}$) mice. (**E**) Immunofluorescence staining of FOXO1 (green) in the implantation site at day 5 (D5) of pregnancy. (**F**) Immunohistochemical staining of HAND2 in the decidual zone at day 6 (D6) of pregnancy. Red dashed lines indicate the embryo (Em). (**G-I**) Comparison of basic reproductive parameters between $Prmt5^{f/f}$ and $Prmt5^{d/d}$ mice: number of ovulated oocytes (**G**), serum estradiol levels (**H**), and serum progesterone levels (**I**). (**J**) Alkaline phosphatase (ALP) activity staining of oil-induced artificial decidualization in $Prmt5^{f/f}$ and $Prmt5^{d/d}$ mice. (**K**) ALP activity staining of oil-induced artificial decidualization in Control and GSK591-treated mice. (**L**) Immunofluorescence analysis of symmetric dimethylarginine (SDMA, green) in decidual tissues at day 8 (D8) to validate the efficacy of GSK591 treatment. Nuclei were stained with DAPI (blue). Data represent mean ± SEM. ns, not significant, Student's t-test. Scale bar, 100 µm.

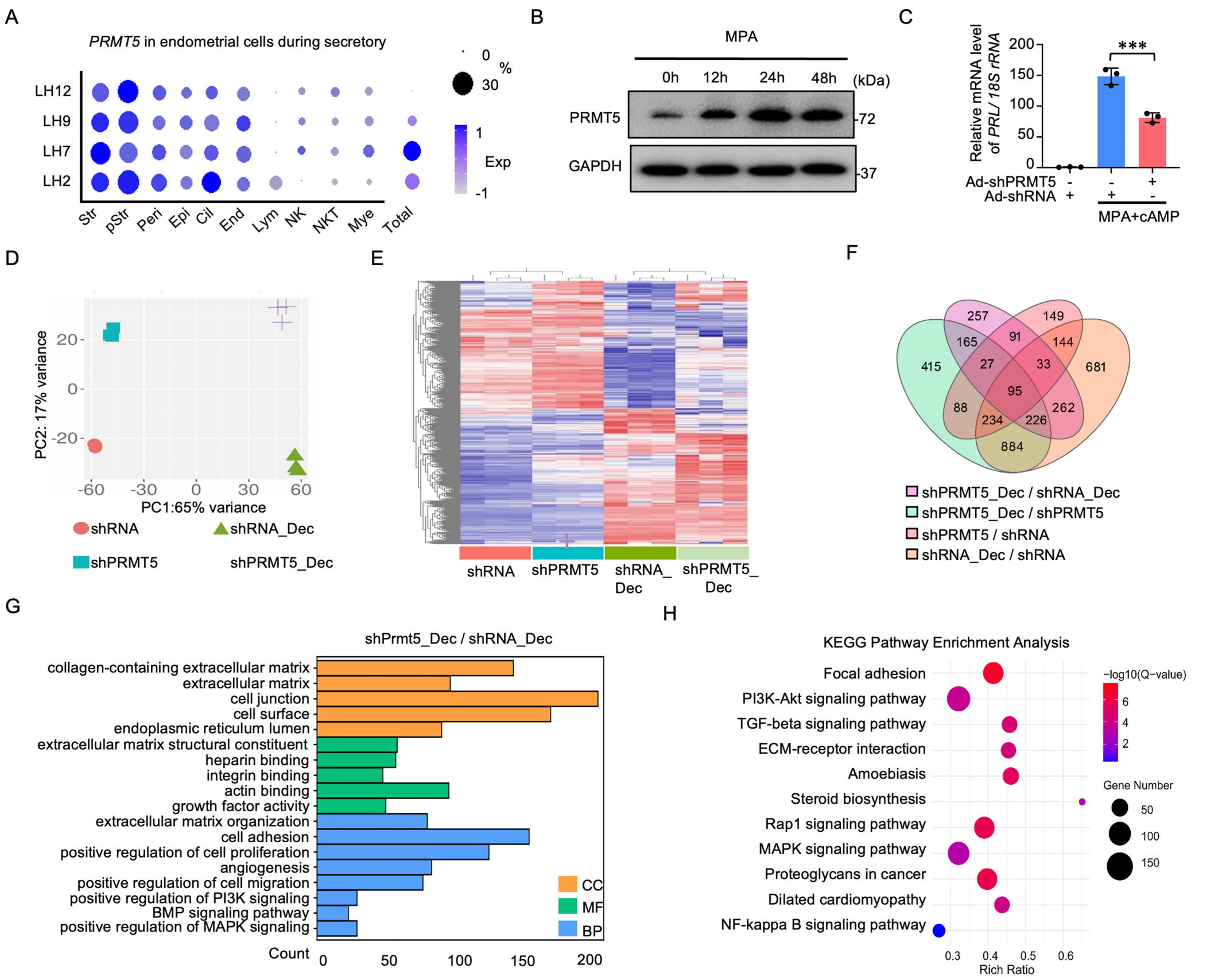

Supplemental Figure 4. Expression pattern of PRMT5 and transcriptomic analysis of PRMT5-knockdown EnSCs during decidualization. (**A**) Dot plot showing the expression levels of PRMT5 in different endometrial cell types across the secretory phase (LH+2 to LH+12) based on single-cell RNA sequencing (scRNA-seq) data. Cell types include stromal (Str), perivascular stromal (pStr), epithelial (Epi), ciliated (Cil), endothelial (End), lymphocyte (Lym), NK cells, etc. (**B**) Western blot analysis of PRMT5 protein levels in human endometrial stromal cells (EnSCs) treated with P4 for 0, 12, 24, and 48 h. (**C**) RT-qPCR analysis of the decidualization marker *PRL* mRNA levels following PRMT5 knockdown. (**D**) Principal component analysis (PCA) of transcriptomic data from four groups: shRNA (control), shPRMT5 (knockdown), shRNA_Dec (induced), and shPRMT5_Dec (induced knockdown). PC1 and PC2 explain 65% and 17% of the variance, respectively. (**E**) Hierarchical clustering heatmap of differentially expressed genes (DEGs) across the treatment groups. (**F**) Venn diagram illustrating the overlap of differentially expressed genes among the four

comparison combinations. (**G**) GO functional enrichment analysis of differentially expressed genes (DEGs) between the shPRMT5_Dec and shRNA_Dec groups, classified by cellular component (CC), molecular function (MF), and biological process (BP). (**H**) KEGG pathway enrichment analysis of the PRMT5 knockdown-associated DEGs. n=3. Data in (C) represent mean ± SEM (n = 3). ***P < 0.001.

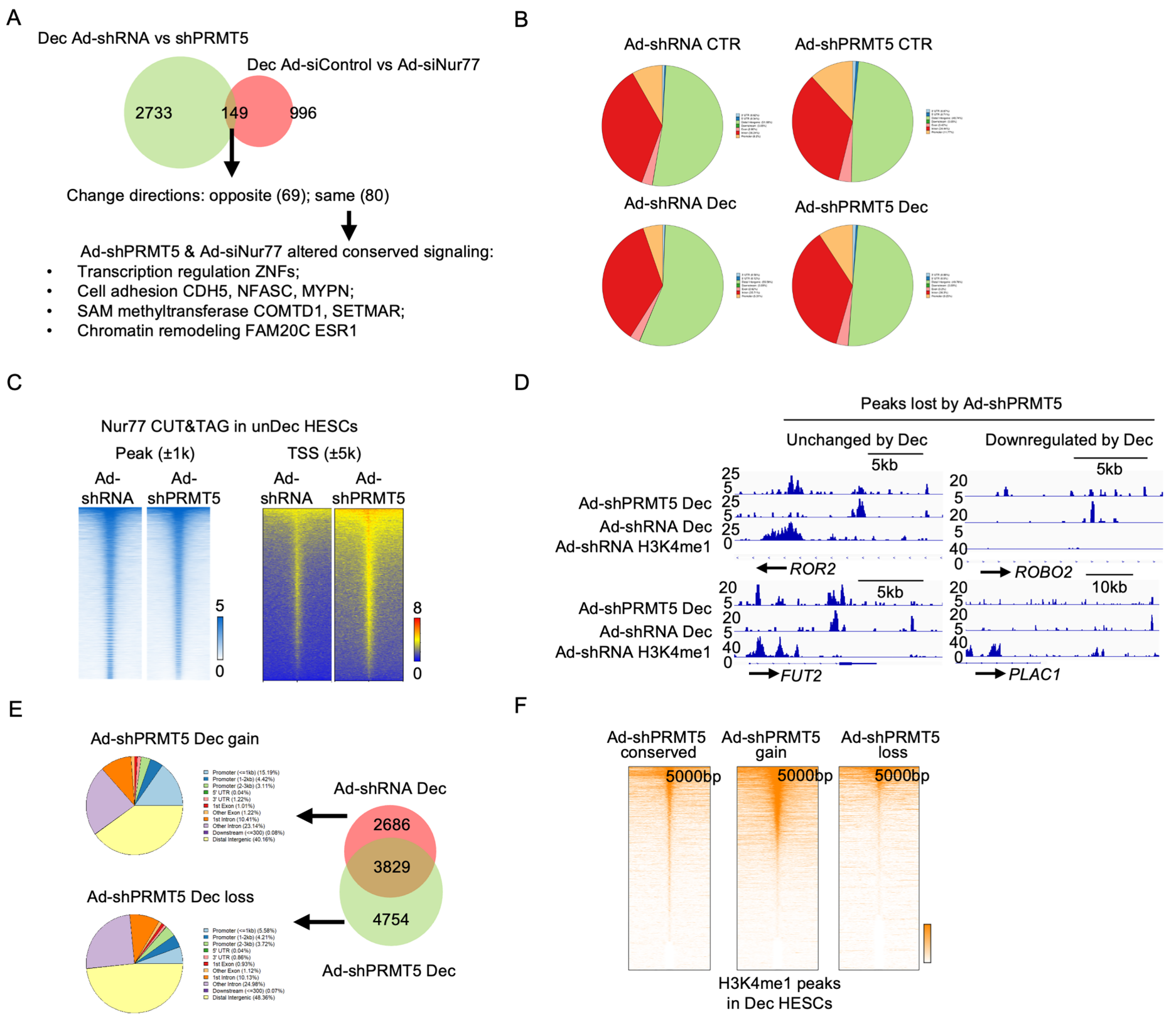

Supplemental Figure 5. Multi-omics integration, transcriptional overlap, and chromatin remodeling upon PRMT5 loss. (**A**) Venn diagram comparing differentially expressed genes (DEGs) in decidualized EnSCs following PRMT5 knockdown versus Nur77 knockdown. Common regulated pathways are listed below. (**B**) Pie charts showing the genomic distribution (promoter, intron, exon, distal intergenic) of Nur77 binding sites in control (Ad-shRNA) and PRMT5-knockdown (Ad-shPRMT5) EnSCs under proliferative (CTR) or decidualized (Dec) conditions. (**C**) Heatmaps of Nur77 CUT&Tag signal intensity centered on Nur77 peaks (left) and Transcription Start Sites (TSS, right) in undecidualized (unDec) EnSCs. (**D**) Representative genome browser tracks showing Nur77 occupancy at *ROR2*, *ROBO2*, *FUT2*, and *PLAC1* loci in decidualized EnSCs. Tracks highlight peaks lost upon PRMT5 knockdown. H3K4me1 track serves as a reference for enhancer regions. (**E**) Venn diagram analyzing the overlap of Nur77 binding peaks between control and PRMT5 knockdown decidualized EnSCs (Right), and pie charts showing the genomic annotation of gained and lost Nur77 binding sites upon PRMT5 knockdown (Left). (**F**)

Heatmaps displaying H3K4me1 signal intensity centered on Nur77 binding regions (conserved, gained, or lost) in decidualized EnSCs.

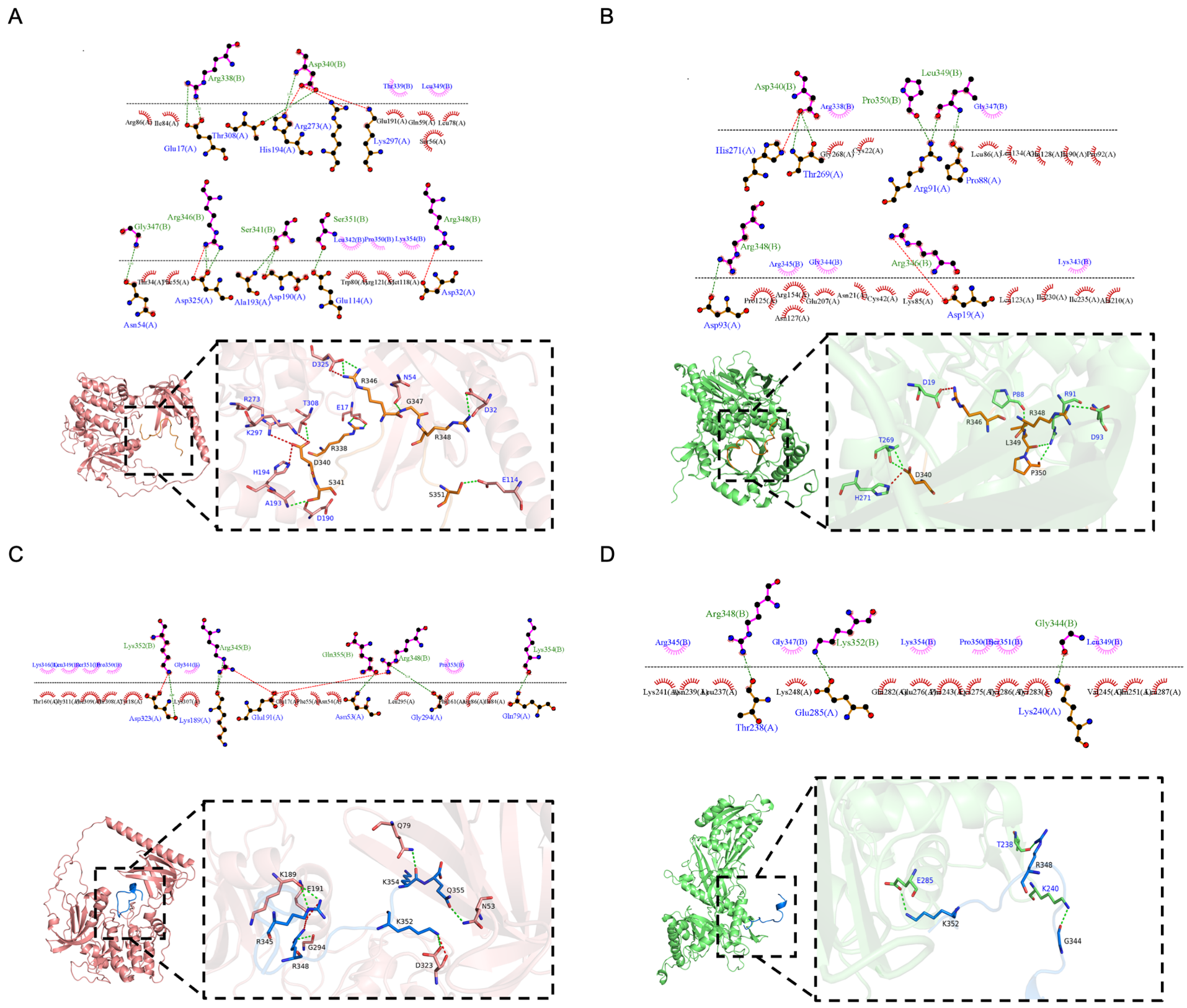

Supplemental Figure 6. Molecular docking analysis of Nur77 peptides interacting with PRMT5 and AKT proteins. (**A** and **B**) Predicted binding modes of the Pep-Nur77^{WT} with AKT (**A**) and PRMT5 (**B**). (**C** and **D**) Predicted binding modes of the mutant Pep-Nur77^{R346K} with AKT **(C)** and PRMT5 **(D)**. In each panel, the upper section displays the two-dimensional (2D) ligplot showing hydrogen bonds (green dashed lines) and hydrophobic interactions (red arcs), while the lower section displays the three-dimensional (3D) docking conformation. The dashed boxes in the 3D structures indicate the magnified view of the key binding interfaces and residues.

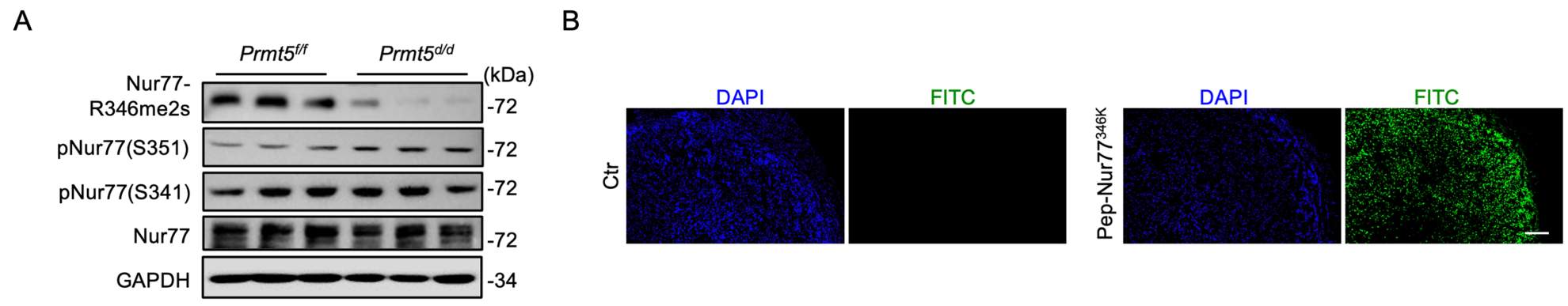

Supplemental Figure 7. Characterization and validation of in vivo peptide delivery and pharmacological models. (**A**) Western blot analysis of Nur77-R346me2s, pNur77(S351), and pNur77(S341) protein levels in endometrial tissues from *Prmt5*$^{f/f}$ and *Prmt5*$^{d/d}$ mice. (**B**) Validation of efficient delivery of FITC-labeled Pep-Nur77^{R346K} peptide into mouse uterine stromal cells via intrauterine perfusion. Scale bar, 100 μm.

A

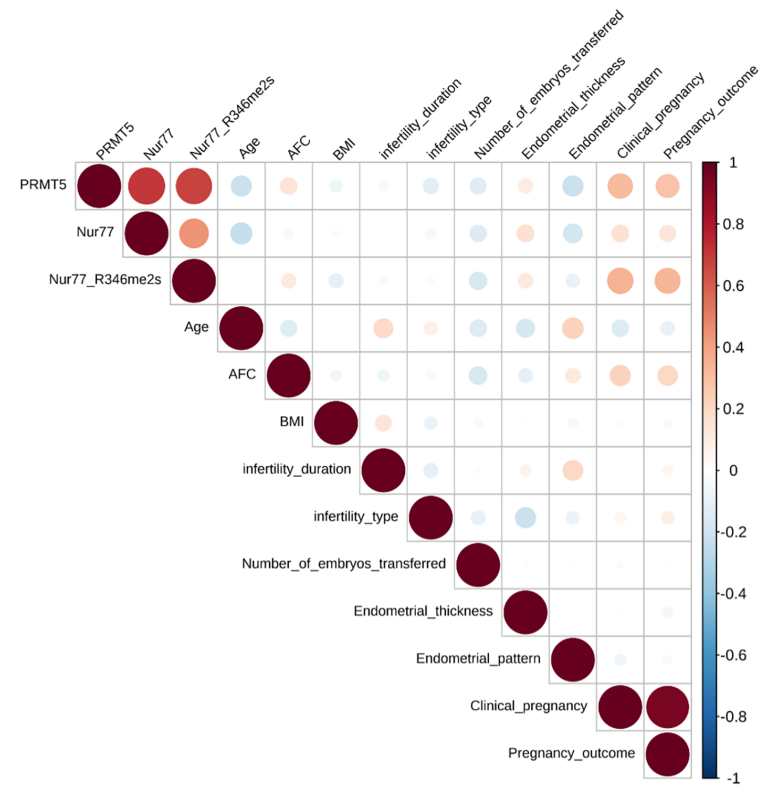

B

Variable	Model1 OR (95% CI)	P	Model2 OR (95% CI)	P	Model3 OR (95% CI)	P
PRMT5	6.3183 (2.2184, 17.9954)	0.00056	7.2784 (2.4380, 21.7290)	0.00038	6.6384 (2.1845, 20.1726)	0.00084
PRMT5 (T1)	Reference		Reference		Reference	
PRMT5 (T2)	1.3229 (0.5246, 3.3358)	0.55324	1.4388 (0.5633, 3.6748)	0.44697	1.4554 (0.5580, 3.7960)	0.44287
PRMT5 (T3)	4.1699 (1.6557, 10.5020)	0.00245	4.9030 (1.8625, 12.9074)	0.00129	4.6002 (1.6789, 12.6044)	0.00300
p for trend		0.00245		0.00129		0.00300

C

Variable	Model1 OR (95% CI)	P	Model2 OR (95% CI)	P	Model3 OR (95% CI)	P
Nur77-R346me2s	7.0497 (2.5793, 19.2683)	0.00014	8.3960 (2.9253, 24.0977)	0.00008	7.8819 (2.6628, 23.3311)	0.00019
Nur77-R346me2s (T1)	Reference		Reference		Reference	
Nur77-R346me2s (T2)	3.5763 (1.4303, 8.9424)	0.00642	3.5683 (1.4159, 8.9931)	0.00699	3.5543 (1.3955, 9.0528)	0.00784
Nur77-R346me2s (T3)	6.4613 (2.5213, 16.5583)	0.00010	7.1803 (2.7164, 18.9801)	0.00007	6.8043 (2.4664, 18.7719)	0.00021
p for trend		0.00010		0.00007		0.00021

Supplemental Figure 8. Correlation and multivariable logistic regression analysis of endometrial PRMT5, Nur77, and Nur77-R346me2s with pregnancy outcome. (**A**) Spearman's rank correlation matrix. (**B**) Multivariable logistic regression evaluating the association between PRMT5 and pregnancy outcome. PRMT5 was analyzed both as a continuous predictor and as tertiles (T1: 0.02–0.46; T2: 0.46–0.83; T3: 0.83–1.87), with T1 as the reference category; Model 1–3 included different sets of confounders. (**C**) Multivariable logistic regression evaluating the association between Nur77-R346me2s and pregnancy outcome. Nur77-R346me2s was analyzed as tertiles (T1: 0.01–0.34; T2: 0.34–0.77; T3: 0.77–1.94), with T1 as the reference category; Model 1–3 included different sets of confounders.

A

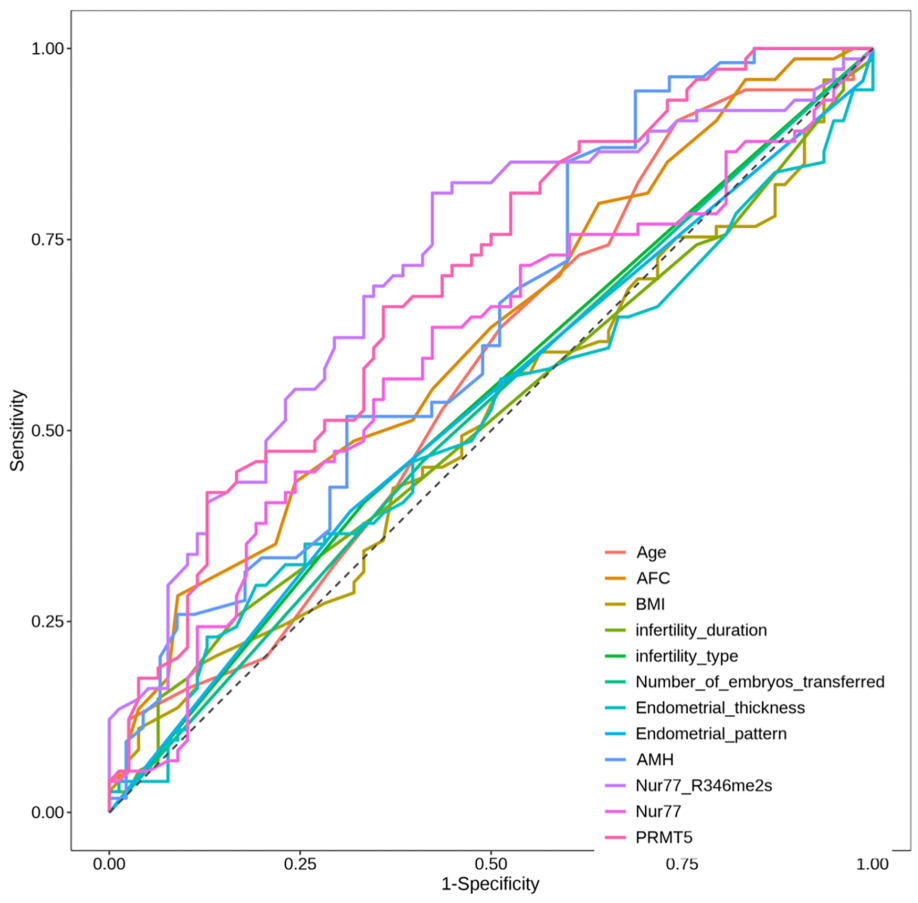

B

Variable	AUC	95% CI (Lower)	95% CI (Upper)	Predicted probability threshold	Specificity	Sensitivity	Youden index	Cut-off value (original scale)
Nur77-R346me2s	0.71	0.627	0.793	0.38	0.5769	0.8108	0.3877	0.3683
PRMT5	0.694	0.611	0.777	0.4553	0.641	0.6622	0.3032	0.5955
Nur77	0.596	0.504	0.688	0.469	0.5769	0.6351	0.2121	0.6364
AMH	0.634	0.523	0.745	0.4336	0.3111	0.9444	0.2556	1.4251
AFC	0.623	0.534	0.711	0.5983	0.9103	0.2838	0.194	23.4982
Age	0.573	0.482	0.664	0.4146	0.2564	0.9054	0.1618	35.4986
infertility_type	0.536	0.459	0.613	0.497	0.6667	0.4054	0.0721	1.5002
Endometrial_pattern	0.529	0.449	0.609	0.4908	0.6842	0.3944	0.0786	1.4996
Number_of_embryos_transferred	0.525	0.445	0.604	0.4901	0.5897	0.4595	0.0492	1.5005
infertility_duration	0.516	0.424	0.608	0.5013	0.8333	0.2568	0.0901	4.5
BMI	0.509	0.416	0.602	0.4869	0.8846	0.1918	0.0764	19.45
Endometrial_thickness	0.508	0.415	0.602	0.5028	0.8077	0.2973	0.105	11.75

Supplemental Figure 9. ROC curve analysis evaluating the predictive performance of endometrial molecular markers and clinical variables for pregnancy outcome. (**A**) Univariable ROC curves evaluating the discriminative performance of individual predictors. (**B**) Summary of univariable ROC performance metrics.

A

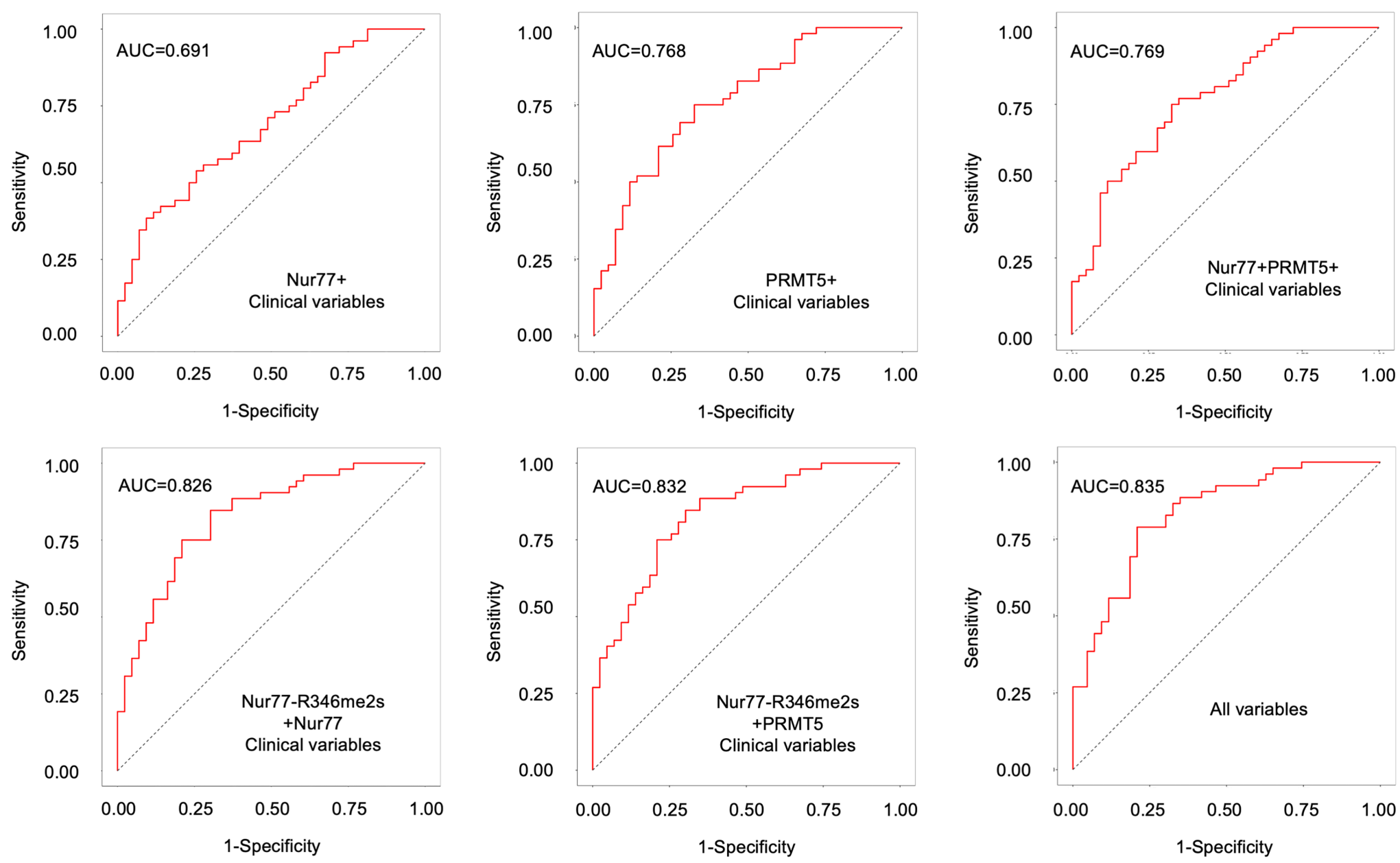

B

Variable	AUC	95% CI (Lower)	95% CI (Upper)	Predicted probability threshold	Specificity	Sensitivity	Youden index
Clinical variables	0.679	0.572	0.787	0.5103	0.5349	0.7692	0.3041
PRMT5+Clinical variables	0.768	0.673	0.862	0.5322	0.6744	0.75	0.4244
Nur77+Clinical variables	0.691	0.585	0.796	0.6675	0.907	0.3846	0.2916
Nur77-R346me2s+Clinical variables	0.828	0.745	0.91	0.501	0.6977	0.8462	0.5439
PRMT5+Nur77+Clinical variables	0.769	0.675	0.864	0.523	0.6744	0.75	0.4244
Nur77-R346me2s+PRMT5+Clinical variables	0.832	0.752	0.913	0.4794	0.6977	0.8462	0.5439
Nur77-R346me2s+Nur77+Clinical variables	0.826	0.743	0.909	0.491	0.6977	0.8462	0.5439
All variables	0.835	0.754	0.915	0.5313	0.7907	0.7885	0.5792

Supplemental Figure 10. Multivariable ROC analysis of clinical variables combined with endometrial PRMT5-Nur77 methylation markers for predicting pregnancy outcomes. (**A**) Receiver operating characteristic (ROC) curve analysis of clinical variables, PRMT5, Nur77, and Nur77-R346me2s for predicting pregnancy outcomes. (**B**) Summary of multivariable ROC performance for models incorporating clinical variables with or without endometrial molecular markers.

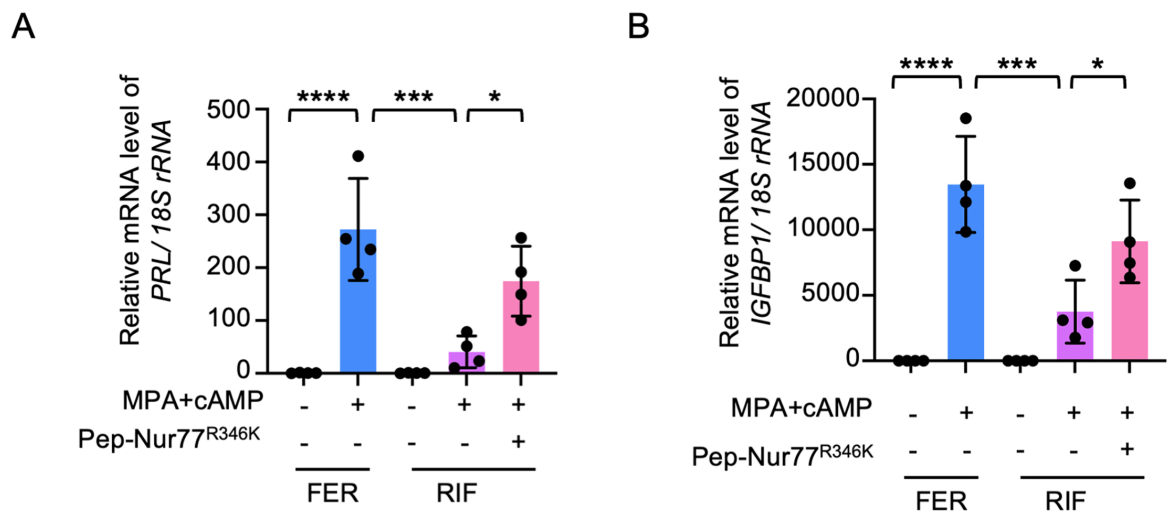

Supplemental Figure 11. Pep-Nur77^{R346K} reproducibly improves decidual marker induction in RIF-derived EnSCs. (**A** and **B**) RT-qPCR analysis of decidualization-associated *PRL* (**A**) and *IGFBP1* (**B**) mRNA expression in primary EnSCs from FER and RIF participants following decidualization induction with MPA+cAMP, with or without Pep-Nur77^{R346K} treatment. Data represent mean ± SEM; n = 4 independent participants per group. *P < 0.05; ***P < 0.001; ****P < 0.0001. One-way ANOVA with Tukey's multiple comparisons test.
