# Supplementary material for: Targeting the PRMT5/Nur77 methylation axis enhances endometrial decidualization capacity and female fertility in preclinical models
Source: J Clin Invest. 2026 Jul 16;136(17):e178862. doi: 10.1172/JCI178862 (PMC13528935; doi:10.1172/JCI178862)
Supplement: Supplementary file 2 [file jci-136-178862-s192.pdf]

Fig1. C

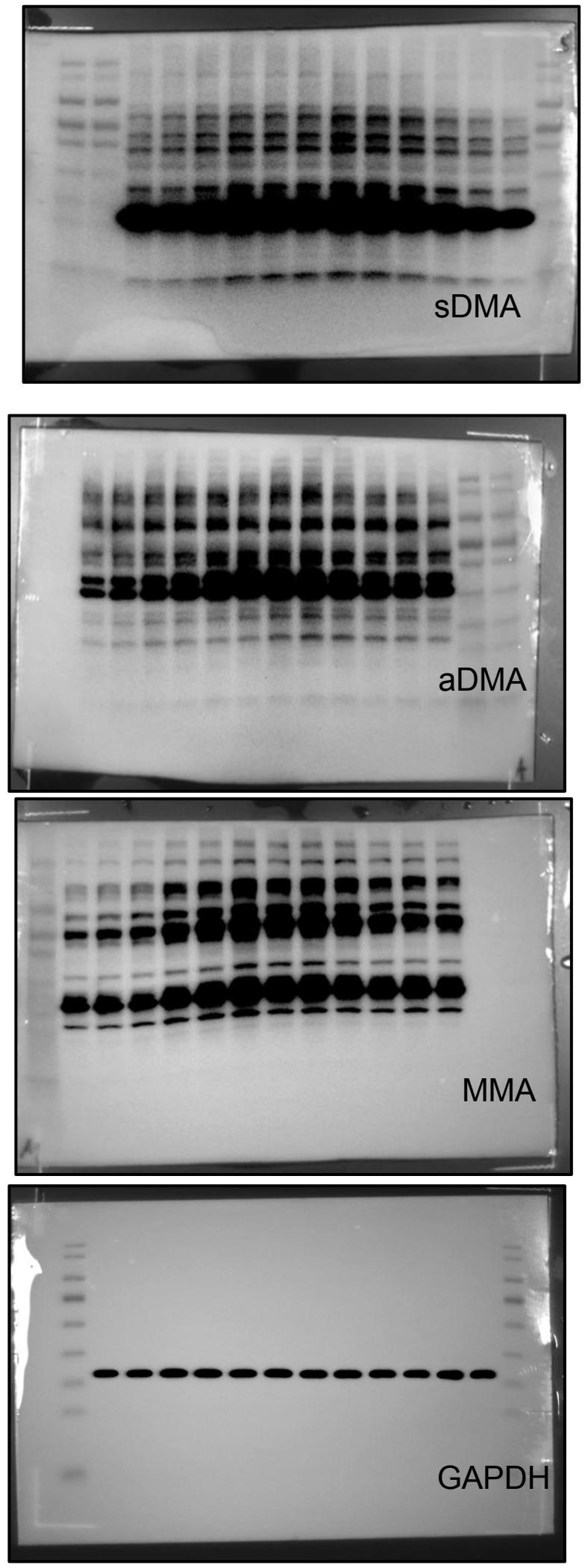

Fig1. D

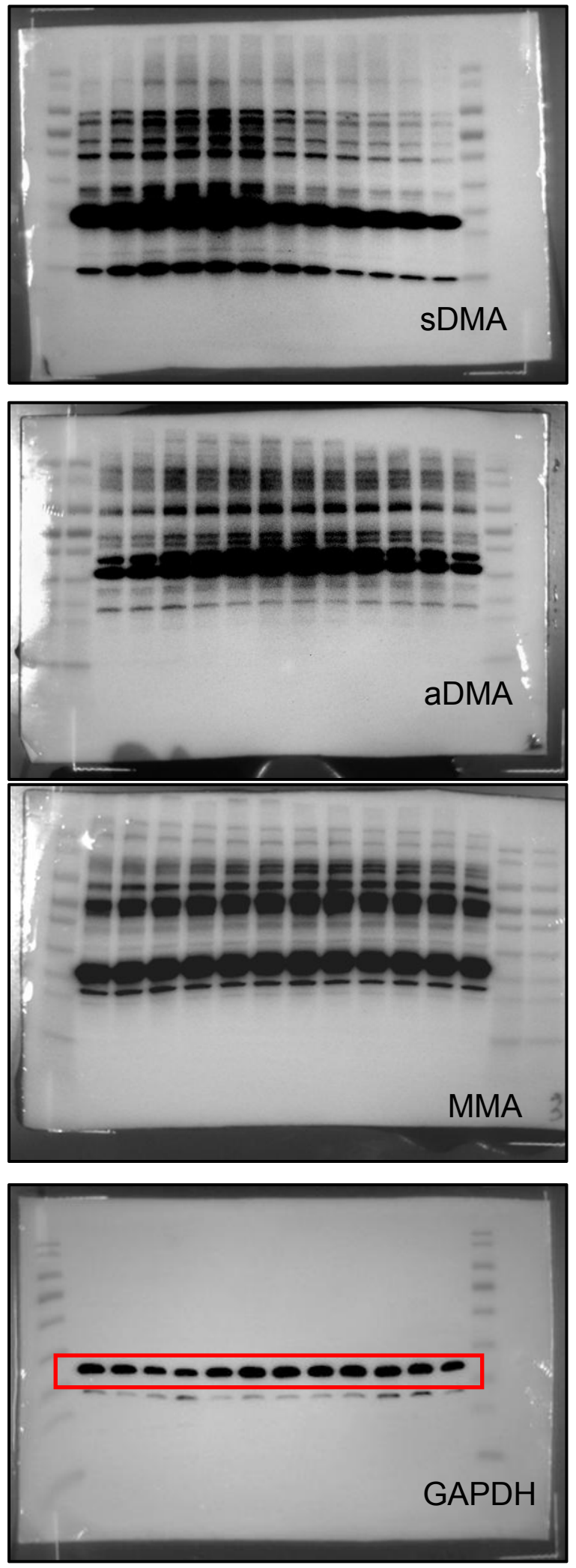

Fig1. I

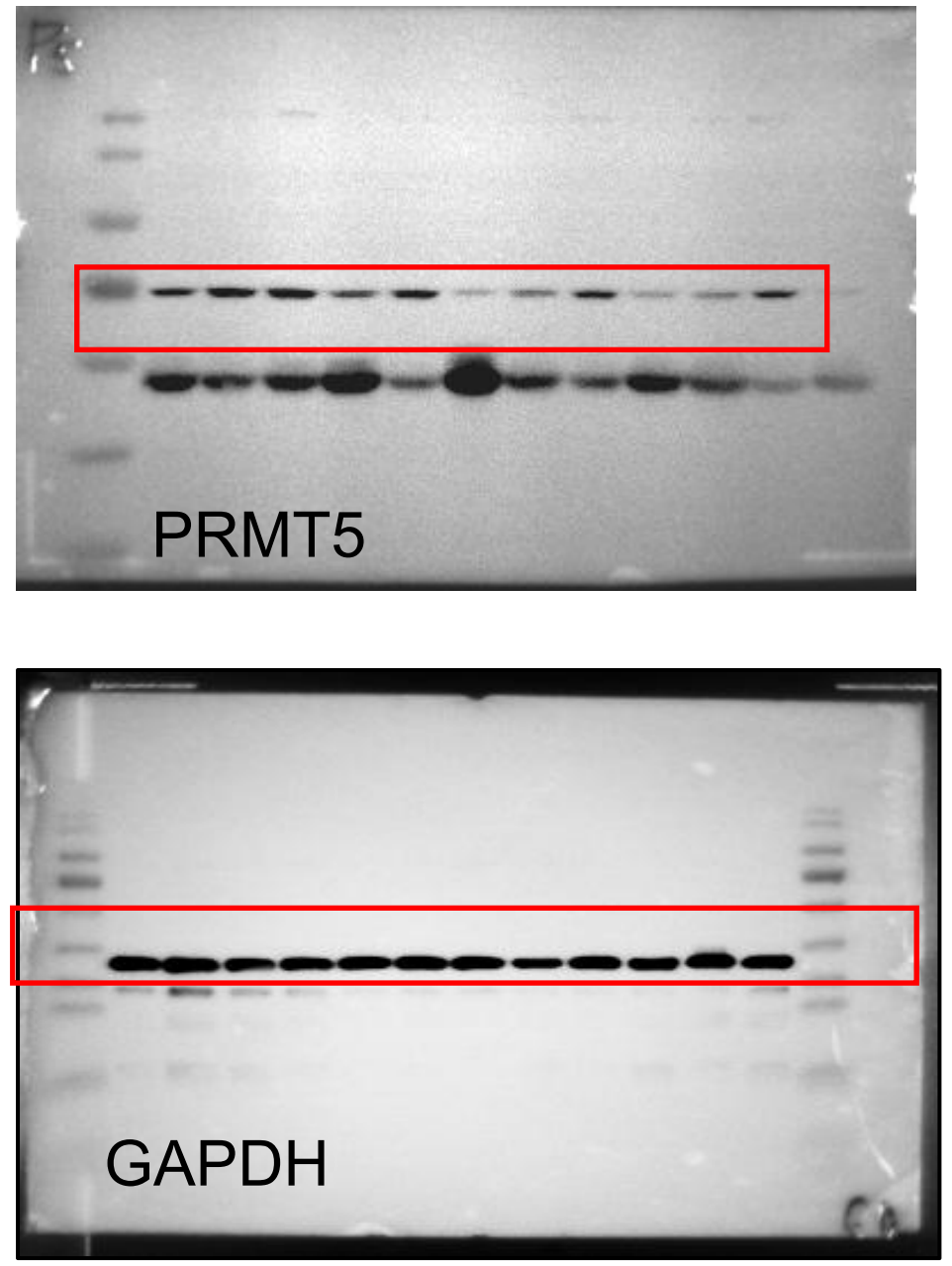

Fig2. C

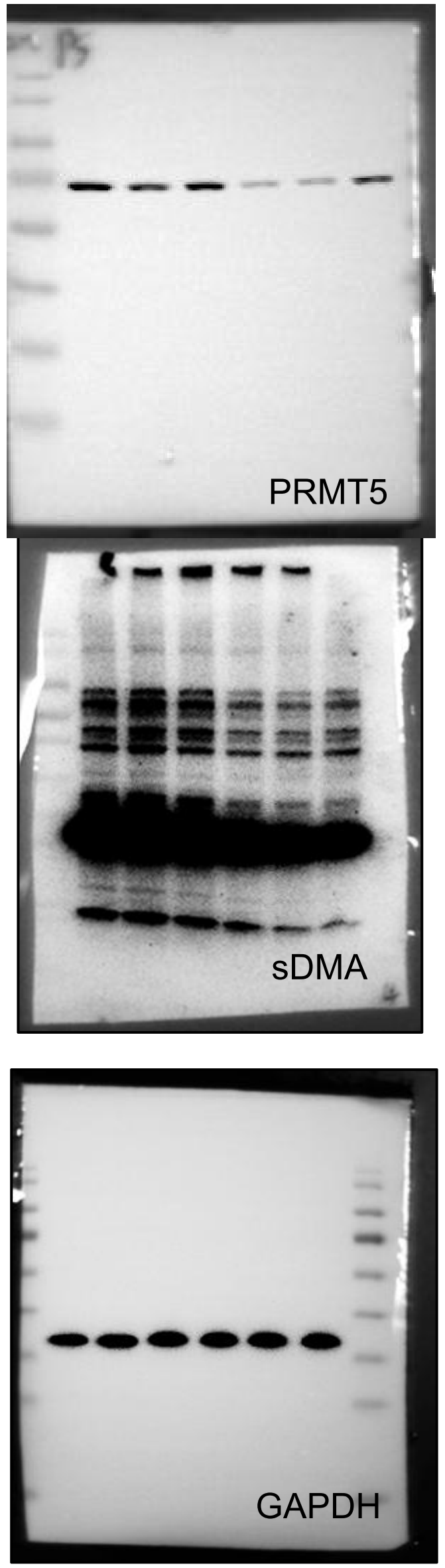

Fig3. B

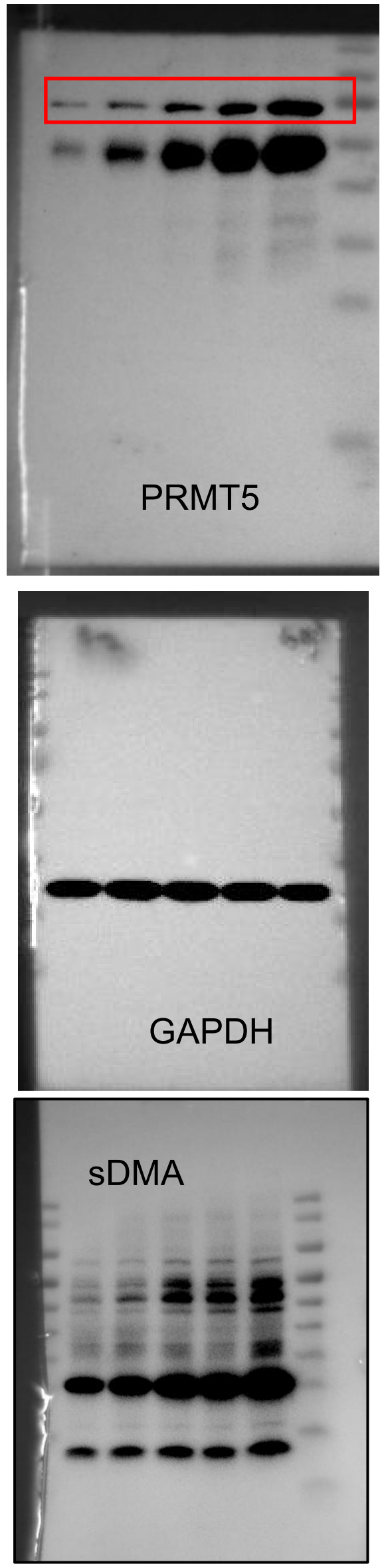

Fig3. D

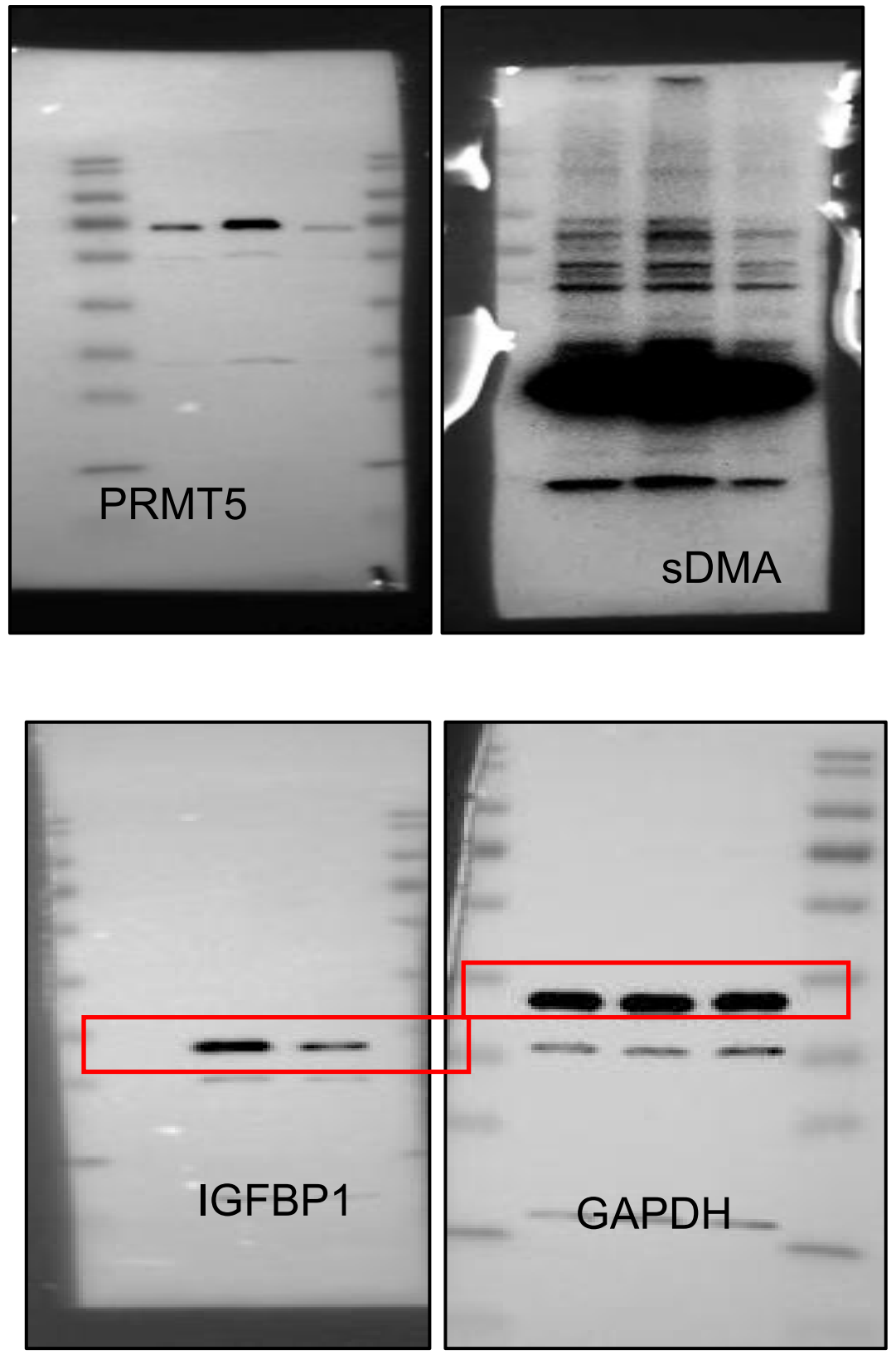

Supplemental Fig. 4B

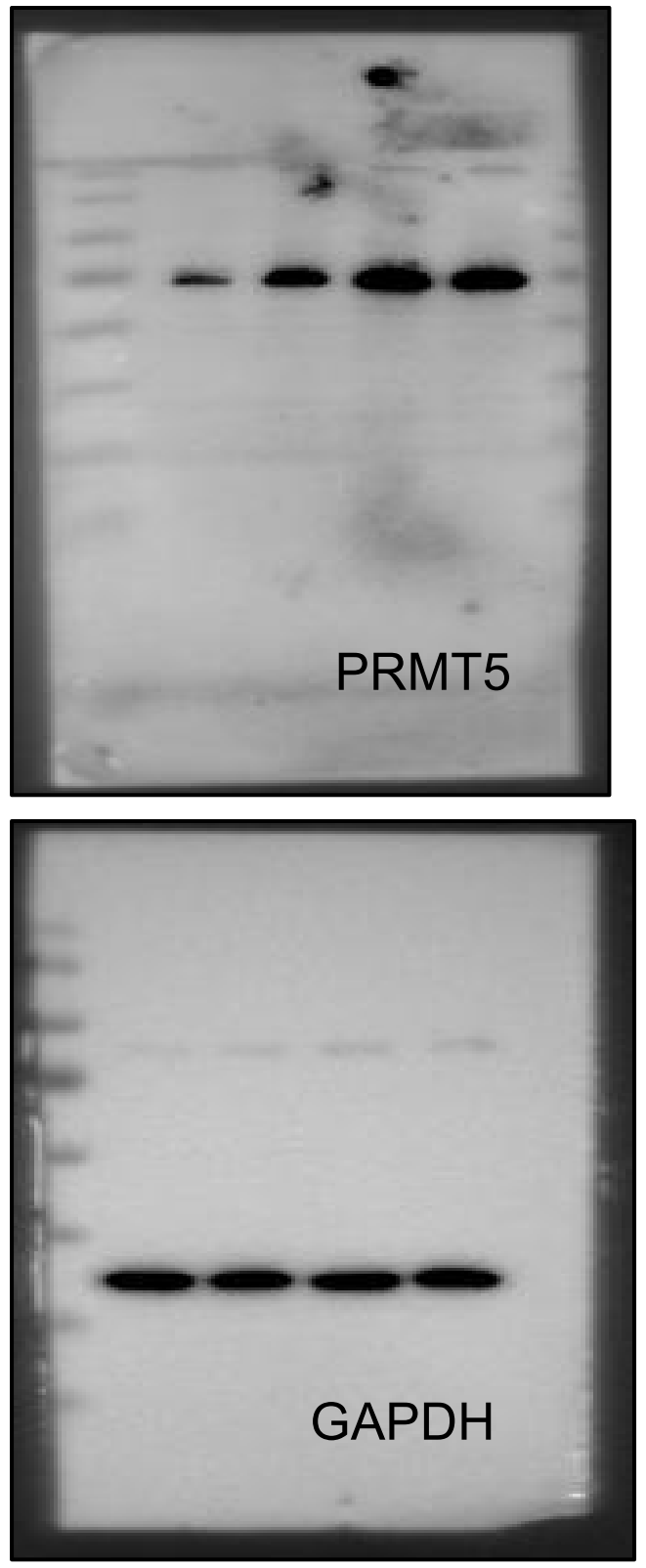

Fig4. E

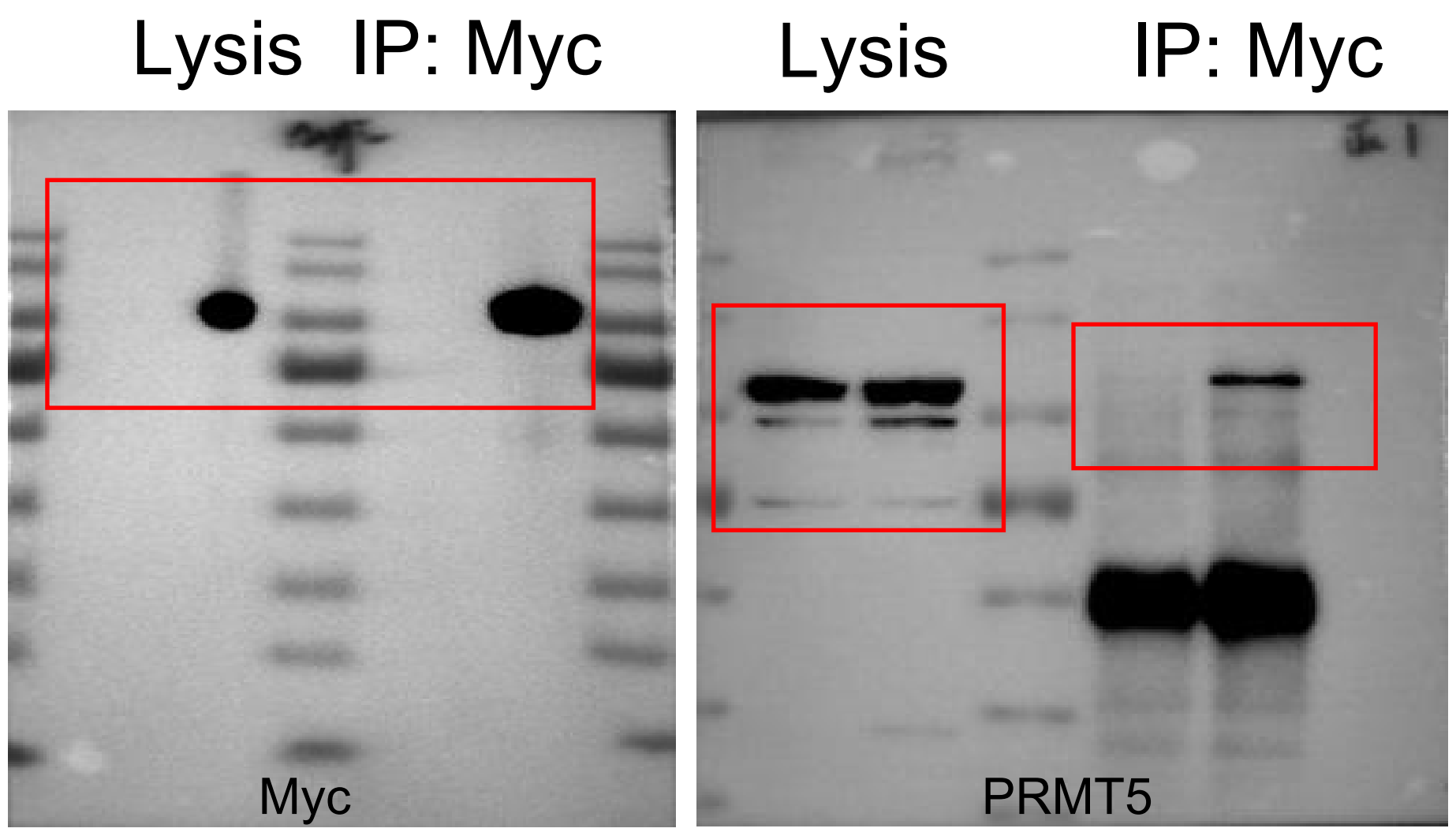
Lysis
IP: Myc
Lysis
IP: Myc
Myc
PRMT5

Fig4. F

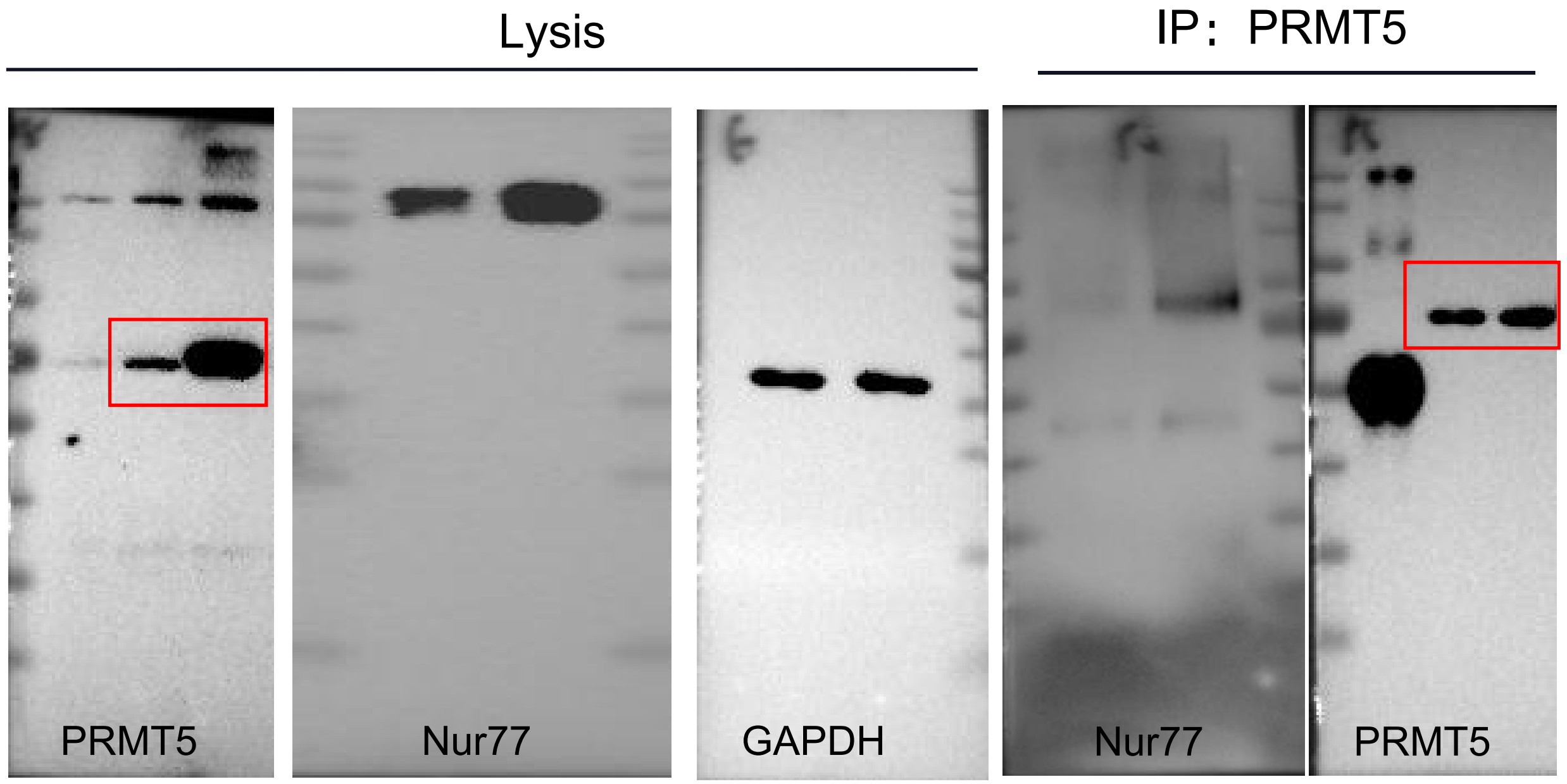
Lysis
IP: PRMT5
PRMT5
Nur77
GAPDH
Nur77
PRMT5

Fig4. G

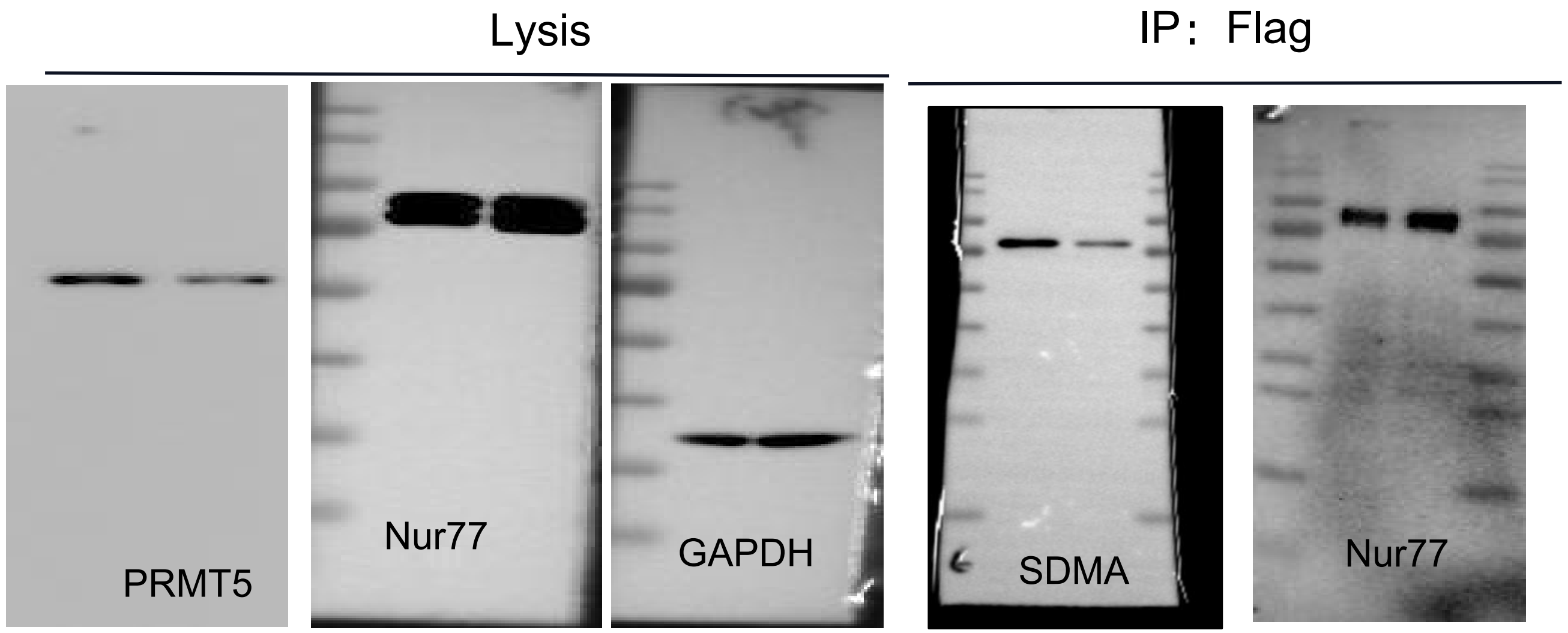

Fig5. A

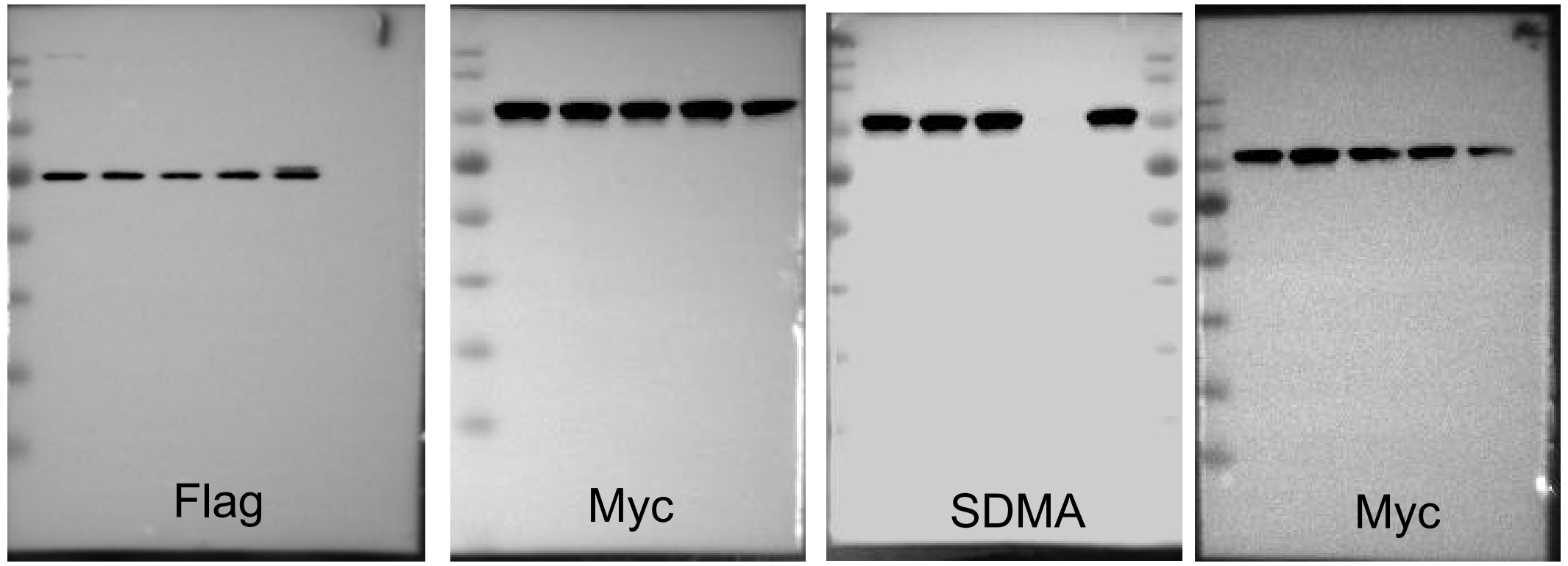

Fig5. B

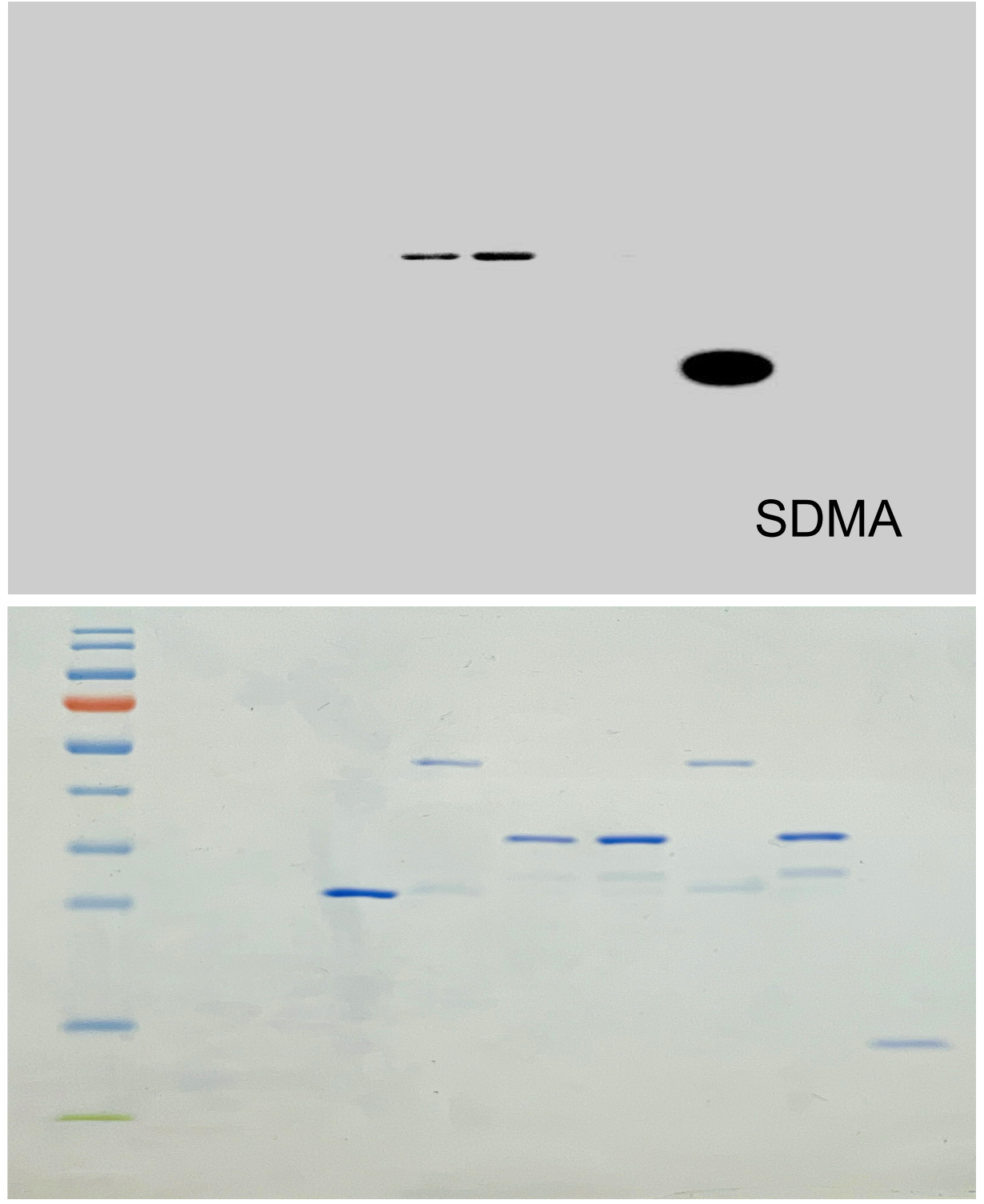

Fig5. C

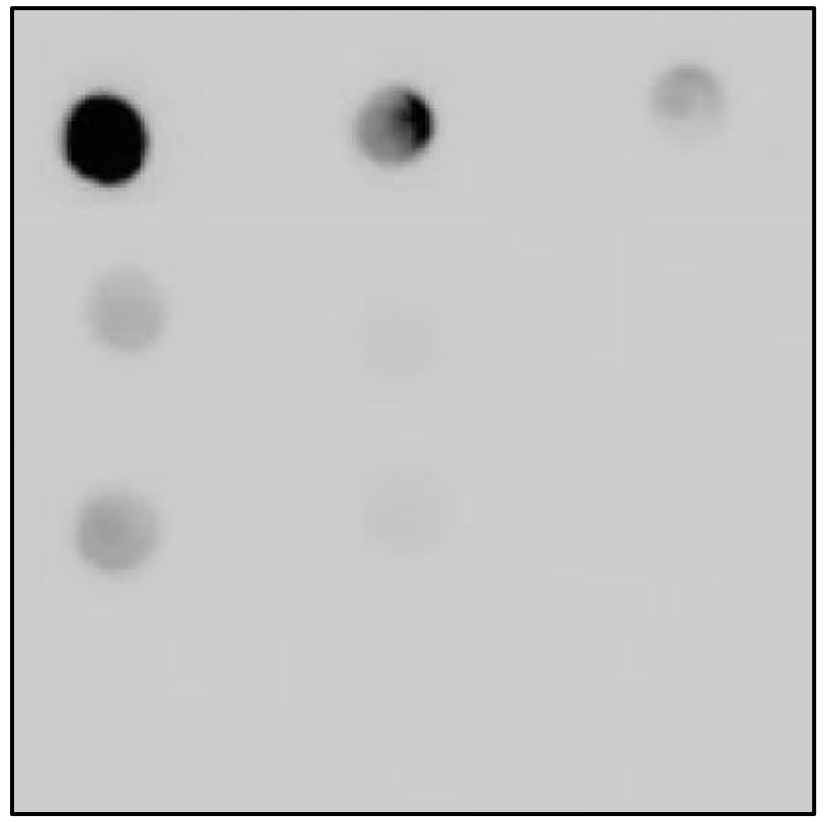

Fig5. D

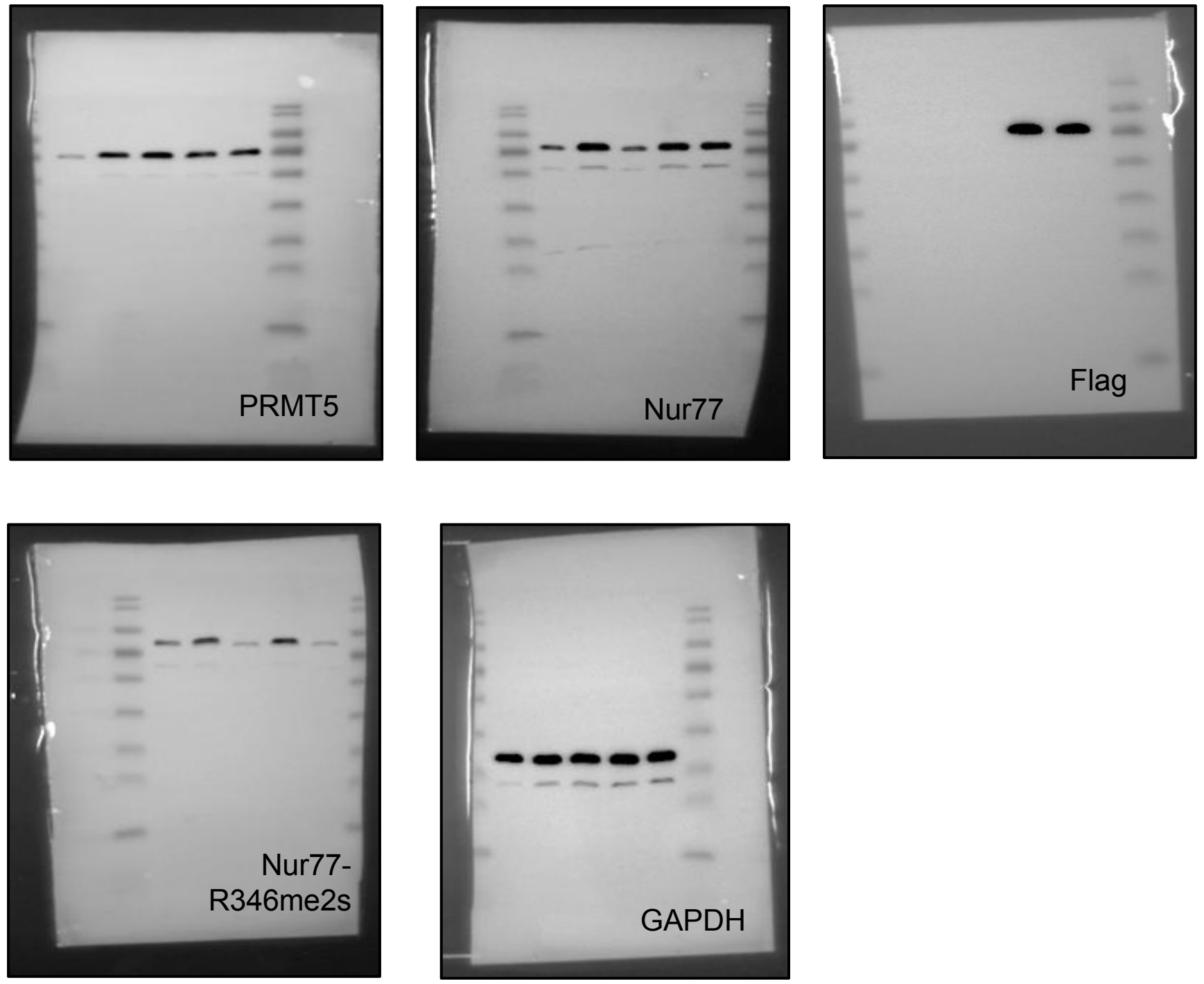
PRMT5
Nur77
Flag
Nur77-
R346me2s
GAPDH

Fig5. G

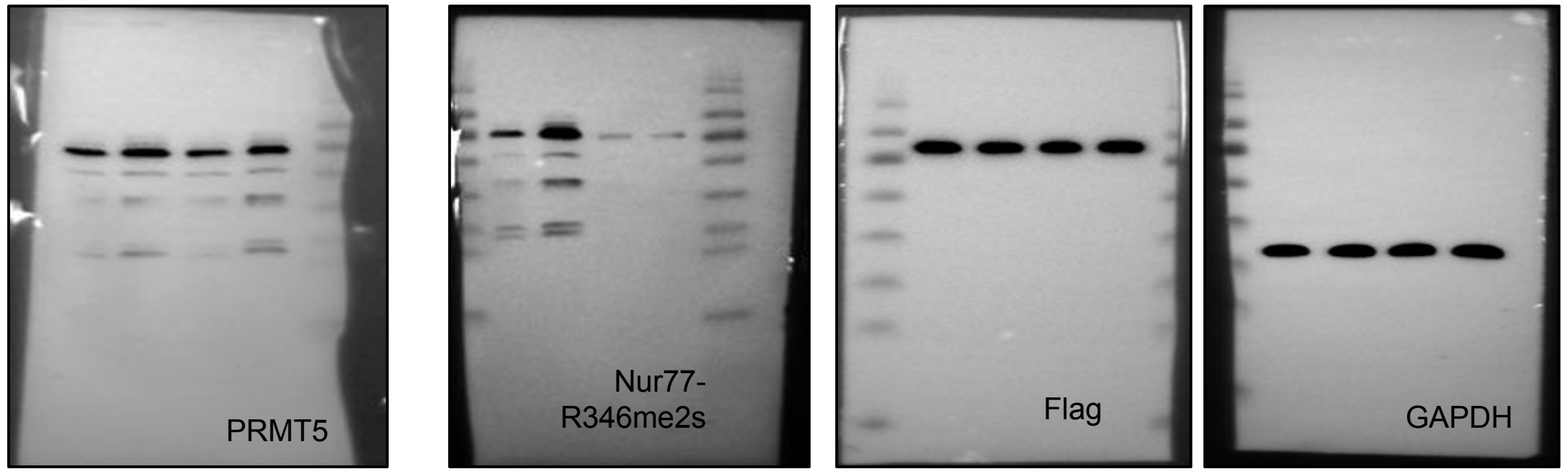
PRMT5
Nur77-
R346me2s
Flag
GAPDH

Fig6. F

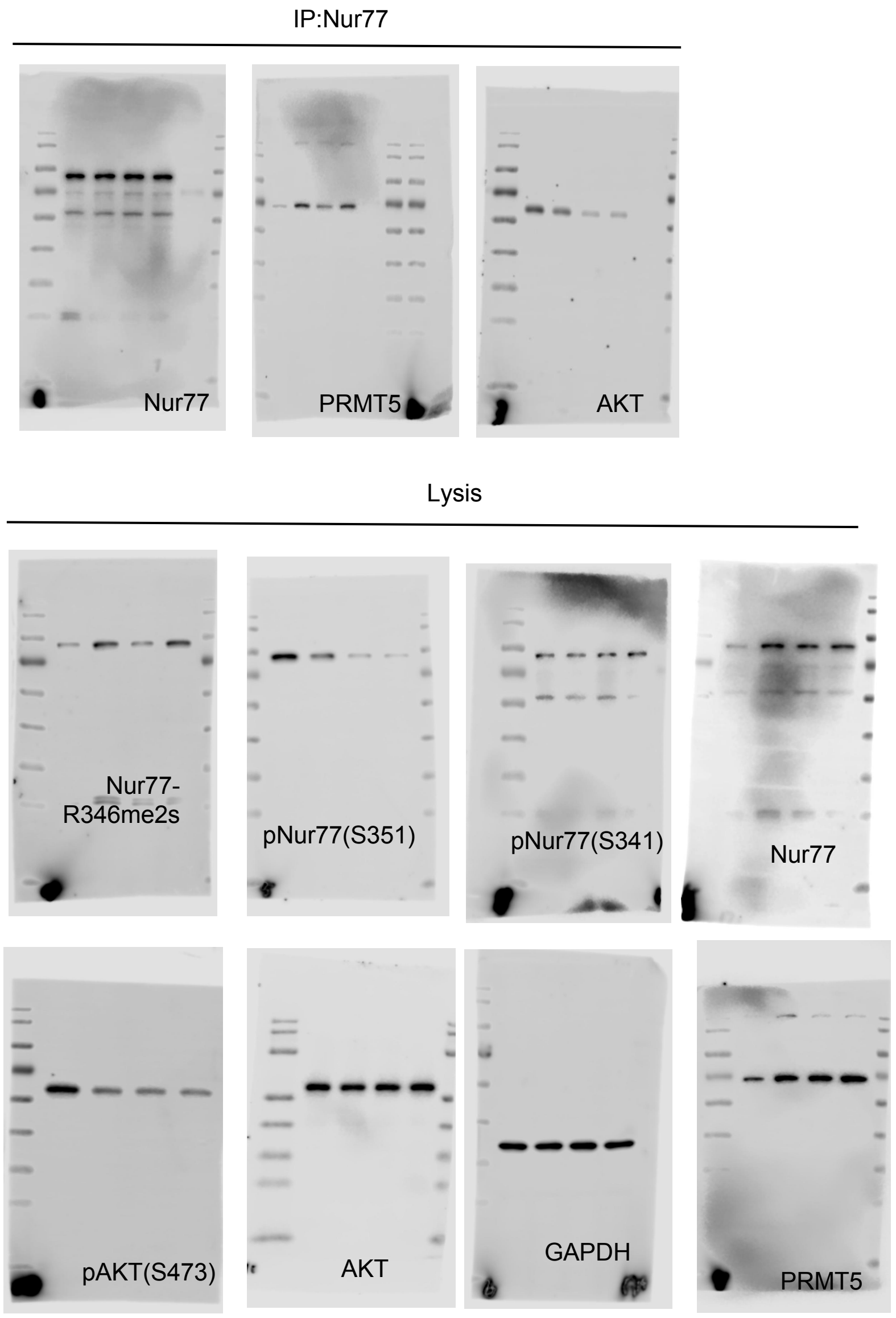

Fig6. G

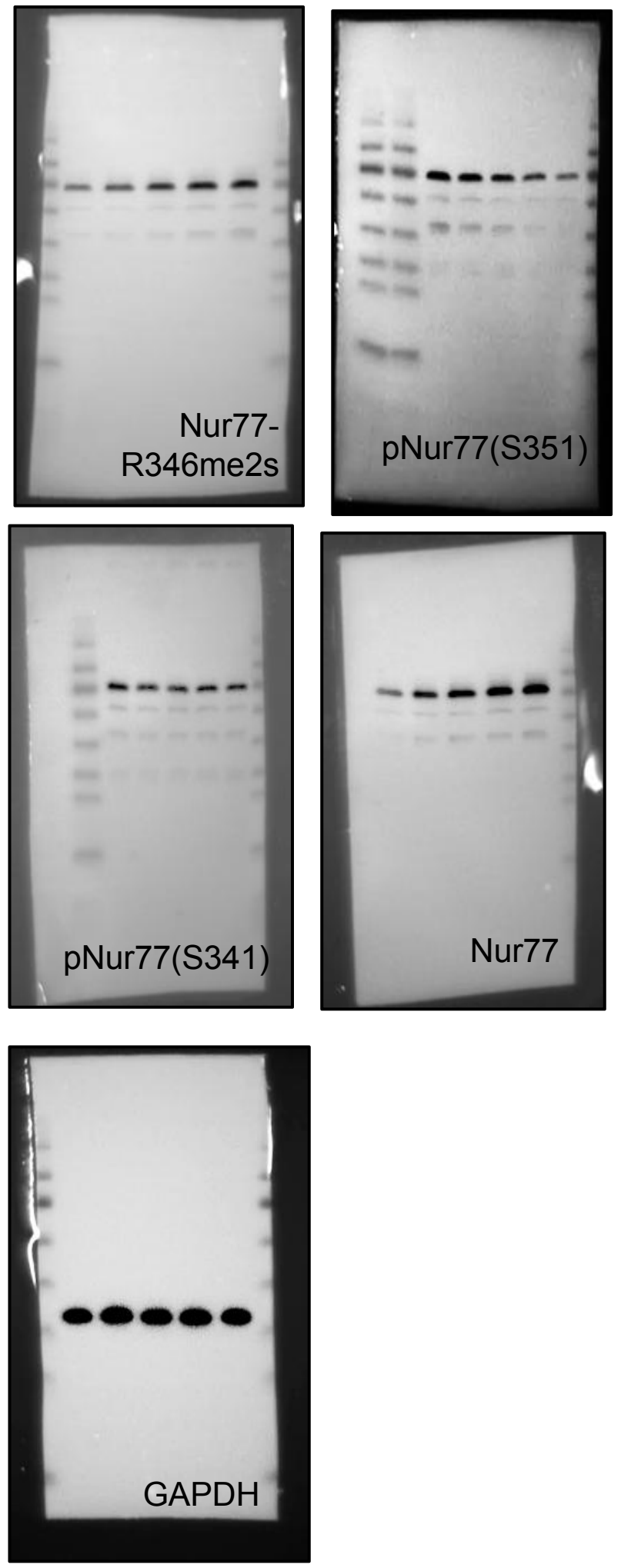

Fig6. J

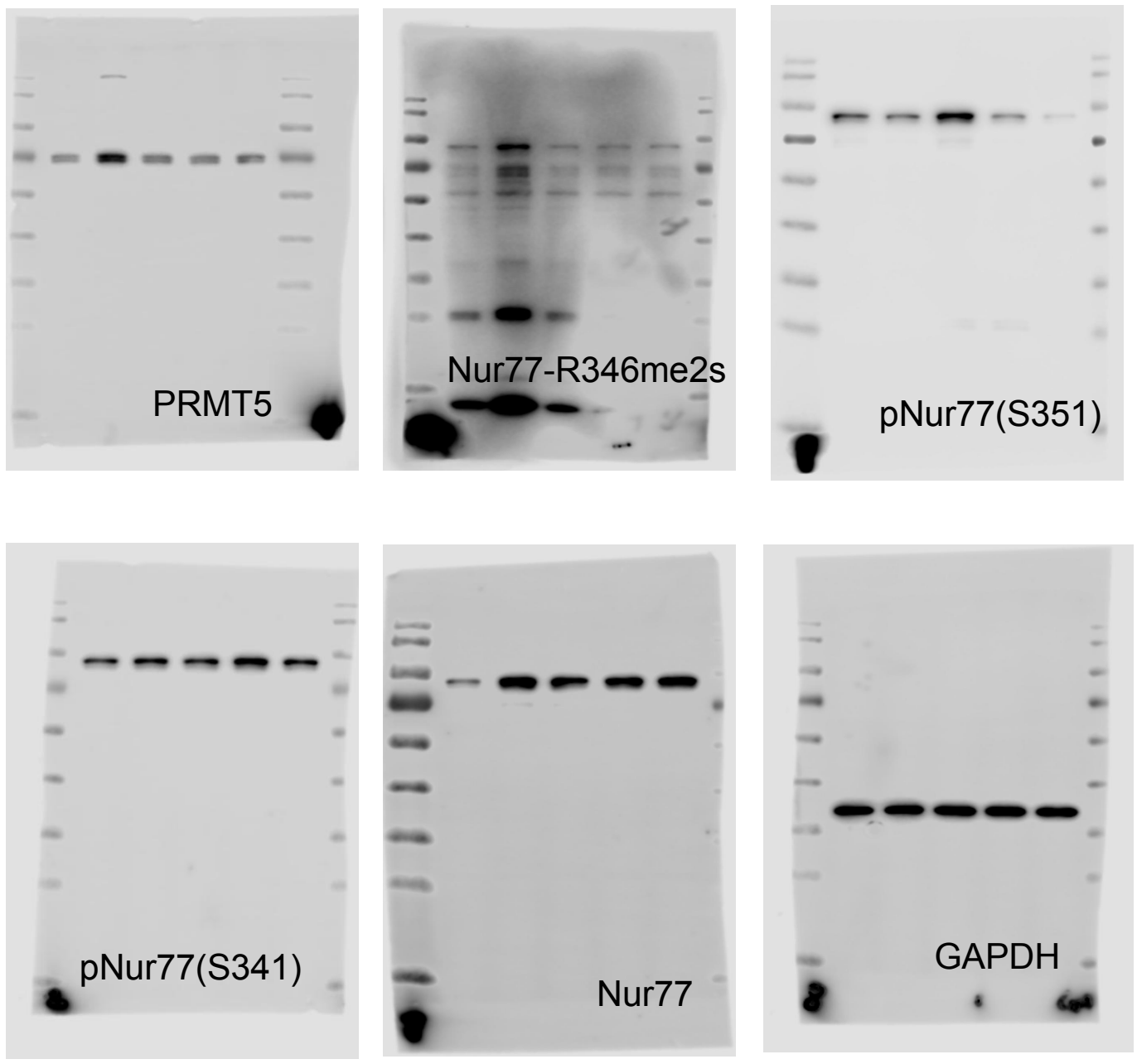

Fig7. B

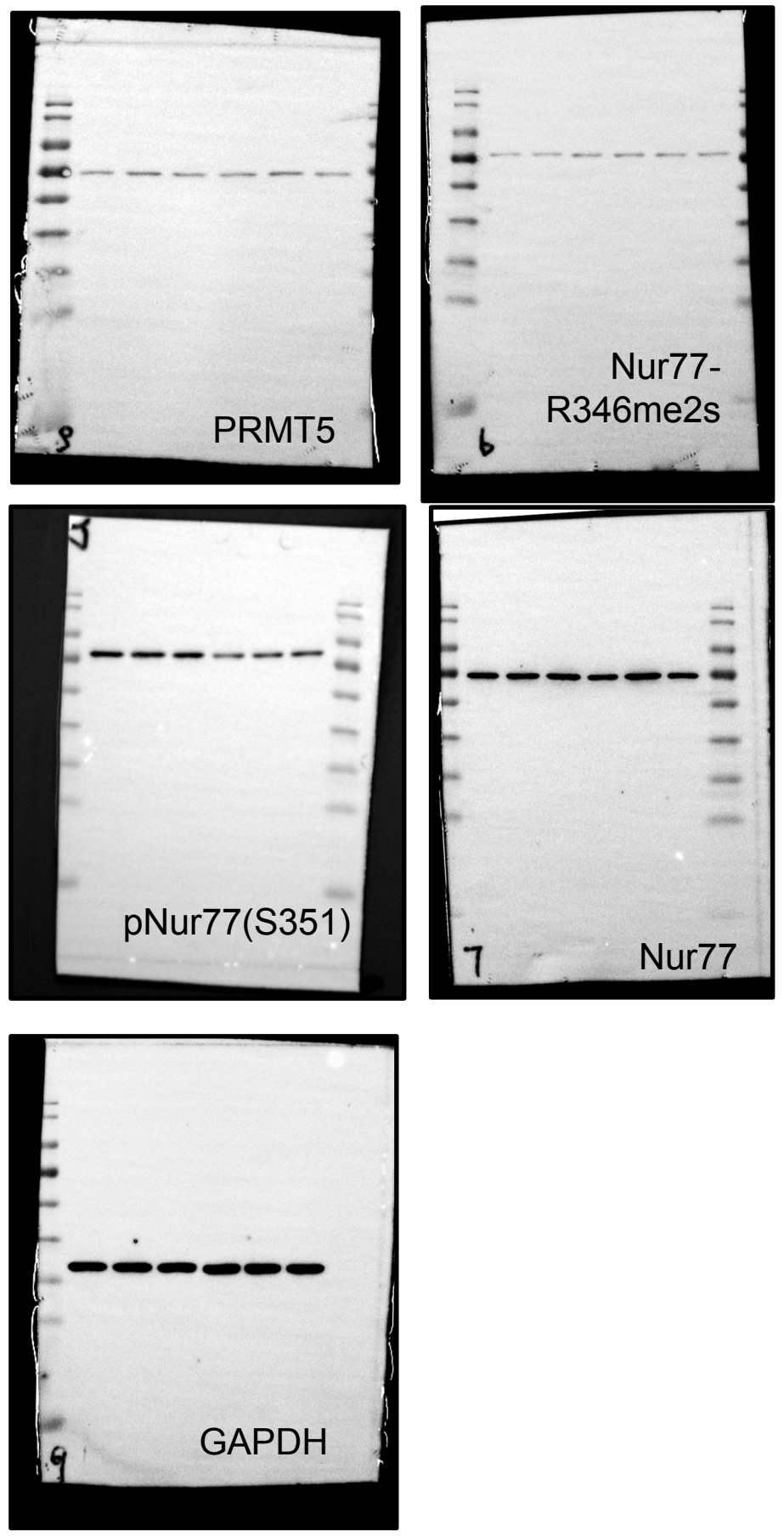

Fig7. H

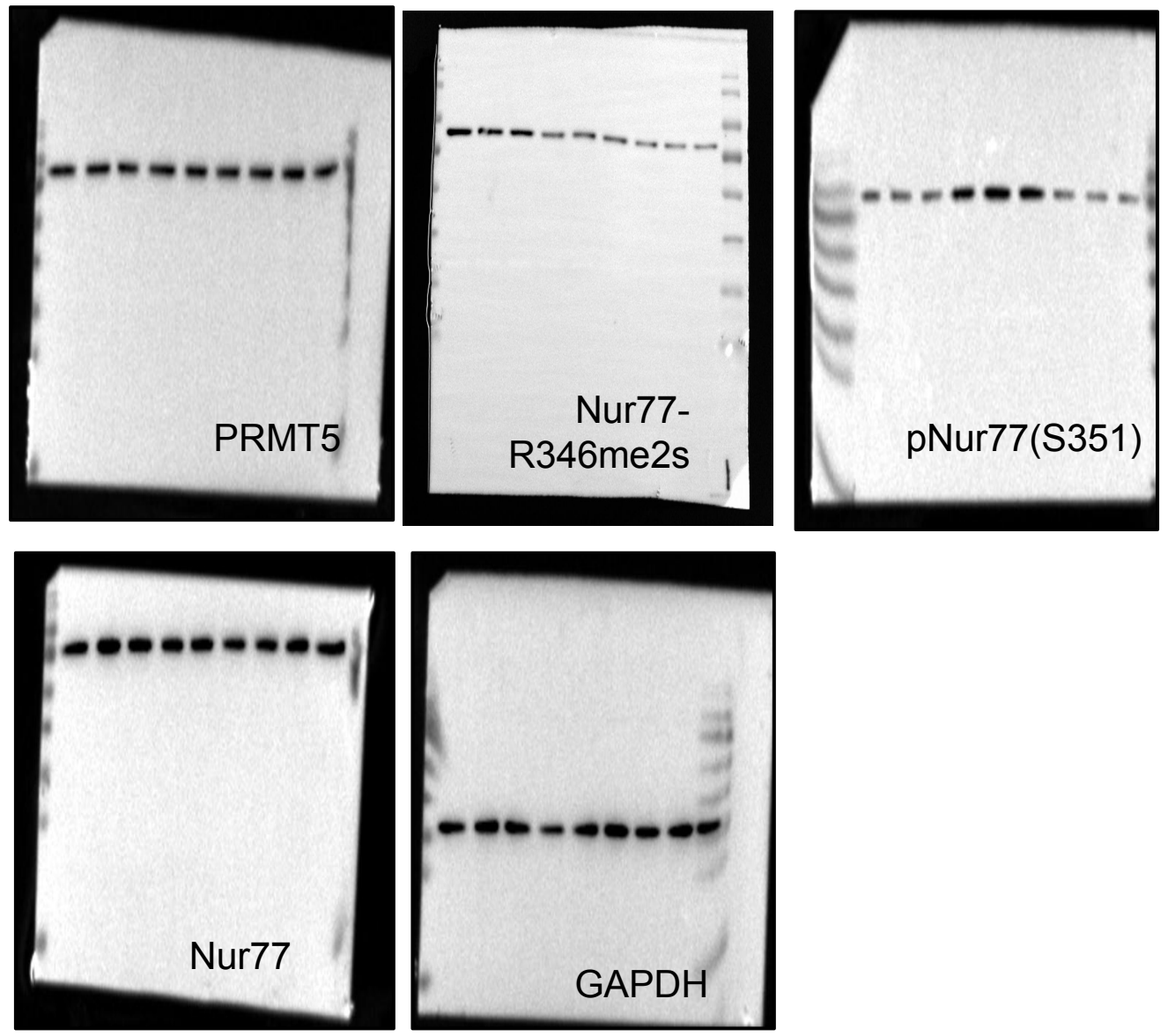

Supplemental Fig. 7A

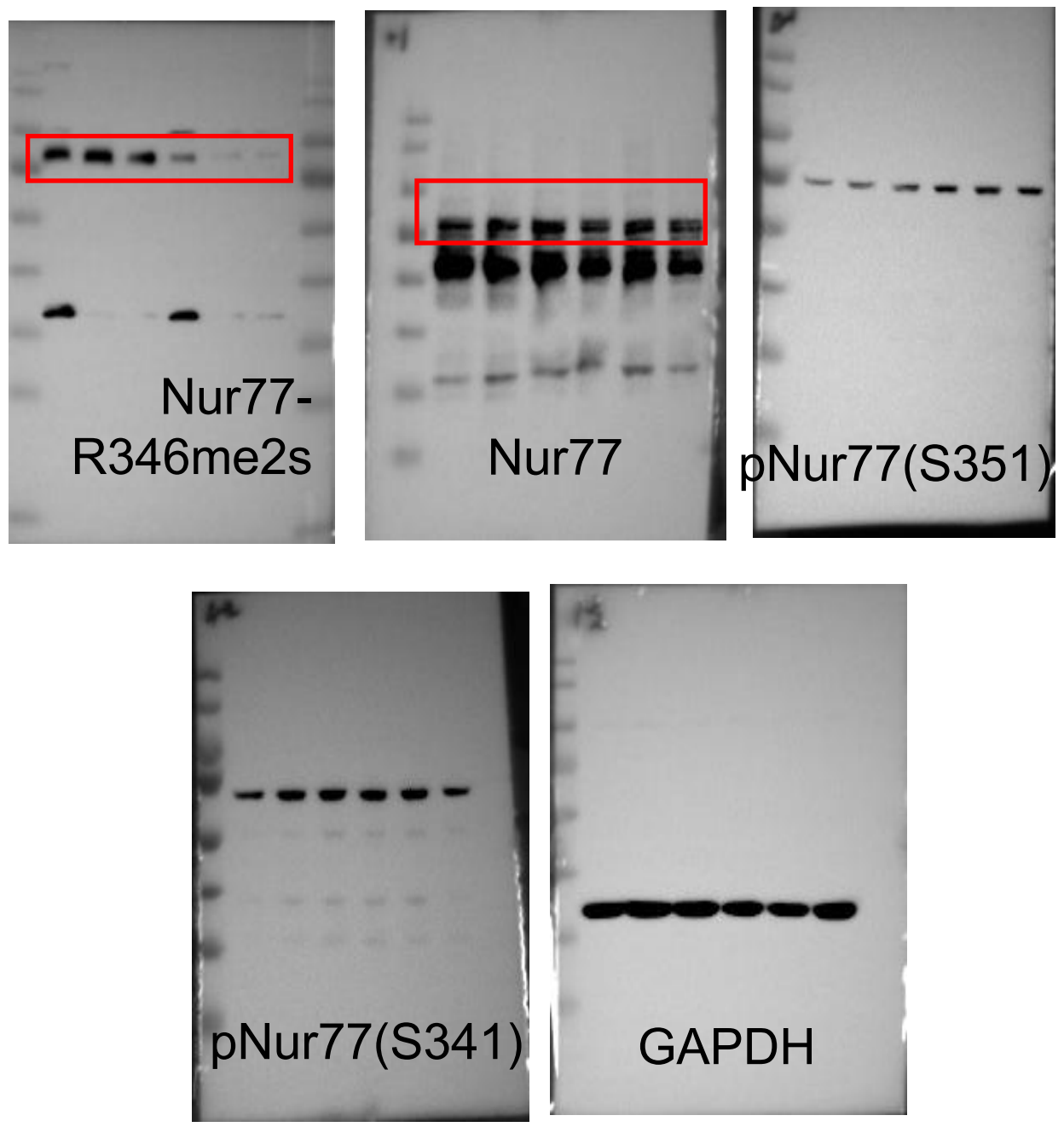

Fig8.B

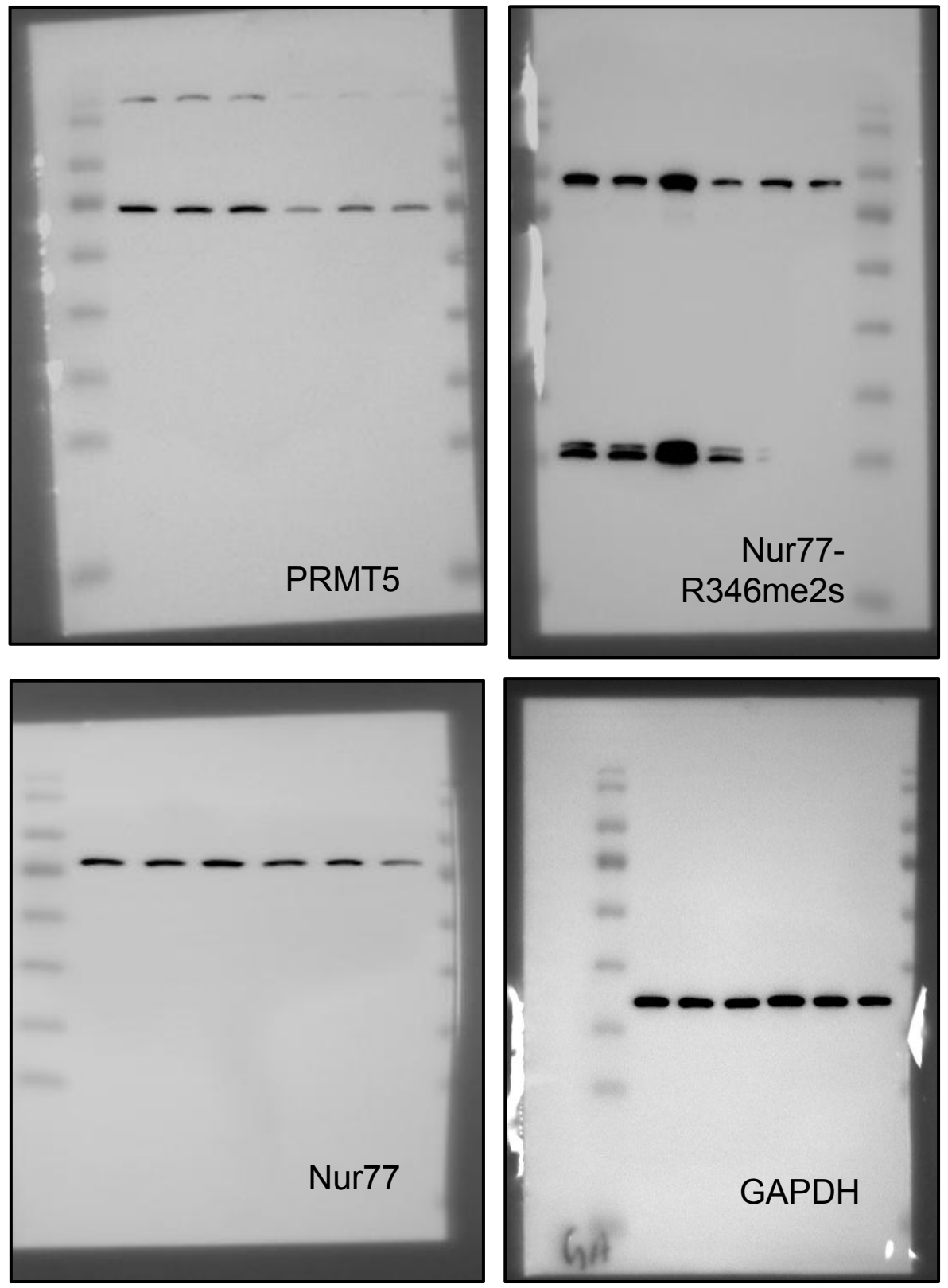
PRMT5
Nur77-
R346me2s
Nur77
GAPDH

Fig9. A

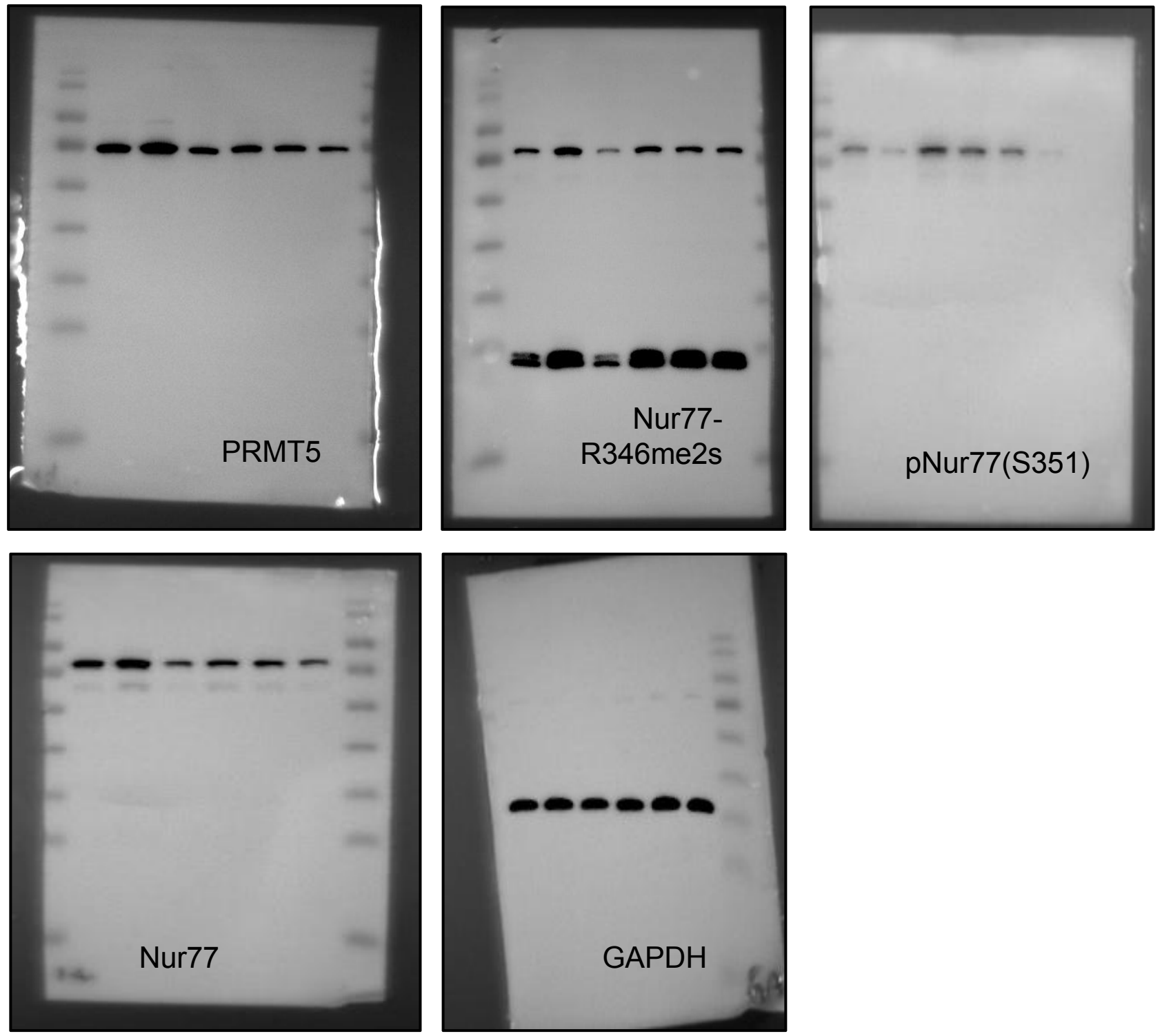
PRMT5
Nur77-
R346me2s
pNur77(S351)
Nur77
GAPDH
